# Genome-wide association study reveals *GmFulb* as candidate gene for maturity time and reproductive length in soybeans (Glycine max)

**DOI:** 10.1371/journal.pone.0294123

**Published:** 2024-01-19

**Authors:** Diana M. Escamilla, Nicholas Dietz, Kristin Bilyeu, Karen Hudson, Katy Martin Rainey

**Affiliations:** 1 Department of Agronomy, Purdue University, West Lafayette, Indiana, United States of America; 2 Division of Plant Science and Technology, University of Missouri, Columbia, Missouri, United States of America; 3 Plant Genetics Research Unit, United States Department of Agriculture (USDA)−Agricultural Research Service (ARS), Columbia, Missouri, United States of America; 4 USDA-ARS Crop Production and Pest Control Research Unit, West Lafayette, Indiana, United States of America; Texas Tech University, UNITED STATES

## Abstract

The ability of soybean [*Glycine max* (L.) Merr.] to adapt to different latitudes is attributed to genetic variation in major *E* genes and quantitative trait loci (QTLs) determining flowering time (R1), maturity (R8), and reproductive length (RL). Fully revealing the genetic basis of R1, R8, and RL in soybeans is necessary to enhance genetic gains in soybean yield improvement. Here, we performed a genome-wide association analysis (GWA) with 31,689 single nucleotide polymorphisms (SNPs) to detect novel loci for R1, R8, and RL using a soybean panel of 329 accessions with the same genotype for three major *E* genes (*e1-as/E2/E3*). The studied accessions were grown in nine environments and observed for R1, R8 and RL in all environments. This study identified two stable peaks on Chr 4, simultaneously controlling R8 and RL. In addition, we identified a third peak on Chr 10 controlling R1. Association peaks overlap with previously reported QTLs for R1, R8, and RL. Considering the alternative alleles, significant SNPs caused RL to be two days shorter, R1 two days later and R8 two days earlier, respectively. We identified association peaks acting independently over R1 and R8, suggesting that trait-specific minor effect loci are also involved in controlling R1 and R8. From the 111 genes highly associated with the three peaks detected in this study, we selected six candidate genes as the most likely cause of R1, R8, and RL variation. High correspondence was observed between a modifying variant SNP at position 04:39294836 in *GmFulb* and an association peak on Chr 4. Further studies using map-based cloning and fine mapping are necessary to elucidate the role of the candidates we identified for soybean maturity and adaptation to different latitudes and to be effectively used in the marker-assisted breeding of cultivars with optimal yield-related traits.

## Introduction

Soybean’s (*Glycine max*) high protein, oil, and carbohydrate content make it a valuable crop worldwide, with various applications in many industries [1–3]. Soybean plants are photoperiod-sensitive and flower under short-day conditions. Soybeans grow across 50°N to 35°S latitudes, and the critical day length period for flowering decreases progressively from higher to lower latitudes [4–8]. Soybean varieties are classified into 13 maturity groups (MG000 to MGX) based on their region of adaptation and day length requirements [8, 9]. When grown outside the optimal latitudinal range, soybeans will flower and mature late in higher latitudes or too early in lower latitudes, reducing biomass and yields [8, 9]. Within each latitudinal range, or region, there is also variation in the length of the growing season, and soybeans are classified as early, mid-, or full season [9].

The change from vegetative to reproductive growth and then to seed maturation are critical developmental switches in soybean influenced and determined by genotype, photoperiod, temperature, elevation, and management [10]. Identifying and understanding the molecular mechanisms and genetic architecture underlying flowering and maturity time is crucial for improving soybean adaptation and yield across differing and variable environments. A number of ‘*E* genes’ and quantitative trait loci (QTLs) contribute to flowering and maturity time through a photoperiod mediated response with different allelic combinations of *E* genes determining soybean adaptation to specific latitudes [8, 11–13]. Eleven maturity loci, *E1* to *E11*, have been reported to control flowering and maturity. Soybean photoperiod sensitivity decreases with the number of recessive alleles [8], while dominant alleles confer late flowering and late maturity except for *E6/J* and *E9* genes [13, 14]. Soybean stem growth also plays a crucial role in flowering time and maturity. The two known genes regulating stem growth are *Dt1*, with the *Dt1Dt1* genotype producing indeterminate stem growth, and *Dt2*, with the *Dt2Dt2* genotype producing semi-determinate plants in the presence of the genotype *Dt1Dt1*. In contrast, the *dt2dt2* genotype produces indeterminate stem growth in the presence of *Dt1Dt1*; however, if the *dt1dt1* genotype is present, the phenotype is determinate [12, 15, 16].

*E1* and *E2* genes delay flowering by suppressing the expression of *GmFT2a* and *GmFT5a*, which are flowering inducers [10, 17–21]. In addition to the *E1* locus, there are two homologous genes, *E1la* and *E1lb*, which repress the expression of *GmFT2a* and *GmFT5a* independently of *E1* [21, 22]. Known variant missense and nonfunctional alleles for the *E1* gene include the *e1-as*, *e1-b3a*, *e1-re*, *e1-fs*, *e1-p*, and *e1-nl* alleles [23–25]. *E3* and *E4* are phytochrome A genes sensitive to red-to-far-red light ratios that promote late flowering under long-day conditions by regulating the expression of *E1* and suppressing *GmFT2a* and *GmFT5a* [22, 23, 26–29]. The *E3* locus has two functional (*E3-Ha* and *E3-Mi*) and three nonfunctional (*e3-tr*, *e3-ns*, and *e3-fs*) alleles described [26, 29] and the *E4* locus has one functional and five nonfunctional alleles (*e4*, *e4-oto*, *e4-tsu*, *e4-kam*, and *e4-kes*) described [27, 28]. The *E5* locus has not been mapped [30, 31]. *E6* and *J* are alleles of the same locus; where *E6* promotes early flowering, and *J* confers a long-juvenile phenotype by delaying flowering under short-day conditions [7, 32–34]. There is not yet a clear understanding of the molecular mechanism for flowering regulation at the *E7* and *E8* is *E1Lb* loci [21, 35–38]. *E9* and *E10* are flowering loci *GmFT2a* and *GmFT4*, respectively, with dominant genotypes promoting earlier flowering than the recessive genotypes [13, 39]. The most recently-reported maturity gene is *E11* which induces earlier flowering when a single dominant allele is present [40].

Days to flowering (R1) and maturity (R8), and duration of flowering to maturity (RL) are critical traits determining soybean adaptation, seed quality, and yield [11, 41, 42]. Most studies focus on understanding the impact of *E* genes on R1 and R8, while there are few studies on the impact of *E* genes on post-flowering and RL [11, 29, 43]. Maturity genes *E1* to *E4* have the most significant effect on R1, R8, and photoperiod sensitivity, controlling as much as 62–66% of the variation in R1 [24, 44, 45]. *E1* has a major effect on R1 and is a crucial regulator of flowering in soybean [46]. Studies on the allelic variation of *E1* to *E4* in different soybean populations reveal a complex genetic control underlying R1 and suggest the existence of unknown genes with roles in R1 [8, 24, 29, 44, 47, 48]. Many other QTLs that have been discovered show evidence of influencing R1 and R8; however, based on their map position, some correspond to the known *E* genes [41, 43, 49].

An effective way to detect associations between single nucleotide polymorphisms (SNPs) and the trait of interest is through genome-wide association studies (GWAS). GWAS commonly estimate the marginal effect of individual SNPs by single-locus analysis, which only detects the SNPs that have relatively large effects, leaving out the SNPs with minor effects [50–54]. Fully uncovering the genetic architecture of important traits is a complicated task that requires careful consideration of the study design, testing populations, data collection, and analysis. At the same time, there is evidence that major-effect loci are less influenced by genetic background than minor-effect loci [55], and it is probable that major *E* genes with large effects on R1 and R8 alter, mask or hide the effect of unknown minor *E* genes. In order to understand the genetic regulation of R1 and R8 beyond what is already known for the eleven major genes, researchers studied causal genetic interactions through epistasis and populations sharing the same genotype for major *E* genes [54, 56, 57]; however, the molecular function of R1- and R8- related genes is still elusive with many unknown loci yet to discover.

Although there is much progress in identifying new maturity loci and understanding the genetic architecture of R1, R8, and RL, the known loci explain only a portion of variation, leading many to question how to explain the remaining variation. Identifying novel loci for R1, R8 and RL may improve our understanding of soybean adaptation to different latitudes, which will facilitate maximizing yield for specific environments. Here, we proposed to investigate the genetic architecture of R1, R8, and RL beyond major E genes (*E1* to *E3*) by high-throughput genetic markers and association analysis on a soybean panel from MG III and IV sharing the same allelic combination ‘*e1-as/E2/E3’* for major genes.

## Materials and methods

### Plant material and experimental design

**Fig 1:**
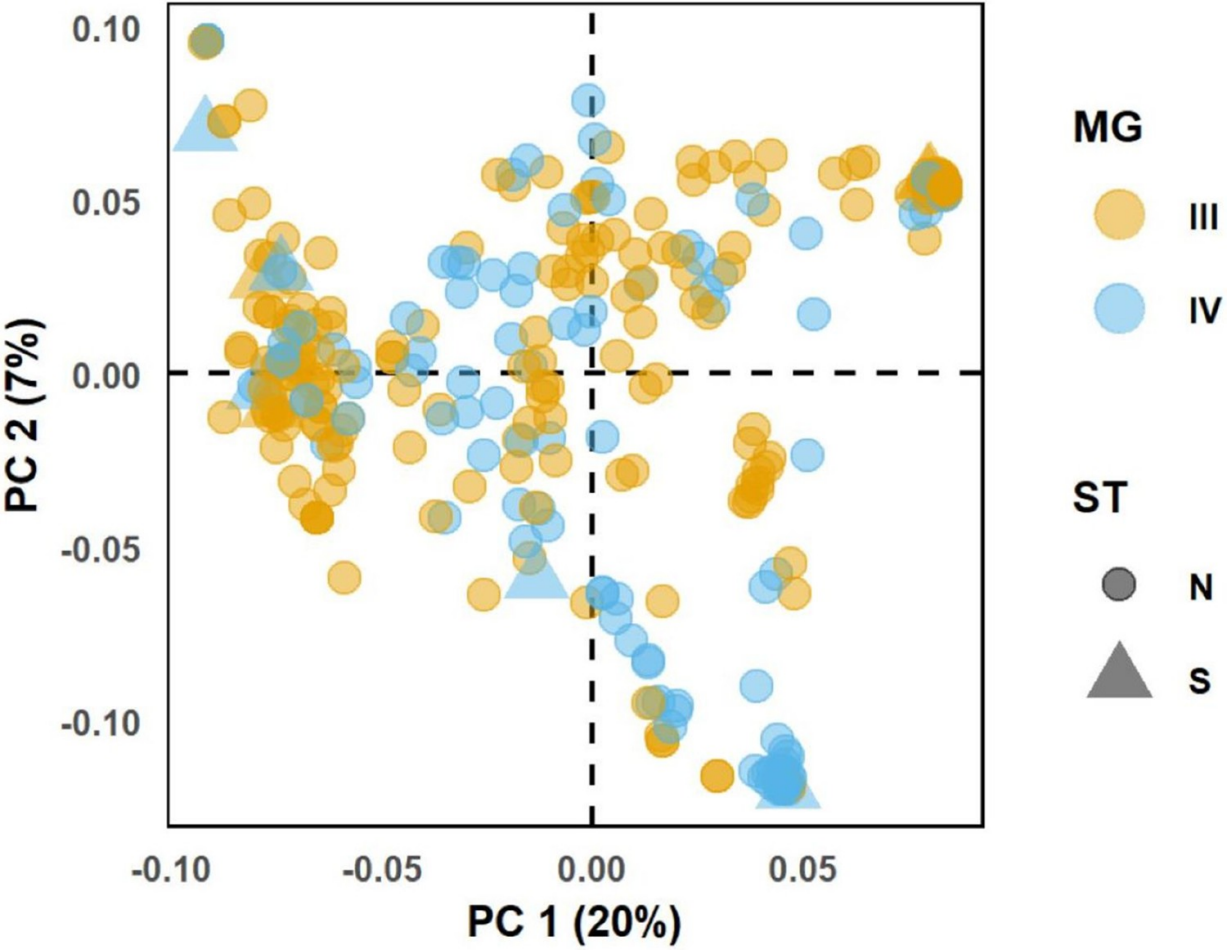
Principal component analysis (PCA) biplot of three hundred twenty-nine *G*. max USDA accessions. Individuals are represented with different colors and shapes according to maturity group (MG) and stem termination type (ST). N-indeterminate, S- semideterminate.

To assemble the diversity panel, we selected 329 soybean accessions predicted to share the same allelic combination for major *E* genes, *e1-as/E2/E3*, from the soybean germplasm collection using the GRIN data explorer (https://www.soybase.org/grindata/). This tool facilitates searches of the GRIN descriptor data [58]. The *e1-as* allele is a missense, recessive allele for the *E1* gene [23], while *E2* and *E3* are functional alleles. The selected 329 accessions were from MG III and IV and include 220 improved cultivars and 109 breeding lines with either indeterminate (*Dt1Dt1/dt2dt2*) or semi-determinate (*Dt1Dt1/Dt2Dt2*) stem growth and predicted to be resistant to shattering (Fig 1). Descriptor data available in the GRIN data explorer for shattering and *E1*, *E2*, and *E3* genes, were imputed. The alleles at the shattering (*Pdh1*) locus were imputed in a previous study performing a genome-wide association study (GWAS) using the *Pdh1* allele status as a phenotype [59]. They identified a highly associated marker in the SoySNP50K array that they later used to predict the *Pdh1* allele status in the GRIN collection. To predict the *E1*, *E2*, and *E*3 genes, researchers studied a genomic dataset of 406 soybean genotypes, including cultivars, landraces, and wild species (*Glycine soja*) [58]. They utilized GWAS and direct genotype information to select associated markers that could accurately predict the allele status of *E1*, *E2*, and *E3* in the GRIN collection.

We grew all accessions in single row plots in Indiana (ACRE-IN) and Missouri (Columbia-MO) in 2017 and 2018. In 2017, single row plots were 0.3 m long with 0.3 m spacing among plots and 10 seeds per plot. The single row plots in 2018 were 1.83 m in length with 0.72 m spacing among plots and 50 seeds per plot. To control for potential heterogeneity of experimental units, we evenly distributed maturity checks, which included 194Dremut #3021, DSN11-03148 (MGIV), DSN11-06152 (MGIII), DSN11-12073 (MGII), and IA3023 (MGIII), over the field experiment area for each location to facilitate spatial correction of field plot variation. In 2019 and 2020, we grew the accessions in four-row plots, two locations (ACRE Farm, West Lafayette, IN, and Ag Alumni Seeds, Romney, IN), and two replications in a completely randomized block design (CRBD) with replications as blocks; with only one replication grown at Romney in 2020. The four row plots were 1.83 m in length and 0.76 m spacing among rows, at a density of approximately 36 plants m^-2^. In 2020, GDM seeds in Gibson, IL, planted all accessions in two row plots of 5 m long and 0.72 m spacing among rows with three replications in a CRBD, at a density of approximately 38 plants m^-2^. Geographical coordinates of the four experimental locations are: 40° 28’ 20.5’’ N and -86° 59’ 32.3’’ W (Acre-IN), 40° 14’ 09.2’’ N and -86° 53’ 24.5’’ W (Romney-IN), 40° 28’ 22.944" N and -88° 20’ 43.152" W (Gibson- IL); 38° 54’ 50.63" N and -92° 17’ 33.67221" (Columbia-MO). S1 Fig shows a map of the experimental locations and delimitating MG III and IV zones that we elaborated using the R package “ggplot2” [60]. Combination of years and locations as unique environments resulted in nine environments. S1 Table presents the planting dates for individual environments.

We observed the plots for R1, R8, and RL in all environments; R1 was recorded as the number of days from planting until 50% of the plants in a plot had one open flower at any node on the main stem, R8 was defined as the number of days after planting when 95% of pods had reached maturity color, and RL was calculated as the difference between R8 and R1 [61]. We visited the locations for ground visual dating of R1 as soon as the earliest flowering plots in each location began to show flowers and for R8 as soon as the earliest maturating plots in a given location began to senesce. Visits were every two to three days, and we interpolated dates when it was clear a plot reached R8 or R1 between visits. We expressed the date of plot flowering and maturity as the number of days after planting (DAP).

### Genotypic data, quality control and linkage disequilibrium estimation

The complete USDA soybean germplasm collection has genotype data of 42,509 SNPs (http://www.soybase.org/dlpages/#snp50k) obtained by genotyping with the Illumina Infinium SoySNP50K iSelect Bead chip [62]. Our colleagues [63] retrieved and made available the genotypic data from the SoyBase website [64], coded as {0,1,2}, imputed for missing data, and cleaned for redundant SNPs and SNPs with MAF≤ 0.05. The coded allelic genotype as {0,1,2} corresponded to {AA, Aa, aa}; where AA is homozygous towards the reference genome Williams82 [63]. We performed principal component analysis (PCA) of whole-genome SNPs using R software and plotted the first two principal components (PC) for visualization (Fig 1). We obtained the genomic relationship matrix (GRM) by using the R package NAM and visualized it with a Heatmap (S2 Fig) [65].

To characterize the mapping resolution, the average extent of genome-wide LD between pairwise SNPs was estimated by euchromatin and heterochromatin regions. First, we calculated the squared correlation coefficient (*r*^*2*^) of alleles between markers using the LD function in the R package NAM [65], which phases molecular markers using the expectation-maximization algorithm [66]. Due to differences in recombination rates between euchromatic and heterochromatic regions, we calculated *r*^*2*^ separately for the two chromosomal regions; We defined the approximate physical length and positions of heterochromatic and euchromatic regions in the Wm82.a2.v1 as described previously [67]. To measure the extent of LD, we only used the *r*^*2*^ between SNPs with pairwise physical distances smaller than 10 Mb in either the euchromatic or heterochromatic region of each chromosome. We computed the mean *r*^*2*^ within intervals of 100 kb and drew the LD decay plots (S3 Fig); then, we calculated the LD decay rate by chromosome as the distance where the average *r*^*2*^ dropped to half its maximum value [68].

### Data and association analysis

To minimize the effect of environmental variation, we estimated the genetic values as the Best Linear Unbiased Estimator (BLUE) for each line by a mixed model analysis using the R package ‘lme4’ [69]. We combined year and location factors into one environment term (Eq 1) (S1 Table). The linear model used to model genetic values was:

$$y_{ijk}=\mu +f(x)+\alpha_{i}+\beta_{j}+\gamma_{k}+e_{ijk}$$ (1)

Where *y*_*ijk*_ is the phenotype (R1, R8, RL) measured in the *j*th environment into the *k*th block, *μ* is the intercept, *f*(*x*) is the spatial covariate based on a moving-average of neighbor plots as described by [70] and estimated through functions NNscr/NNcov of R package NAM [65], *α*_*i*_ captures the fixed genotype effects, *β*_*j*_ (*j* = 1,…number of environments) is the random environment effect with $\beta_{j}∼N(0,\sigma_{\beta }^{2})$ where $\sigma_{\beta }^{2}$ is the environment variance, *γ*_*k*_ is the random effect of the *k*th block with $\gamma_{k(j)}∼N(0,\sigma_{\gamma }^{2})$ where $\sigma_{\gamma }^{2}$ is the block variance, and *e* is the residual term distributed as $e_{ijk}∼N(0,\sigma_{e}^{2})$ where $\sigma_{e}^{2}$ is the residual variance. We estimated the BLUEs for individual years, environments, and across all years and environments by modifying Eq 1. To estimate broad sense heritability (H) on an entry-mean basis, we used the same model structure from Eq 1 with only the spatial covariate treated as fixed effect; while, the accessions were treated as random effect and assumed to be normally distributed as *α*_*i*_ ~ N (0, $\sigma_{\alpha }^{2}$) where $\sigma_{\alpha }^{2}$ is the genetic variance. We estimated H from the REML variance components as:

$$H=\frac{\sigma_{\alpha }^{2}}{\sigma_{\alpha }^{2}+\frac{\sigma_{e}^{2}}{r}}$$ (2)

Where *H* corresponds to broad sense heritability in mean entry bases, σ^2^_*α*_ is genetic variance, σ^2^_*e*_ corresponds to variance of error, and r is the number of replications. To estimate narrow-sense heritability (h^2^) we used a whole-genome regression using the expectation-maximization restricted maximum likelihood method from the “NAM” package [65]. In this model the accessions BLUPs were modeled as a function of the polygenic effects $g∼N(0,{K\sigma }_{g}^{2})$, where $\sigma_{g}^{2}$ is the additive genetic variance and **K** is a matrix of kinship coefficients estimated by genomic data, and the residual term $e∼N(0,\sigma_{e}^{2})$ where $\sigma_{e}^{2}$ is the residual variance. We estimated h^2^ from the variance components as:

$$h^{2}=\frac{\sigma_{g}^{2}}{\sigma_{g}^{2}+\sigma_{e}^{2}}$$ (3)

To conduct the GWAS, we used an empirical Bayesian framework using the R package NAM, which uses a sliding-window strategy to increase power and avoid double fitting markers into the model [65]. For each trait, we used the BLUEs from Eq 1 as phenotypes. The model includes a polygenic term that accounts for the population structure to minimize false positives and increase statistical power. We tested marker-trait associations using a linear model implemented in the function *gwas*2:

y=μ+Zu+g+e (4)

Where the BLUEs (**y**) obtained in Eq 1 were modeled as a function of an intercept (μ), the incidence matrix (**Z**) relating the accessions to the marker effects ***u*** ~ N (0, **I**σ^2^_u_), the vector of independent residuals ***e***~ N (0, **I**σ^2^_*e*_), and the polygenic effect ***g*** ~ N (0, **K**σ^2^_*g*_) accounting for the genetic covariance among individual through the genomic relationship matrix **K**. Statistical significance of markers was measured by calculating the likelihood ratio test statistics (LRT), which represent the improvement that each SNP provides to a mixed model when compared to the reduced model without the marker. The p-values were obtained from LRT using the Chi-squared density function with 0.5 degrees of freedom [65]. We used the multiple testing correction developed by [71] to evaluate marker trait associations. The corrected p-value threshold (0.000068) was obtained by applying a Bonferroni correction to the significance level (p-value 0.01) using the number of independent tests (143). The number of independent tests corresponded to the number of principal components accounting for a large portion of the genotypic variance (95%). We used the R package CM-plot to create the Manhattan plots [72] and the R package “ggplot2” to generate the plots of the genomic regions with the stronger signals [60].

We further inspected seven association signals to identify their potential functional effects. Based on Wm82.a2.v1 genome assembly, selected SNPs are positioned in Chr 4 at 17,075,267 (G/A) bp, 17,228,343 (T/C) bp, 40,009,617 (C/T) bp, 40,276,263 (A/G), 40,151,473 (T/C) bp and 40,218,961 (G/A) bp and in Chr 10 at 41,455,680 (G/A) bp (Table 1). In GWAS, the strongest associated variants are rarely the causal mutation (CM); instead, they are likely in linkage disequilibrium (LD) with the causal variant, especially when using low-density genotype data [68, 73].

**Table 1:** Summary of single-nucleotide polymorphisms (SNPs) significantly associated with flowering time (R1), maturity time (R8), and reproductive length (RL) in three hundred twenty-nine *G*. *max* accessions across environments and years.

Chr	SNP Wm82.a2^a^	SoySNP50k_ID ^a^	Trait	Peak	-log(p-values)	Effect	Reported QTLs^a^
**10**	Gm10_41455680_G_A*	ss715607080	R1	Peak-3	4.4	1.79	R8 full maturity 10-g4.1, Reproductive stage length 4-g2.1, Reproductive stage length 4-g2.2, Reproductive period 3-g5, Seed protein 7-g7 and Seed weight 4-g10
**4**	Gm04_17228343_T_C*	ss715587206	R8	Peak-1	4.71	-1.85	^-^
**4**	Gm04_17075267_G_A*	ss715587203	R8	Peak-1	4.4	-1.77	-
**4**	Gm04_40009617_C_T*	ss715587845	R8	Peak-2	4.66	-1.77	Reproductive stage length 2–2, 3–4, and 5–1, Pod maturity 18–3^b^
**4**	Gm04_40276263_A_G*	ss715587857	R8	Peak-2	4.7	-1.76	^b^
**4**	Gm04_16673792_G_A	ss715587192	R8	Peak-1	4.4	-1.77	-
**4**	Gm04_16889396_T_C	ss715587197	R8	Peak-1	4.4	-1.77	-
**20**	Gm20_46615517_A_C	ss715638773	R8	Peak-4	4.33	-1.36	First flower 6-g4, R8 full maturity 8-g13, Reproductive period 3-g1
**4**	Gm04_39977826_G_A	ss715587844	R8	Peak-2	4.17	-1.6	^b^
**4**	Gm04_40151473_T_C*	ss715587852	R8	Peak-2	4.18	-1.68	^b^
**4**	Gm04_40218961_G_A*	ss715587856	R8	Peak-2	4.18	-1.68	^b^
**3**	Gm03_36427644_C_T	ss715585727	RL	Peak-5	4.17	-1.62	First flower 4-g10, First flower 3-g2, R8 full maturity 3-g3
**4**	Gm04_40151473_T_C*	ss715587852	RL	Peak-2	5.63	-1.92	^b^
**4**	Gm04_40218961_G_A*	ss715587856	RL	Peak-2	5.63	-1.92	^b^
**4**	Gm04_17228343_T_C*	ss715587206	RL	Peak-1	4.24	-1.68	-
**4**	Gm04_40009617_C_T*	ss715587845	RL	Peak-2	4.35	-1.65	^b^
**4**	Gm04_39006019_T_C	ss715587808	RL	Peak-2	4.22	-1.58	^b^
**4**	Gm04_39484148_T_C	ss715587823	RL	Peak-2	4.22	-1.58	^b^
**4**	Gm04_39731223_A_G	ss715587834	RL	Peak-2	4.22	-1.58	^b^

SNP Wm82.a2 is the SNP positions based on Wm82.a2

^a^ Information obtained from SoyBase and scientific reports

^b^ the same reported QTLs applied for all SNPs withing peak 2 in Chr4

* Positions of the tagging SNPs used for candidate gene selection. SNP IDs, location in the genome, variance explained, distance from known *E* genes and chromosomal region are presented in an expanded version of this table in S6 Table

We followed three steps to identify potential causal genes: First, we identified an initial list of potential causal genes by using the Synthetic Phenotype to CM strategy (SP2CM) developed by our colleagues [73] and implemented in AccuTool (https://soykb.org/AccuTool/index.php). In SP2CM and AccuTool, the positions of the SNPs significantly associated with the traits were treated as synthetic phenotypes, and their correspondence with other genomic variants within a specified region was measured through an average accuracy estimate, whose equations are described by [73]. An average accuracy of 100% indicates an exact match between the significant SNP and the other genomic variant in the Soy775 accession panel. The Soy775 accession panel combines all publicly available resequencing data from 775 soybean accessions, which increases AccuTool’s power of detection [73]. We explored a window of +/- 1 Mb distance from the significant SNPs. Genes with genomic variants that are in high correspondence (average accuracy >85%) with significant SNPs were selected as potential candidate genes. According to our colleagues [73] using a correspondence threshold of 85% and a large interval reduces the risk of missing true positive associations without negative consequences on the analysis as any variant outside the range of LD will show low correspondence with the significant SNPs. AccuTool uses SNP positions and annotated genes from the Williams 82 a2.v1 reference genome, whose source is SoyBase (www.soybase.org). Second, we retrieved the previously characterized functions of the list of potential causal genes generated in the first step. To determine the gene’s functions, we searched SoyBase, NCBI RefSeq, UniProt, The *Arabidopsis* Information Resource (TAIR), and scientific articles. Lastly, we selected a final list of genes that meet at least one of the two following criteria: 1) genes have a known function in soybean related to the studied traits; 2) genes have orthologs in *Arabidopsis* or other plants, whose functions are related to the studied traits.

## Results

### Phenotypic variation and genetic diversity

Despite sharing the same haplotype for major *E* genes (*e1-as/E2/E3*), the 329 accessions exhibited a wide range of variation in R1, R8, and RL. The phenotypic distribution of R1, R8, and RL by environment is presented in S4 Fig. Across environments, R1 ranged from 31 to 80 DAP, R8 ranged from 110 to 169 DAP, and RL ranged from 55 to 111 days. In addition, R8 and RL were the least variable traits, whereas R1 was the most variable. Descriptive statistics by accessions, environments, and across all environments are provided in S2 and S3 Tables, alongside broad- and narrow-sense heritability. From all environments, Gibson, IL in 2020 (E9) had the highest mean values and widest ranges for R1, R8, and RL; and it was planted earlier compared to the other environments (S1 Table). The observed broad sense heritabilities were 66% for R8, 52% for RL and 61% for R1, while narrow-sense heritabilities were 69% for R8, 57% for RL, and 65% for R1.

To investigate the population structure of the selected 329 accessions, we generated a scatter plot of the first two PCs from PCA of whole-genome SNPs in the 329 accessions (Fig 1), that in combination with k-mean clustering (S5 Fig) indicated the existence of two subgroups in the studied panel of accessions. The GWAS model included a polygenic term that accounts for the population structure to minimize false positives and increase statistical power. A total of 31,689 polymorphic SNPs with MAF ≥ 0.05 were used for the association analysis with an average marker density of 1 SNP every 30 kb genome-wide, and ranging from 21 kb/SNP (Chr 13) to 44 kb/SNP (Chr 1) (S6 Fig). The majority of the SNPs (76%) were located in euchromatic regions (S4 Table). Due to the differences in LD decay rates between chromosomal regions, a higher marker density is required for the low LD euchromatic regions, whereas in the heterochromatic region a lower marker density is acceptable [41, 74]. The average LD decay rate of the panel of accessions in euchromatic regions was 2150 kb, with *r*^2^ = 0.38 being half of its maximum value; while, in heterochromatic regions, the *r*^2^ dropped to half of its maximum until 8650 kb (S5 Table, and S3 Fig). Given the average marker density of 30 kb/SNP and the LD decay rate, the used SNPs were expected to have reasonable power to identify loci significantly associated to the studied traits.

### Genome-wide association analysis

**Fig 2:**
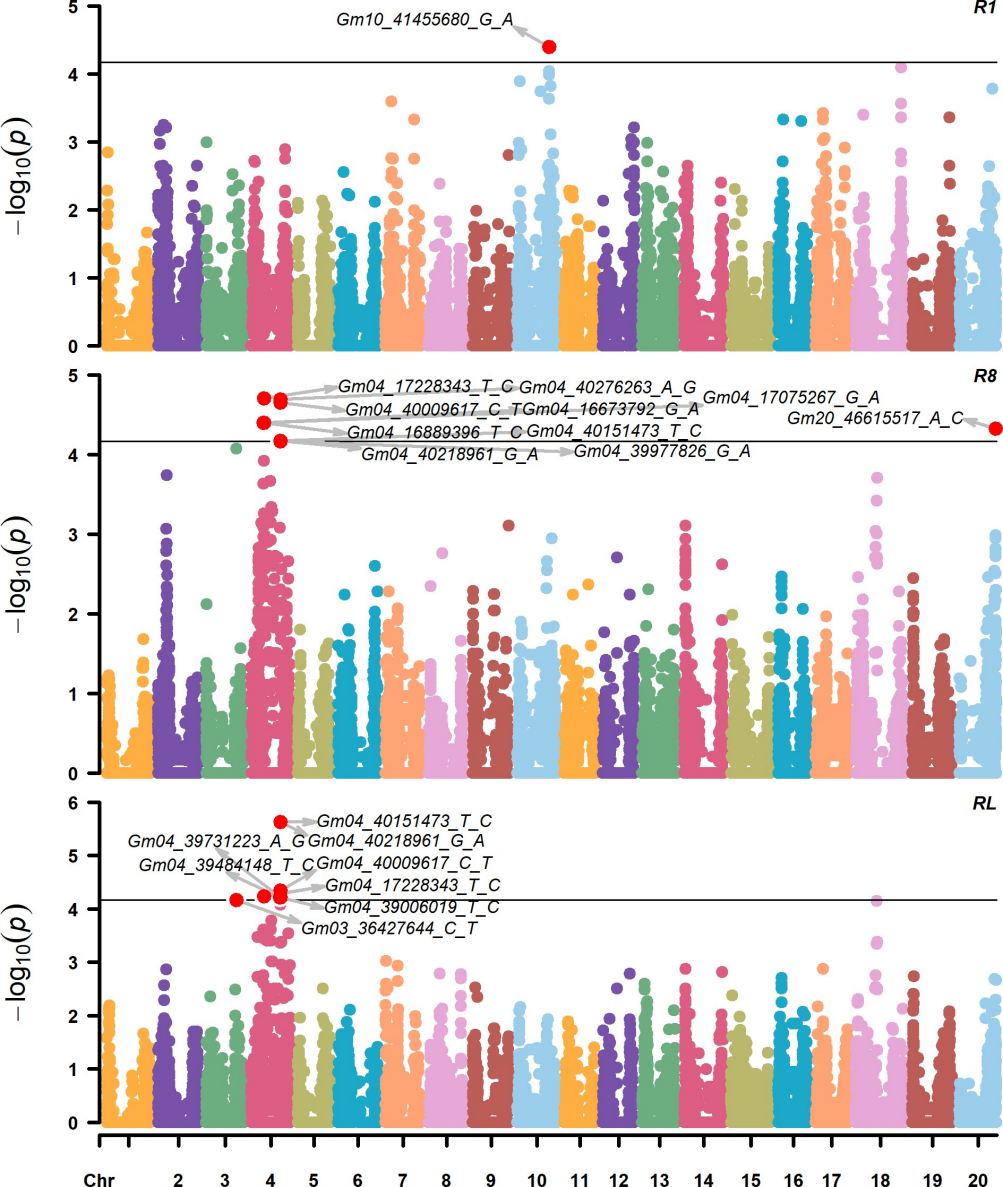
Manhattan plots of GWAS for flowering time (R1), maturity time (R8), and reproductive length (RL) in three hundred twenty-nine *G*. *max* accessions. GWAS results correspond to the analysis across the nine environments. The horizontal dashed lines indicate the statistically significant cut-off of–log (p-value) = 4.16. Significant SNPs IDs correspond to the Wm82.a1.

Successful identification of marker-trait associations and the power of resolution relies on the size and variability of the mapping population [75]. In soybeans, as few as 9,600 SNPs can capture most of the haplotype variation [75, 76]; thus, this study used a very diverse population and a sufficient number of SNPs (Fig 1 and S6 Fig). GWAS across environments revealed a total of 15 SNPs associated with at least one of the phenotypes with *p-*values *<* 6.85 x 10^−5^ (Fig 2). One SNP was associated with R1, ten SNPs with R8, eight SNPs with RL, and four SNPs with R8 and RL. Of the ten SNPs associated with R8, nine were from Chr 4 with two strong peaks, and one was from Chr 20. One SNP associated with RL was from Chr 3, and the other seven SNPs were from Chr 4, with the same two strong peaks observed for R8. The shared significant SNPs between R8 and RL were from Chr 4. The effect of significant SNPs on trait phenotypes ranged from -1.38 to 1.92 DAP. A summary of the association signals with the effect of each SNP on days to R1 and R8, and RL is presented in Table 1 and, an expanded version of the table is presented in S6 Table, including additional information about the significant SNPs such as variance explained, chromosomal region, and SNP ID.

GWAS, by unique environments and years, revealed a total of 5 SNPs associated with R1, 14 SNPs associated with R8, and 13 SNPs associated with RL; ten SNPs were associated with R8 and RL. From them, we identified four, six, and five environment-specific association signals for R1, R8, and RL, respectively. Environment-specific SNPs may be susceptible to environmental influences and could play an essential role in soybean adaptation to specific environments. A summary of the association signals from the different environments and years, alongside the effect and variance explained by each SNP marker for R1, R8, and RL is presented in S7 Table, and S7 Fig. We selected the six most significant SNPs from Chr4 and one from Chr 10 to examine further their surrounding genomic region for genes that can potentially explain their functional effect on the studied traits. Selected SNPs from Chr 4 were significantly associated with R8 and RL across all environments, had the smallest P-values and the highest effects, and were present in more than one environment (S6 and S7 Tables); in addition, we included the SNP on Chr 10 because it was the only significant association observed for R1 across all environments. In this paper, we referred to the selected SNPs as tagging SNPs (Table 1, and S6 Table). To provide further verification of the tagging SNPs, we examined their effect on trait’s performance and observed significant differences between accessions carrying the reference and alternative alleles for all SNPs (S8 Fig).

### Candidate gene exploration

**Fig 3:**
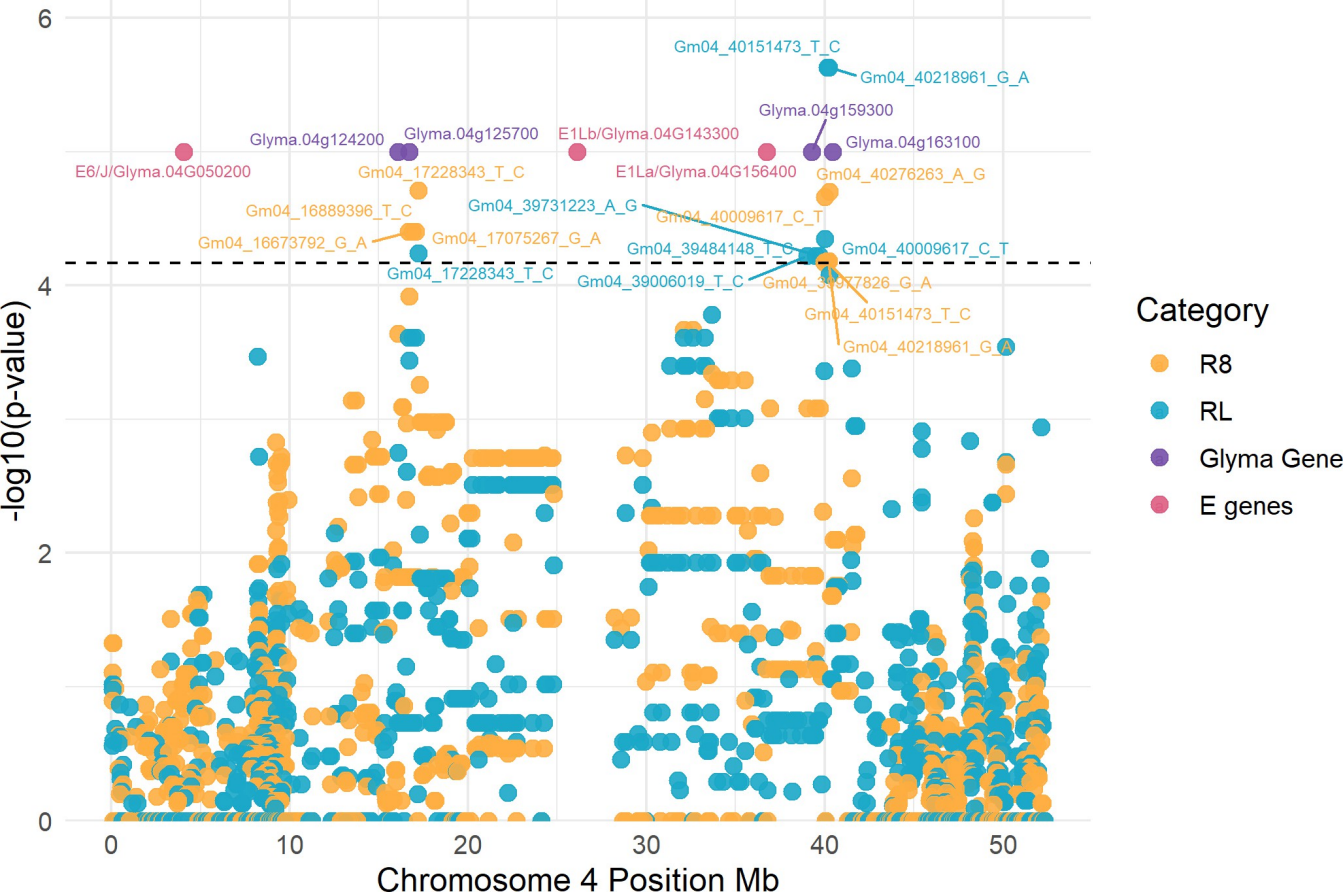
GWAS results showing the candidate genomic regions of peak-1 (left) and peak-2 (right) for R8 (yellow) and RL (blue). In the graph there are labels for the significant SNPs, the known maturity *E* genes (pink), and the potential causal genes in chromosome 4 (purple) which were obtained using AccuTool. The horizontal dashed lines indicate the statistically significant cut-off of–log (p-value) = 4.16. Significant SNPs IDs correspond to the Wm82.a1.

**Fig 4:**
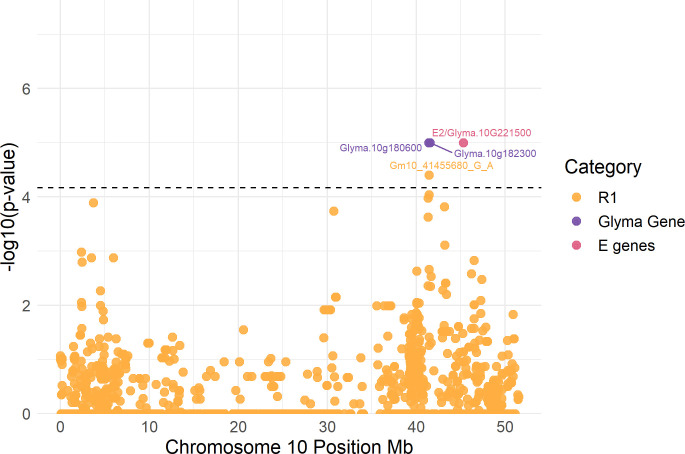
GWAS results showing the candidate genomic region of peak-3 for R1. In the graph there are labels for the significant SNPs, the known maturity E genes (pink), and the potential causal genes in chromosome 10 (Purple), which we obtained using AccuTool. The horizontal dashed lines indicate the statistically significant cut-off of–log (p-value) = 4.16. Significant SNPs IDs correspond to the Wm82.a1.

We explored a genomic region of +/- 1 Mb surrounding the seven tagging SNPs to gain insight into their potential functional effects. Local Manhattan plots, linkage disequilibrium analysis (LD), and SNPs physical positions revealed that the tagging SNPs are likely representing three different association peaks, one on Chr 10 associated with R1 and two on Chr 4 associated with R8 and RL (Figs 3, 4, and S5 Table). We defined the regions of the peaks taking the two most distant significant SNPs of each peak. Based on the Williams 82 a2.v1 reference genome, one peak was on Chr 4 at 16,673,792–17,228,343 bp (peak-1), a second peak was also on Chr 4 but at 39,006,019–40,276,263 bp (peak-2), and a third peak was on Chr 10 at 41,455,680 (peak-3). Of the seven tagging SNPs, two were from peak-1, four from peak-2, and one from peak-3 (Table 1). Although the peak-3 on Chr 10 had only one significant SNP, we decided to examine it further since it was the only significant SNP for R1 and had an effect of +/- 2 DAP on flowering time (Table 1, and S6 Table). There were four previously reported QTLs, three for RL and one for R8 in the genomic region of peak-2 on Chr 4 [43, 57, 77, 78]. Similarly, there were four previously reported QTLs in peak-3 on Chr 10, one for R8 and three for RL according to the SoyBase.org browser and previous studies (Table 1) [79, 80]. Significant peaks on Chr 4 are at least 2 and 9 Mb apart from maturity genes *E1La*, *E1Lb*, respectively, whereas the significant SNP on Chr 10 is 4 Mb away from maturity gene *E2* (Table 1, and Figs 3 and 4).

To explore the genomic region of +/- 1 Mb around the tagging SNPs, we used the SP2CM implemented in AccuTool [73], which measures the correspondence of the tagging SNPs with other genomic variants within the specified region through an average accuracy estimate. This methodology allows the assessment of the relationship between tagging SNPs and variant alleles of nearby genes. Variant alleles in a total of 53 *Glyma* genes showed high correspondence (average accuracy > 85%) with the tagging SNPs of peak-1, variant alleles of 46 *Glyma* genes had high correspondence with the tagging SNPs of peak-2, and variant alleles of 12 *Glyma* genes had high correspondence with the tagging SNPs of peak-3. We designated six genes as potential candidates, with two genes per peak. The selected genes are interesting because they had orthologs in *Arabidopsis* and other plant species with functions related to flowering and maturity, even though their exact functions in soybeans are unknown (Table 2). For each gene, we selected the variant allele with the highest correspondence with tagging SNPs as the most likely cause of the effect on trait phenotypes (Table 2). From them, one was a modifying variant at nucleotide 113 (A to T) in *GmFulb* (*Glyma*.*04G159300*) which caused an amino acid change (H to L) [77]; the gene mentioned above is the most interesting candidate of chromosome 4. Local Manhattan plots of the genomic regions showing the association signals on Chr 4 and 10, nearby known *E* genes, significant SNPs, and potential candidate genes are presented in Figs 3 and 4. A complete list of genes that had high correspondence with the different tagging SNPs is presented in S8 Table.

**Table 2:** Description of the candidate genes.

Gene ID	Chr	Variant^a^	Variant Position^b^	SNP Wm82.a2^b^	Peak	Avg Acc %^c^	Distance (Kb)^d^	Description^e^
Glyma.04g124200	4	AT|upstream_gene_variant	16087021	Gm04_17228343_T_C*	1	91.1	1141.32	BZIP transcription factor/ABA-responsive element binding protein 3
				Gm04_16673792_G_A		87.8	586.77	
				Gm04_16889396_T_C		87.2	802.38	
				Gm04_17075267_G_A*		86.8	988.25	
Glyma.04g125700	4	C|upstream_gene_variant	16707992	Gm04_17075267_G_A*	1	96.7	367.27	Myb domain protein 33
				Gm04_17228343_T_C*		93.3	520.351	
				Gm04_16673792_G_A		100	34.2	
				Gm04_16889396_T_C		98.2	181.40	
Glyma.04g159300	4	A|missense_variant|H38L	39294836	Gm04_40218961_G_A*	2	90.2	924.12	^f^*GmFulb* an *AP1/FUL-*like gene that are mainly involved in reproductive transition, floral meristem identity, and fruit development in plants
				Gm04_40151473_T_C*		89.3	856.64	
				Gm04_39006019_T_C		97.7	288.82	
				Gm04_39484148_T_C		99.6	189.31	
				Gm04_39731223_A_G		99.6	436.39	
Glyma.04g163100	4	G|intron_variant	40436493	Gm04_40218961_G_A*	2	93.9	217.53	PHD finger-containing protein. Interacts with BDT1, acts with other PHD proteins to associate with flowering genes and thereby suppress their transcription.
				Gm04_40276263_A_G*		97.3	160.23	
				Gm04_40151473_T_C*		92.5	285.02	
				Gm04_39484148_T_C		87.7	925.34	
				Gm04_39731223_A_G		87.7	705.27	
Glyma.10g180600	10	C|upstream_gene_variant	41423808	Gm10_41455680_G_A*	3	96.7	1019.19	GmCRY2/Cryptochrome 2
Glyma.10g182300	10	G|downstream_gene_variant	41571115	Gm10_41455680_G_A*	3	96.4	1019.19	COP1-interacting protein-related

^a^ if a tagging SNP had high correspondence with more than one variant within a gene, we selected the variant with the highest average accuracy

^b^SNPs and candidate gene variants positions based on Glyma.Wm82.a2.v1 genome assembly

^c^ Average accuracies obtained by AccuTool among gene variants and significant SNPs, we only reported the accuracies >85%

^d^Distance of the candidate gene variants from the significant SNPs

^e^ Description and gene name of orthologous genes in *Arabidopsis*. ^e^Function reported for Soybean by Jia et al. 2015

* Positions of the tagging SNPs used for candidate gene selection

**Fig 5:**
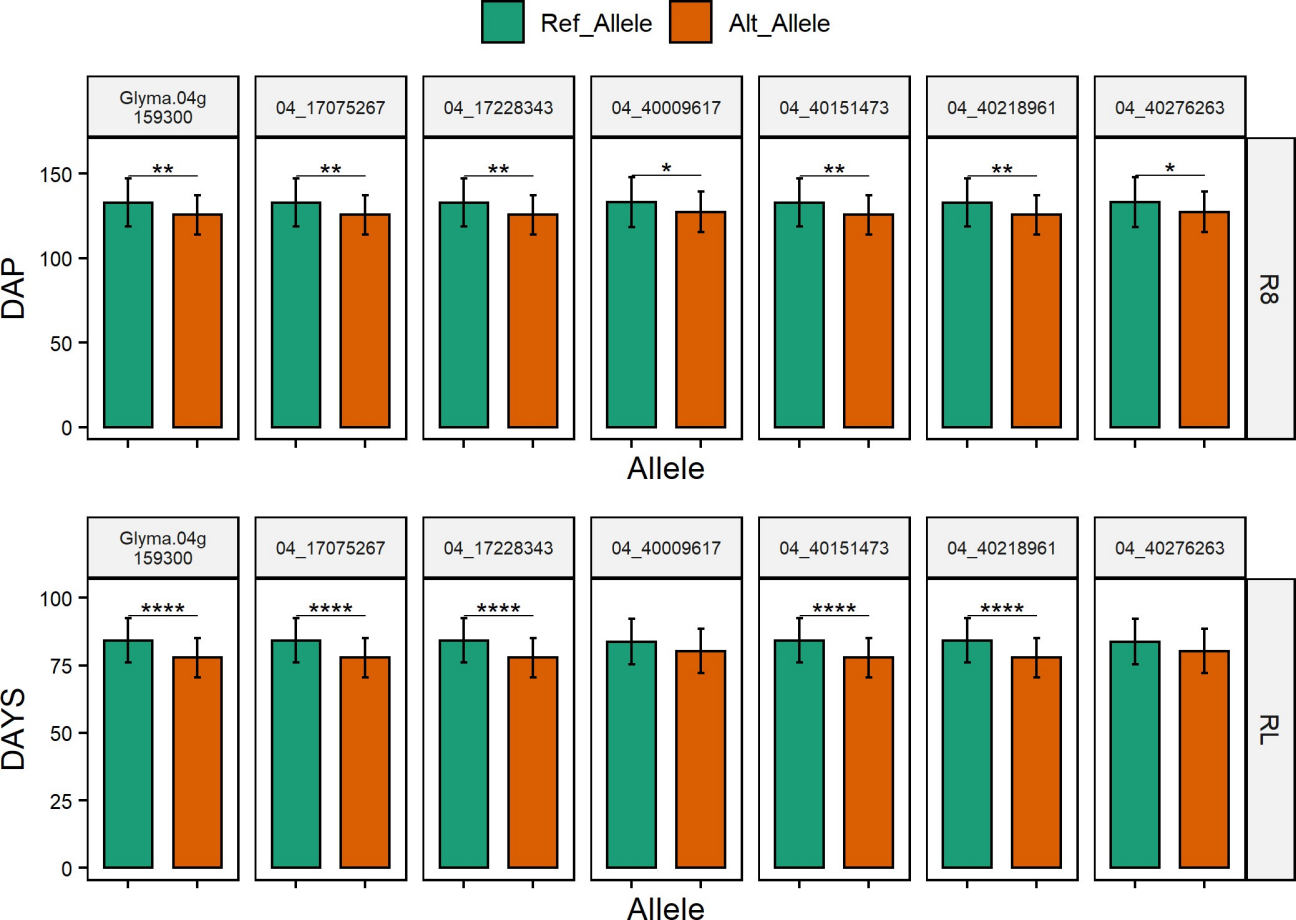
Phenotypic differences between lines carrying the reference allele (Ref_Allele) and the alternative allele (Alt_Allele) of the SNP in the *GmFulb* gene, and six tagging SNPs associated with R8, and RL. ** indicates significant differences at a p-value ≤ 0.01 between the two groups. **** indicates significant differences at a p-value ≤ 0.0001 between the two groups. DAP is days after planting.

Using the Soybean Allele Catalog Tool (https://soykb.org/SoybeanAlleleCatalogTool/), we identified 14 accessions from the studied soybean panel with sequence data available. In the 14 accessions, we inspected their allelic variation for the seven tagging SNPs, SNPs on the known *E* genes near the peaks, and allelic variants of the six candidate genes. Accessions showed allelic variation for the allelic variant (H38L) of candidate gene *GmFulb* at 04:39294836, while the allelic variants from the other five candidate genes are not reported in the Soybean Allele Catalog Tool because they are not modifying variants. Accessions also exhibited allelic variation for the six tagging SNPs from Chr 4 (Table 3). We compared the mean performance of lines carrying the reference and alternative alleles of tagging SNPs and the allelic variant of *GmFulb* that were polymorphic in the 14 accessions. There were significant differences in R8 and RL between accessions carrying the reference and alternative alleles for the allelic variant in the candidate gene *GmFulb* and the tagging SNPs from peaks on Chr 4. Two tagging SNPs from peak 2 on Chr 4 did not show significant different in RL among the 14 accessions (Fig 5). It is important to note that the set of 14 sequenced accessions did not show allelic variation for *E1la* and *E1lb* genes, the tagging SNP on Chr 10, or maturity gene *E2*. The above results support *GmFulb* as the most likely candidate for one of the peaks on Chr 4.

**Table 3:** Allelic variants of tagging SNPs, and candidate gene *GmFulb* (*Glyma*.*04159300*) for 14 accessions.

Accession	Tagging SNPs^a^						Glyma.04g159300^b^
	Gm04 40009617	Gm04 40151473	Gm04 40218961	Gm04 40276263	Gm04 17075267	Gm04 17228343	39294836
**PI515961**	C|Ref	T|Ref	G|Ref	A|Ref	G|Ref	T|Ref	T|Ref
**PI547501**	C|Ref	T|Ref	G|Ref	A|Ref	G|Ref	T|Ref	T|Ref
**PI547860**	C|Ref	T|Ref	G|Ref	A|Ref	G|Ref	T|Ref	T|Ref
**PI547885**	C|Ref	T|Ref	G|Ref	A|Ref	G|Ref	T|Ref	T|Ref
**PI548603**	C|Ref	T|Ref	G|Ref	A|Ref	G|Ref	T|Ref	T|Ref
**PI591495**	C|Ref	T|Ref	G|Ref	A|Ref	G|Ref	T|Ref	T|Ref
**PI634761**	C|Ref	T|Ref	G|Ref	A|Ref	G|Ref	T|Ref	T|Ref
**PI548543**	T|Alt	T|Ref	G|Ref	G|Alt	G|Ref	T|Ref	T|Ref
**PI548634**	T|Alt	T|Ref	G|Ref	G|Alt	G|Ref	T|Ref	T|Ref
**PI597382**	T|Alt	T|Ref	G|Ref	G|Alt	G|Ref	T|Ref	T|Ref
**PI547811**	T|Alt	C|Alt	A|Alt	G|Alt	A|Alt	C|Alt	A|H38L
**PI548177**	T|Alt	C|Alt	A|Alt	G|Alt	A|Alt	C|Alt	A|H38L
**PI548180**	T|Alt	C|Alt	A|Alt	G|Alt	A|Alt	C|Alt	A|H38L
**PI548632**	T|Alt	C|Alt	A|Alt	G|Alt	A|Alt	C|Alt	A|H38L

^a^SNP IDs and allelic information obtained from the genotype data used

in this study

^b^ allelic information obtained from the Soybean Allele Catalog Tool.

## Discussion

Despite sharing the same *e1-as*, *E2*, and *E3* alleles, wide ranges of variation and moderate to high broad sense heritabilities were observed in the studied traits, which reflects the complexity of the genetic control underlying R1, R8, and RL in soybeans. A previous study also observed wide ranges of variation, and moderate to high heritabilities for R1 and R8 in a mapping population with the same *e1-asE2E3* haplotype for major *E* genes [57]. Of the three phenotypes, R1 and RL were under a more substantial genetic control than R8 (S3 Table); however, heritability estimations depend in great part on the properties of the studied individuals and the environments with different heritabilities reported for R1, R8, and RL among previous studies [81, 82]. It is important to note that the effect of environmental factors on R1, R8, and RL is not well documented. Future studies that better understand environmental effects on *E* genes would help elucidate the mechanisms regulating flowering time and facilitating site-specific soybean breeding.

We observed higher LD decay rates compared to previous reports for accessions of the USDA soybean germplasm collection; however, this could be due to differences in the structure and diversity of the specific set of the USDA germplasm collection used in each study [41, 63, 74, 83, 84]. LD estimates are highly dependent on the sample size, and small sample sizes tend to overestimate the amount of LD [85]; therefore, our LD decay rates for euchromatic and heterochromatic regions may be inflated by the smaller sample size, lower marker density in heterochromatic regions and the way we selected the soybean panel. LD is the primary factor limiting the mapping resolution in GWAS, and overestimation of the extent of LD could lead to misinterpretations of the levels of LD in the studied populations and the sizes of identified QTLs [41, 86]; therefore, we also considered previous reports on the levels of the LD in the USDA soybean germplasm collection as a reference [63, 83].

Previous research demonstrated that maturity loci *E1*, *E2*, and *E3* are significant determinants of soybean maturity and flowering under field conditions controlling up to 66% of the variation [23, 24, 44, 45]. Here, we identified additional loci with an effect of up to two days on flowering and maturity that explain less than 30% of the observed variation, suggesting the existence of other undetected minor effect loci controlling these traits and a strong influence of the environment. From the significant SNP-trait associations, two were stable across environments, while others were environment specific. Finding stable and real associations that can be detected in various environments and plant materials is one of the major difficulties of GWAS studies [80], mainly due to the strong effect that environmental factors have on complex traits such as R1, R8, and RL. Stable marker-trait associations are helpful for marker-assisted selection (MAS), which is generally the goal of GWAS [87]. In contrast, environment-specific SNP-trait associations may play an essential role in adaptation to changing environments and maintenance of genetic variation in populations [88]; these SNPs might enhance or diminish phenotype when MAS is applied in a specific environment. The two detected peaks on Chr 4 are good candidates for validation studies, which is necessary before subsequent research in QTL cloning or marker-assisted breeding for soybean yield improvement.

Previous research showed that QTLs associated with R8 and major maturity *E* genes also affected R1 and RL mainly because they are highly correlated traits [6, 29, 57, 79]; however, in this study, pleiotropic effects existed only over R8 and RL. Our results support the idea that *E* genes can influence R8 through their influence on RL; however, there is still limited knowledge of whether *E* genes control maturity by simply influencing flowering time, reproductive length, or both [11]. In addition to the maturity loci *E1* to *E3* fixed in the studied panel of accessions, we did not detect any of the other known maturity loci. No detection of other major *E* loci could result from the lack of functional polymorphisms at these loci in the studied soybean panel or the inability to detect genetic variants on those loci due to low SNP coverage. Based on our findings, trait-specific minor effect loci could also be involved in the control of R1 and R8 in soybeans, as suggested in a previous study [41]. In addition, earlier maturing varieties sharing the same genotype for major *E* loci may be selected independently of flowering time.

One of the two stable Chr4 peaks resides about 3.2 Mb from *E1La* and 19 Mb from *E1Lb*, and the other is 14 Mb apart from *E1La* and 9 Mb from *E1Lb*, whereas the significant SNP on Chr 10 was located at 3.8 Mb apart from the *E2* gene [10, 18, 20, 22, 38, 39]. Significant Chr 4 peaks are located in heterochromatic regions, while the Chr 10 peak resides in an euchromatic region. In soybeans, LD decay rates reported for euchromatic regions are around 113 to 326 kb, while LD decay rates for heterochromatic regions range from 4 to 9 Mb [41, 63, 74, 83]. As a result, the significant SNP detected on Chr 10 does not likely correspond to the known *E2* locus. In contrast, the distance of *E1la* and *E1lb* from the two peaks on Chr 4 brings to question if the effect detected is caused by *E1la* and *E1lb*; however, there was no high correspondence between the tagging SNPs and *E1la* and *E1lb*. In addition, the absence of genetic variation for *E1la* and *E1lb* in the presence of genetic variation for the tagging SNPs suggested that detected peaks on Chr 4 in this study do not correspond to known *E* genes.

The significant peaks detected for R8 and RL on Chr 4 are located in a region similar to previously reported QTLs for R8 and RL [43, 57, 77, 78]. Researchers [57], using a bi-parental RIL population sharing the same *e1-asE2E3* haplotype for *E* genes, mapped a QTL encompassing the two Chr 4 peaks; thus, specific alleles of these major *E* genes may regulate significant peaks on Chr 4. It is important to note that GWAS using natural populations offers a higher mapping resolution than linkage-based analysis [89]. Here, we identified two association peaks 23 Mb apart from each other rather than a large single QTL as described by [57]. As opposed to [57], we did not observe an effect from Chr 4 peaks over R1; therefore, it may be possible that Chr 4 peaks are also affected by other maturity loci causing different effects on R1. In addition, a previous study found a major QTL on Chr 4 for R8 and RL that overlaps with peak-2 using a biparental RIL population, whose parents Noir (*e1-ase2e3fsE4*) and Archer (*e1-ase2E3E4*) had the same allele *e1-as* as our studied population [77]. Interestingly, they also reported *GmFulb* as the candidate gene of their QTL on Chr 4 [77], which differed in sequence between the parents of the RIL population with one of the parents harboring an amino acid variation (H38L) caused by a base substitution. This study also identified the allelic variant H38L within the *GmFulb* gene as the most likely contributor to Peak-2’s effect on R8 and RL.

The most intriguing candidate for peak-2 on Chr 4 was *GmFulb* (*Glyma*.*04g159300*), part of the *APETALA1 (AP1)/ FRUITFULL (FUL)* genes of the MADS-box transcription factor superfamily [90]. Its orthologous genes in *Arabidopsis* and other plant species play important roles in specifying floral meristem identity, reproductive transition and determining the fate of floral organ primordia [91, 92]. In soybeans, a previous study found eight *AP1/FUL*-like genes, including our candidate gene *GmFULb* (*Glyma*.*04G159300*) [90]; where the homologous gene, *GmFULa*, has shown to play roles in flowering and maturation, and to improve soybean yield by enhancing carbon assimilation [90, 93, 94]. Furthermore, *GmFULb* was one of the *E1* downregulated genes identified when overexpressing *E1* [94]; therefore, the effect of Peak-2 over R8 and RL is likely dependent on *E1* gene activity. Our results and a previous study [77] suggested that the modifying variant SNP at position 04:39294836 in *GmFulb* (H38L) is the most relevant candidate for peak-2 influencing R8 and RL.

The other candidates linked to peaks on Chr 4 were involved in seed development and maturation, floral meristem identity, photoperiod, and vernalization in soybeans, *Arabidopsis*, and other plants. *Glyma*.*04g124200*, candidate for peak-1, encodes an ABA-responsive element binding protein with a bZIP domain (AREB3) that regulates diverse developmental processes during seed development and maturation in soybean, grapes, and other legumes [95–97]. *Glyma*.*04g125700*, also a candidate of peak-1, encodes a member of the Myb family of transcription factors (MYB33). MYB transcription factors play an important role in flowering time in *Arabidopsis* by directly repressing a flowering locus (*FT*) expression in the leaf [98]. The ortholog gene in tomato, *SIMYB33*, also regulated flowering and maturity by modulating the expression of genes responsible for flowering and sugar metabolism [99]. The other candidate for peak-2, *Glyma*.*04g163100*, is a PHD finger protein that regulates expression of floral repressors related to photoperiod and vernalization pathways in *Arabidopsis*. It has been shown that mutations in the *PHD* genes cause increased expression of flowering genes and early flowering [100, 101].

The peak-3 on Chr 10 associated with R1, is located in a region that overlaps with previously reported QTLs for R1, R8, RL, seed protein, seed weight, and other disease resistance QTLs according to the SoyBase.org browser and previous studies [79, 80, 102]. We identified two potential candidate genes, *Glyma*.*10g180600* and *Glyma*.*10g182300*, located 33 and 105 kb apart from the significant SNP on Chr 10. *Glyma*.*10g180600* encodes a *Cryptochrome 2* (*CRY2a*) gene, a blue light receptor whose orthologs in tomato and *Arabidopsis* regulate leaf senescence, vegetative development, and R1. In soybeans, there are at least six cryptochrome genes in the soybean genome some of which influence flowering time. Previous research found that the *CRY2a* gene regulates leaf senescence in soybean by repressing a transcription activator (CIB1) that activates the transcription of senescence-associated genes [103–105]. Our other candidate gene, *Glyma*.*10g182300*, encodes a constitutively photomorphogenic 1 (COP1) interacting protein-related, whose ortholog in *Arabidopsis* is a central regulator of photoperiodic flowering [106, 107]. In this study, the association peak-3 showed to be highly influenced by the environment and further studies are needed to verify its role in flowering time.

In association studies, candidate genes or mechanisms underlying a trait are not directly revealed [108]; therefore, post-GWAS analysis is critical to determining relevant conclusions. A common strategy in the post-GWAS analysis is to consider the linkage disequilibrium around tagging SNPs to delimit a region of exploration for potential causal genes; however, those regions are usually large, and depending on their size, we can find hundreds or thousands of genes, which could make the identification of causal genes less accurate and more complex. To shorten the list of potential candidates, we used a methodology known as the “Synthetic phenotype association study” (SPAS), which provides a tool to measure the relationship between tagging variants from GWAS and genomic variants within nearby genes [73]. Overall, we found two stable peaks significantly associated with R8 and RL in a soybean panel sharing the same haplotype *e1-asE2E3*, where *GmFulb* (H38L) is the most significant candidate gene; however, further investigation of the specific candidate gene responsible for the phenotypic variation, as well as their underlying functional mechanisms remains to be done.

## Conclusions

This study demonstrates that characterizing populations sharing the same genotype for known *E* genes can help to identify additional loci affecting R1, R8, and RL. We identified two stable peaks that can promote or delay by two days R8 and RL, in addition to one significant SNP for R1 which was highly influenced by the environment. The detected peaks did not exhibit pleiotropic control over R1 and R8, suggesting that trait-specific minor effect loci are also involved in controlling R1 and R8, and that major *E* genes may regulate them. The independent loci acting over R1 and R8 also indicate that we could select soybean with earlier maturity independent of R1. From the total of 111 *Glyma* genes that showed high association with the three significant peaks in this study, we identified six genes that may play important roles in regulating R1, R8, and RL. Of them, *GmFulb* is the most compelling candidate given its previously reported functions. For future validation studies, parents differing for the peaks, with the most contrasting R8 phenotype, can be selected from the diversity panel to develop biparental populations. QTL flanking markers represent a valuable tool for soybean molecular breeding; however, fine mapping and map-based cloning studies of the candidate genes are necessary before they can be used effectively to breed cultivars with optimal phenological traits. In addition, further studies comparing individuals with different genotypes of major *E* genes could increase our understanding of the role of minor-effect loci in soybean flowering and maturity.

## Supporting information

S1 Fig Map showing the four locations where flowering (R1), maturity (R8), and reproductive length (RL) measurements were taken from 2017 to 2020. Orange squares delimit MG III and IV adaptation regions.
(TIF)

S2 Fig Heatmap from a genomic relationship matrix of the three hundred twenty-nine *G*. max USDA accession used in this study. (TIF)

S3 Fig Average squared correlation of allele frequencies (*r*^*2*^) against distance across the whole genome from heterochromatic (red) and euchromatic regions (blue). (TIF)

S4 Fig Histograms of the frequency distribution of flowering (R1), maturity (R8), and reproductive length (RL) for three hundred twenty-nine *G*. *max* accessions. Data are measurements of R1 and R8 in days after planting (DAP), and RL as the number of days between R1 and R8 from four years and four locations.
(TIF)

S5 Fig Optimal number of clusters by k-means using the average silhouette width. (TIF)

S6 Fig SNP density plot across 20 chromosomes of soybean representing the number of SNPs within 1 Mb window size. (TIF)

S7 Fig Significant SNPs from genome-wide association analysis by individual environments, years, and across all environments. R1 is flowering time, R8 is maturity time, and RL is reproductive length. The plot is color coded by the effect of the SNPs for each trait. SNP positions are based on the Wm82.a2.v1 genome assembly.
(TIF)

S8 Fig Phenotypic differences between lines carrying the reference allele (Ref_Allele) and the alternative allele (Alt_Allele) of the seven tagging SNPs associated with R1, R8, and RL. Bar plots show the differences in R8 (A), RL (B), and R1 (C). **** indicates significant differences at a *p*-value ≤ 0.0001 between the two groups. DAP is days after planting.
(TIF)

S1 Table Planting dates of experimental environments. (PDF)

S2 Table Mean flowering (R1), and maturity (R8) time in days after planting (DAP) and mean reproductive length (RL) in number of days per accession across all environments. (PDF)

S3 Table Descriptive statistics of phenotypic variation, genotypic variance (G) and broad-sense heritability (H^2^) of days to flowering (R1), days to maturity (R8), and reproductive length for 329 *G*. max USDA accessions evaluated at nine environments. (PDF)

S4 Table Percentage of SNPs by heterochromatic and euchromatic regions of each chromosome. (PDF)

S5 Table Linkage Disequilibrium (LD) decay rate across 20 chromosomes within euchromatic and heterochromatic regions. (PDF)

S6 Table Summary of the single-nucleotide polymorphisms (SNPs) significantly associated with flowering time (R1), maturity time (R8), and reproductive length (RL) in three hundred twenty-nine G. max accessions across environments and years. (PDF)

S7 Table Summary of the significant single-nucleotide polymorphisms (SNPs) associated with flowering time (R1), maturity time (R8), and reproductive length (RL) in three hundred twenty-nine G. max accessions by individual environments, years, and across all environments and years. (PDF)

S8 Table List of potential candidate genes by tagging SNPs with average accuracy and descriptions for flowering time (R1), maturity time (R8), and reproductive length (RL). (PDF)

S9 Table Correspondence between tagging SNPs and known E genes measured by average accuracy obtained from AcuTool. (PDF)

S1 File (DOCX)

## References

[1] Liu K. Soybeans: Chemistry, Technology, and Utilization. In: Liu K, editor. New York: Chapman and Hall; 1997. p. 532.

[2] Hymowitz T, Collins FI. Variability of Sugar Content in Seed of. Agron J. 1974;66:239–40.

[3] Burton JW. Soyabean (Glycine max (L.) Merr.). Field Crops Res. 1997;53(1–3):171–86.

[4] Bu T, Lu S, Wang K, Dong L, Li S, Xie Q, et al. A critical role of the soybean evening complex in the control of photoperiod sensitivity and adaptation. Proc Natl Acad Sci U S A. 2021 Feb;118(8). doi: 10.1073/pnas.2010241118 33558416 PMC7923351

[5] Garner W. W. and Allard H. A. Effect of the relative length of day and night and other factors of the environment on growth and reproduction plants. J Agric Res. 1920;48(7):415.

[6] Watanabe S, Harada K, Abe J. Genetic and molecular bases of photoperiod responses of flowering in soybean. Vol. 61, Breeding Science. Breed Sci; 2012. p. 531–43. doi: 10.1270/jsbbs.61.531 23136492 PMC3406791

[7] Destro D, Carpentieri-Pípolo V, Kiihl RAS, Almeida LA. Photoperiodism and Genetic Control of the Long Juvenile Period in Soybean: A Review. Crop Breeding and Applied Biotechnology. 2001 Mar 31;1(1):72–92.

[8] Miladinović J, Ćeran M, Đorđević V, Balešević-Tubić S, Petrović K, Đukić V, et al. Allelic variation and distribution of the major maturity genes in different soybean collections. Front Plant Sci. 2018 Sep 4;9. doi: 10.3389/fpls.2018.01286 30233624 PMC6131654

[9] Roger Boerma H, Specht JE. Soybeans: Improvement, production, and uses. Soybeans: Improvement, Production, and Uses. wiley; 2016. 1–1119 p.

[10] Harada K, Watanabe S, Zhengjun X, Tsubokura Y, Yamanaka N, Anai T. Positional Cloning of the Responsible Genes for Maturity Loci E1, E2 and E3 in Soybean. In: Soybean—Genetics and Novel Techniques for Yield Enhancement. 2011.

[11] Kumudini S V., Pallikonda PK, Steele C. Photoperiod and E-genes influence the duration of the reproductive phase in soybean. Crop Sci. 2007;47(4):1510–7.

[12] Cober ER, Curtis DF, Stewart DW, Morrison MJ. Quantifying the effects of photoperiod, temperature and daily irradiance on flowering time of soybean isolines. Plants. 2014;3(4):476–97. doi: 10.3390/plants3040476 27135515 PMC4844283

[13] Kong F, Nan H, Cao D, Li Y, Wu F, Wang J, et al. A new dominant gene E9 conditions early flowering and maturity in soybean. Crop Sci. 2014 Nov 1;54(6):2529–35.

[14] Bonato ER, Vello NA. E6, a dominant gene conditioning early flowering and maturity in soybeans. Genet Mol Biol. 1999;22(2):229–32.

[15] Zhang D, Wang X, Li S, Wang C, Gosney MJ, Mickelbart M V., et al. A Post-domestication Mutation, Dt2, Triggers Systemic Modification of Divergent and Convergent Pathways Modulating Multiple Agronomic Traits in Soybean. Mol Plant. 2019 Oct;12(10):1366–82. doi: 10.1016/j.molp.2019.05.010 31152912

[16] Ping J, Liu Y, Sun L, Zhao M, Li Y, She M, et al. Dt2 is a gain-of-function MADS-domain factor gene that specifies semideterminacy in soybean. Plant Cell. 2014 Aug 26;26(7):2831–42. doi: 10.1105/tpc.114.126938 25005919 PMC4145117

[17] Buzzell RI. Inheritance of a soybean flowering response to fluorescent-daylength conditions. Canadian Journal of Genetics and Cytology. 1971;13(4):703–7.

[18] Bernard RL. Two Major Genes for Time of Flowering and Maturity in Soybeans 1. Crop Sci. 1971 Mar 1;11(2):242–4.

[19] Takeshima R, Nan H, Harigai K, Dong L, Zhu J, Lu S, et al. Functional divergence between soybean FLOWERING LOCUS T orthologues FT2a and FT5a in post-flowering stem growth. J Exp Bot. 2019 Aug;70(15):3941–53. doi: 10.1093/jxb/erz199 31035293 PMC6685666

[20] Watanabe S, Xia Z, Hideshima R, Tsubokura Y, Sato S, Yamanaka N, et al. A map-based cloning strategy employing a residual heterozygous line reveals that the GIGANTEA gene is involved in soybean maturity and flowering. Genetics. 2011 Jun 1;188(2):395–407. doi: 10.1534/genetics.110.125062 21406680 PMC3122305

[21] Zhu J, Takeshima R, Harigai K, Xu M, Kong F, Liu B, et al. Loss of function of the E1-like-B gene associates with early flowering under long-day conditions in Soybean. Front Plant Sci. 2019 Jan 8;9. doi: 10.3389/fpls.2018.01867 30671065 PMC6331540

[22] Xu M, Yamagishi N, Zhao C, Takeshima R, Kasai M, Watanabe S, et al. The soybean-specific maturity gene E1 family of floral repressors controls night-break responses through down-regulation of FLOWERING LOCUS T orthologs. Plant Physiol. 2015;168(4):1735–46. doi: 10.1104/pp.15.00763 26134161 PMC4528769

[23] Xia Z, Watanabe S, Yamada T, Tsubokura Y, Nakashima H, Zhai H, et al. Positional cloning and characterization reveal the molecular basis for soybean maturity locus E1 that regulates photoperiodic flowering. Proc Natl Acad Sci U S A. 2012 Aug 7;109(32):E2155–64. doi: 10.1073/pnas.1117982109 22619331 PMC3420212

[24] Tsubokura Y, Watanabe S, Xia Z, Kanamori H, Yamagata H, Kaga A, et al. Natural variation in the genes responsible for maturity loci E1, E2, E3 and E4 in soybean. Ann Bot. 2014;113(3):429–41. doi: 10.1093/aob/mct269 24284817 PMC3906962

[25] Zhai H, Lü S, Wu H, Zhang Y, Zhang X, Yang J, et al. Diurnal Expression Pattern, Allelic Variation, and Association Analysis Reveal Functional Features of the E1 Gene in Control of Photoperiodic Flowering in Soybean. PLoS One. 2015 Aug 14;10(8):e0135909 doi: 10.1371/journal.pone.0135909 26275311 PMC4537287

[26] Watanabe S, Hideshima R, Zhengjun X, Tsubokura Y, Sato S, Nakamoto Y, et al. Map-based cloning of the gene associated with the soybean maturity locus E3. Genetics. 2009 Aug;182(4):1251–62. doi: 10.1534/genetics.108.098772 19474204 PMC2728863

[27] Tsubokura Y, Matsumura H, Xu M, Liu B, Nakashima H, Anai T, et al. Genetic variation in soybean at the maturity locus e4 is involved in adaptation to long days at high latitudes. Agronomy. 2013 Feb;3(1):117–34.

[28] Liu B, Kanazawa A, Matsumura H, Takahashi R, Harada K, Abe J. Genetic redundancy in soybean photoresponses associated with duplication of the phytochrome A gene. Genetics. 2008 Oct 1;180(2):995–1007. doi: 10.1534/genetics.108.092742 18780733 PMC2567397

[29] Xu M, Xu Z, Liu B, Kong F, Tsubokura Y, Watanabe S, et al. Genetic variation in four maturity genes affects photoperiod insensitivity and PHYA-regulated post-flowering responses of soybean. BMC Plant Biol. 2013 Jun 25;13(1):1–14. doi: 10.1186/1471-2229-13-91 23799885 PMC3698206

[30] Dissanayaka A, Rodriguez TO, Di S, Yan F, Githiri SM, Rodas FR, et al. Quantitative trait locus mapping of soybean maturity gene E5. Breed Sci. 2016 Jun;66(3):407–15. doi: 10.1270/jsbbs.15160 27436951 PMC4902463

[31] Mcblain BA, Bernard RL. A new gene affecting the time of flowering and maturity in soybeans. Journal of Heredity. 1987 May;78(3):160–2.

[32] Wei C, Yang H, Wang S, Zhao J, Liu C, Gao L, et al. Draft genome sequence of *Camellia sinensis* var. *sinensis* provides insights into the evolution of the tea genome and tea quality. Proceedings of the National Academy of Sciences. 2018;201719622. 10.1073/pnas.1719622115 PMC5939082 29678829

[33] Fang C, Liu J, Zhang T, Su T, Li S, Cheng Q, et al. A recent retrotransposon insertion of J caused E6 locus facilitating soybean adaptation into low latitude. J Integr Plant Biol. 2021;63(6):995–1003. doi: 10.1111/jipb.13034 33205888

[34] Nissan N, Cober ER, Sadowski M, Charette M, Golshani A, Samanfar B. Identifying new variation at the J locus, previously identified as e6, in long juvenile ‘Paranagoiana’ soybean. Theoretical and Applied Genetics. 2021 Apr;134(4):1007–14. doi: 10.1007/s00122-020-03746-2 33386860 PMC7973924

[35] Molnar SJ, Rai S, Charette M, Cober ER. Simple sequence repeat (SSR) markers linked to E1, E3, E4, and E7 maturity genes in soybean. Genome. 2003;46(6):1024–36. doi: 10.1139/g03-079 14663521

[36] Dietz N, Combs-Giroir R, Cooper G, Stacey M, Miranda C, Bilyeu K. Geographic distribution of the E1 family of genes and their effects on reproductive timing in soybean. BMC Plant Biol. 2021;21(1):1–14. 34587901 10.1186/s12870-021-03197-x PMC8480027

[37] Cober ER, Voldeng HD. A new soybean maturity and photoperiod-sensitivity locus linked to E1 and T. Crop Sci. 2001 May 1;41(3):698–701.

[38] Cober ER, Molnar SJ, Charette M, Voldeng HD. A new locus for early maturity in soybean. Crop Sci. 2010 Mar;50(2):524–7.

[39] Zhao C, Takeshima R, Zhu J, Xu M, Sato M, Watanabe S, et al. A recessive allele for delayed flowering at the soybean maturity locus E9 is a leaky allele of FT2a, a FLOWERING LOCUS T ortholog. BMC Plant Biol. 2016 Jan 19;16(1):1–15. doi: 10.1186/s12870-016-0704-9 26786479 PMC4719747

[40] Wang F, Nan H, Chen L, Fang C, Zhang H, Su T, et al. A new dominant locus, E11, controls early flowering time and maturity in soybean. Molecular Breeding. 2019 May;39(5).

[41] Zhang J, Song Q, Cregan PB, Nelson RL, Wang X, Wu J, et al. Genome-wide association study for flowering time, maturity dates and plant height in early maturing soybean (Glycine max) germplasm. BMC Genomics. 2015;16(1). doi: 10.1186/s12864-015-1441-4 25887991 PMC4449526

[42] Fang C, Ma Y, Wu S, Liu Z, Wang Z, Yang R, et al. Genome-wide association studies dissect the genetic networks underlying agronomical traits in soybean. Genome Biol. 2017 Dec 24;18(1):161. doi: 10.1186/s13059-017-1289-9 28838319 PMC5571659

[43] Cheng L, Wang Y, Zhang C, Wu C, Xu J, Zhu H, et al. Genetic analysis and QTL detection of reproductive period and post-flowering photoperiod responses in soybean. Theoretical and Applied Genetics. 2011 Aug 1;123(3):421–9. doi: 10.1007/s00122-011-1594-8 21556700

[44] Jiang B, Nan H, Gao Y, Tang L, Yue Y, Lu S, et al. Allelic combinations of soybean maturity loci E1, E2, E3 and E4 result in diversity of maturity and adaptation to different latitudes. PLoS One. 2014;9(8). doi: 10.1371/journal.pone.0106042 25162675 PMC4146597

[45] Liu L, Song W, Wang L, Sun X, Qi Y, Wu T, et al. Allele combinations of maturity genes E1-E4 affect adaptation of soybean to diverse geographic regions and farming systems in China. PLoS One. 2020;15(7). doi: 10.1371/journal.pone.0235397 32628713 PMC7337298

[46] Zhai H, Lü S, Liang S, Wu H, Zhang X, Liu B, et al. GmFT4, a homolog of FLOWERING LOCUS T, is positively regulated by E1 and functions as a flowering repressor in soybean. PLoS One. 2014;9(2). 10.1371/journal.pone.0089030 PMC3929636 24586488

[47] Langewisch T, Zhang H, Vincent R, Joshi T, Xu D, Bilyeu K. Major soybean maturity gene haplotypes revealed by SNPViz analysis of 72 sequenced soybean genomes. Zhang T, editor. PLoS One. 2014 Apr 11;9(4):e94150. doi: 10.1371/journal.pone.0094150 24727730 PMC3984090

[48] Wolfgang G, An Y qiang C. Genetic separation of southern and northern soybean breeding programs in North America and their associated allelic variation at four maturity loci. Molecular Breeding. 2017;37(1). doi: 10.1007/s11032-016-0611-7 28127254 PMC5226990

[49] Zimmer G, Miller MJ, Steketee CJ, Jackson SA, de Tunes LVM, Li Z. Genetic control and allele variation among soybean maturity groups 000 through IX. Plant Genome. 2021 Nov 1;14(3):e20146. doi: 10.1002/tpg2.20146 34514734 PMC12807432

[50] Yang J, Benyamin B, McEvoy BP, Gordon S, Henders AK, Nyholt DR, et al. Common SNPs explain a large proportion of the heritability for human height. Nat Genet. 2010 Jun;42(7):565–9. doi: 10.1038/ng.608 20562875 PMC3232052

[51] Huang W, Richards S, Carbone MA, Zhu D, Anholt RRH, Ayroles JF, et al. Epistasis dominates the genetic architecture of Drosophila quantitative traits. Proc Natl Acad Sci U S A. 2012 Sep;109(39):15553–9. doi: 10.1073/pnas.1213423109 22949659 PMC3465439

[52] Manolio TA, Collins FS, Cox NJ, Goldstein DB, Hindorff LA, Hunter DJ, et al. Finding the missing heritability of complex diseases. Vol. 461, Nature. Nature; 2009. p. 747–53. doi: 10.1038/nature08494 19812666 PMC2831613

[53] Phillips PC. Epistasis—The essential role of gene interactions in the structure and evolution of genetic systems. Vol. 9, Nature Reviews Genetics. Nature Publishing Group; 2008. p. 855–67. 10.1038/nrg2452 PMC2689140 18852697

[54] Ahsan A, Monir M, Meng X, Rahaman M, Chen H, Chen M. Identification of epistasis loci underlying rice flowering time by controlling population stratification and polygenic effect. DNA Research. 2019;26(2):119–30. doi: 10.1093/dnares/dsy043 30590457 PMC6476725

[55] Doust AN, Lukens L, Olsen KM, Mauro-Herrera M, Meyer A, Rogers K. Beyond the single gene: How epistasis and gene-byenvironment effects influence crop domestication. Proc Natl Acad Sci U S A. 2014 Apr;111(17):6178–83. doi: 10.1073/pnas.1308940110 24753598 PMC4035984

[56] Kim KH, Kim JY, Lim WJ, Jeong S, Lee HY, Cho Y, et al. Genome-wide association and epistatic interactions of flowering time in soybean cultivar. PLoS One. 2020 Jan;15(1):e0228114. doi: 10.1371/journal.pone.0228114 31968016 PMC6975553

[57] Kong L, Lu S, Wang Y, Fang C, Wang F, Nan H, et al. Quantitative trait locus mapping of flowering time and maturity in soybean using next-generation sequencing-based analysis. Front Plant Sci. 2018;9:995. doi: 10.3389/fpls.2018.00995 30050550 PMC6050445

[58] Langewisch T, Lenis J, Jiang GL, Wang D, Pantalone V, Bilyeu K. The development and use of a molecular model for soybean maturity groups. BMC Plant Biol. 2017 May 30;17(1). doi: 10.1186/s12870-017-1040-4 28558691 PMC5450301

[59] Miranda C, Culp C, Škrabišová M, Joshi T, Belzile F, Grant DM, et al. Molecular tools for detecting Pdh1 can improve soybean breeding efficiency by reducing yield losses due to pod shatter. Molecular Breeding. 2019;39(2).

[60] Wickham Hadley. ggplot2: Elegant Graphics for Data Analysis. Springer-Verlag New York; 2016. Available from: https://ggplot2.tidyverse.org

[61] Fehr WP, Caviness CE. Stages of soybean development. Agriculture and Home Economics Experiment Station and Cooperative Extension Service. 1977. 11 p.

[62] Song Q, Hyten DL, Jia G, Quigley C V, Fickus EW, Nelson RL, et al. Development and Evaluation of SoySNP50K, a High-Density Genotyping Array for Soybean. PLoS One. 2013;8(1):54985. doi: 10.1371/journal.pone.0054985 23372807 PMC3555945

[63] Xavier A, Thapa R, Muir WM, Rainey KM. Population and quantitative genomic properties of the USDA soybean germplasm collection. Plant Genetic Resources: Characterisation and Utilisation. 2018 Dec 1;16(6):513–23.

[64] Grant D, Nelson RT, Cannon SB, Shoemaker RC. SoyBase, the USDA-ARS soybean genetics and genomics database. Nucleic Acids Res. 2009 Jan;38(SUPPL.1):D843–6. doi: 10.1093/nar/gkp798 20008513 PMC2808871

[65] Xavier A, Xu S, Muir WM, Rainey KM. NAM: Association studies in multiple populations. Bioinformatics. 2015 Aug 4;31(23):3862–4. doi: 10.1093/bioinformatics/btv448 26243017

[66] Slatkin M, Excoffier L. Testing for linkage disequilibrium in genotypic data using the Expectation-Maximization algorithm. Heredity (Edinb). 1996;76(4):377–83. doi: 10.1038/hdy.1996.55 8626222

[67] Song Q, Jenkins J, Jia G, Hyten DL, Pantalone V, Jackson SA, et al. Construction of high resolution genetic linkage maps to improve the soybean genome sequence assembly Glyma1.01. BMC Genomics. 2016;17(1). doi: 10.1186/s12864-015-2344-0 26739042 PMC4704267

[68] Huang X, Wei X, Sang T, Zhao Q, Feng Q, Zhao Y, et al. Genome-wide asociation studies of 14 agronomic traits in rice landraces. Nat Genet. 2010 Oct 24;42(11):961–7. 20972439 10.1038/ng.695

[69] Bates D, Mächler M, Bolker BM, Walker SC. Fitting linear mixed-effects models using lme4. J Stat Softw. 2015 Oct 7;67(1):1–48.

[70] Lado B, Matus I, Rodríguez A, Inostroza L, Poland J, Belzile F, et al. Increased genomic prediction accuracy in wheat breeding through spatial adjustment of field trial data. G3: Genes, Genomes, Genetics. 2013;3(12):2105–14. doi: 10.1534/g3.113.007807 24082033 PMC3852373

[71] Gao X, Starmer J, Martin ER. A multiple testing correction method for genetic association studies using correlated single nucleotide polymorphisms. Genet Epidemiol. 2008 May;32(4):361–9. doi: 10.1002/gepi.20310 18271029

[72] LiLin Y. Package “CMplot.” 2008.

[73] Škrabišová M, Dietz N, Zeng S, On Chan Y, Wang J, Liu Y, et al. A novel Synthetic phenotype association study approach reveals the landscape of association for genomic variants and phenotypes. J Adv Res. 2022 Apr. doi: 10.1016/j.jare.2022.04.004 36513408 PMC9788956

[74] Hwang EY, Song Q, Jia G, Specht JE, Hyten DL, Costa J, et al. A genome-wide association study of seed protein and oil content in soybean. BMC Genomics. 2014;15(1):1–12. doi: 10.1186/1471-2164-15-1 24382143 PMC3890527

[75] Xavier A, Rainey KM. Quantitative genomic dissection of soybean yield components. G3: Genes, Genomes, Genetics. 2020 Feb 1;10(2):665–75. doi: 10.1534/g3.119.400896 31818873 PMC7003100

[76] Hyten DL, Choi IY, Song Q, Shoemaker RC, Nelson RL, Costa JM, et al. Highly variable patterns of linkage disequilibrium in multiple soybean populations. Genetics. 2007 Apr;175(4):1937–44. doi: 10.1534/genetics.106.069740 17287533 PMC1855121

[77] Wang L, Fang C, Liu J, Zhang T, Kou K, Su T, et al. Identification of major QTLs for flowering and maturity in soybean by genotyping-by-sequencing analysis. Molecular Breeding. 2020;40(10).

[78] Zhang WK, Wang YJ, Luo GZ, Zhang JS, He CY, Wu XL, et al. QTL mapping of ten agronomic traits on the soybean (Glycine max L. Merr.) genetic map and their association with EST markers. Theoretical and Applied Genetics. 2004;108(6):1131–9. doi: 10.1007/s00122-003-1527-2 15067400

[79] Copley TR, Duceppe MO, O’Donoughue LS. Identification of novel loci associated with maturity and yield traits in early maturity soybean plant introduction lines. BMC Genomics. 2018 Mar;19(1):1–12. 29490606 10.1186/s12864-018-4558-4 PMC5831853

[80] Zuo Q, Hou J, Zhou B, Wen Z, Zhang S, Gai J, et al. Identification of qtls for growth period traits in soybean using association analysis and linkage mapping. Plant Breeding. 2013;132(3):317–23.

[81] Mao T, Li J, Wen Z, Wu T, Wu C, Sun S, et al. Association mapping of loci controlling genetic and environmental interaction of soybean flowering time under various photo-thermal conditions. BMC Genomics. 2017 May;18(1):1–17. 28549456 10.1186/s12864-017-3778-3 PMC5446728

[82] Ghodrati G. Study of genetic variation and broad sense heritability for some qualitative and quantitative traits in soybean (Glycine max L.) genotypes. Vol. 2, Current Opinion in Agriculture Curr. Opin. Agric. 2013.

[83] Wen Z, Boyse JF, Song Q, Cregan PB, Wang D. Genomic consequences of selection and genome-wide association mapping in soybean. BMC Genomics. 2015;16(1). doi: 10.1186/s12864-015-1872-y 26334313 PMC4559069

[84] Hwang S, Ray JD, Cregan PB, King C Andy, Davies MK, Purcell LC, et al. Genetics and mapping of quantitative traits for nodule number, weight, and size in soybean (Glycine max L.[Merr.]). Euphytica. 2014;195:419–34.

[85] Tenesa A, Wright AF, Knott SA, Carothers AD, Hayward C, Angius A, et al. Extent of linkage disequilibrium in a Sardinian sub-isolate: sampling and methodological considerations. Hum Mol Genet. 2003;13(1):25–33. doi: 10.1093/hmg/ddh001 14613964

[86] Long Q, Rabanal FA, Meng D, Huber CD, Farlow A, Platzer A, et al. Massive genomic variation and strong selection in Arabidopsis thaliana lines from Sweden. Nat Genet. 2013 Jun;45(8):884–90. doi: 10.1038/ng.2678 23793030 PMC3755268

[87] Liu R, Fang L, Yang T, Zhang X, Hu J, Zhang H, et al. Marker-trait association analysis of frost tolerance of 672 worldwide pea (Pisum sativum L.) collections. Sci Rep. 2017 Jul;7(1):1–10. 28724947 10.1038/s41598-017-06222-y PMC5517424

[88] Wang X, Pang Y, Zhang J, Zhang Q, Tao Y, Feng B, et al. Genetic background effects on QTL and QTL × environment interaction for yield and its component traits as revealed by reciprocal introgression lines in rice. Crop Journal. 2014 Dec;2(6):345–57.

[89] Wang T, Wei L, Wang J, Xie L, Li YY, Ran S, et al. Integrating GWAS, linkage mapping and gene expression analyses reveals the genetic control of growth period traits in rapeseed (Brassica napus L.). Biotechnol Biofuels. 2020 Aug;13(1):1–19. doi: 10.1186/s13068-020-01774-0 32774455 PMC7397576

[90] Jia Z, Jiang B, Gao X, Yue Y, Fei Z, Sun H, et al. GmFULa, a FRUITFULL homolog, functions in the flowering and maturation of soybean. Plant Cell Rep. 2015;34(1):121–32. doi: 10.1007/s00299-014-1693-5 25326369

[91] Mandel MA, Yanofsky MF. The arabidopsis AGL8 MADS box gene is expressed in inflorescence meristems and is negatively regulated by APETALA1. Plant Cell. 1995;7(11):1763–71. doi: 10.1105/tpc.7.11.1763 8535133 PMC161036

[92] Zhang L, Zhang H, Qiao L, Miao L, Yan D, Liu P, et al. Wheat MADS-box gene TaSEP3-D1 negatively regulates heading date. Crop Journal. 2021 Oct;9(5):1115–23.

[93] Yue Y, Sun S, Li J, Yu H, Wu H, Sun B, et al. GmFULa improves soybean yield by enhancing carbon assimilation without altering flowering time or maturity. Plant Cell Rep. 2021;40(10):1875–88. doi: 10.1007/s00299-021-02752-y 34272585 PMC8494661

[94] Zhai H, Wan Z, Jiao S, Zhou J, Xu K, Nan H, et al. GmMDE genes bridge the maturity gene E1 and florigens in photoperiodic regulation of flowering in soybean. Plant Physiol. 2022 Jun;189(2):1021–36. doi: 10.1093/plphys/kiac092 35234946 PMC9157081

[95] Nicolas P, Lecourieux D, Kappel C, Cluzet S, Cramer G, Delrot S, et al. The basic leucine zipper transcription factor ABSCISIC ACID RESPONSE ELEMENT-BINDING FACTOR2 is an important transcriptional regulator of abscisic acid-dependent grape berry ripening processes. Plant Physiol. 2014 Jan;164(1):365–83. doi: 10.1104/pp.113.231977 24276949 PMC3875815

[96] Zinsmeister J, Lalanne D, Terrasson E, Chatelain E, Vandecasteele C, Ly Vu B, et al. ABI5 is a regulator of seed maturation and longevity in legumes. Plant Cell. 2016 Nov;28(11):2735–54. doi: 10.1105/tpc.16.00470 27956585 PMC5155344

[97] Jo L, Pelletier JM, Hsu SW, Baden R, Goldberg RB, Harada JJ. Combinatorial interactions of the LEC1 transcription factor specify diverse developmental programs during soybean seed development. Proc Natl Acad Sci U S A. 2020 Jan;117(2):1223–32. doi: 10.1073/pnas.1918441117 31892538 PMC6969526

[98] Yan Y, Shen L, Chen Y, Bao S, Thong Z, Yu H. A MYB-Domain Protein EFM Mediates Flowering Responses to Environmental Cues in Arabidopsis. Dev Cell. 2014 Aug;30(4):437–48. doi: 10.1016/j.devcel.2014.07.004 25132385

[99] Zhang Y, Zhang B, Yang T, Zhang J, Liu B, Zhan X, et al. The GAMYB-like gene SlMYB33 mediates flowering and pollen development in tomato. Hortic Res. 2020;7:133. doi: 10.1038/s41438-020-00366-1 32922805 PMC7459326

[100] Qian F, Zhao QY, Zhang TN, Li YL, Su YN, Li L, et al. A histone H3K27me3 reader cooperates with a family of PHD finger-containing proteins to regulate flowering time in Arabidopsis. J Integr Plant Biol. 2021 Apr;63(4):787–802. doi: 10.1111/jipb.13067 33433058

[101] Sung S, Schmitz RJ, Amasino RM. A PHD finger protein involved in both the vernalization and photoperiod pathways in Arabidopsis. Genes Dev. 2006 Dec;20(23):3244–8. doi: 10.1101/gad.1493306 17114575 PMC1686601

[102] Su T, Wang Y, Li S, Wang L, Kou K, Kong L, et al. A flowering time locus dependent on E2 in soybean. Molecular Breeding. 2021 Jun;41(6):1–14. doi: 10.1007/s11032-021-01224-1 37309325 PMC10236059

[103] Meng Y, Li H, Wang Q, Liu B, Lin C. Blue light-dependent interaction between cryptochrome2 and CIB1 regulates transcription and leaf senescence in soybean. Plant Cell. 2013;25(11):4405–20. Available from: www.plantcell.org/cgi/doi/10.1105/tpc.113.116590 24272488 10.1105/tpc.113.116590 PMC3875726

[104] Zhang Q, Li H, Li R, Hu R, Fan C, Chen F, et al. Association of the circadian rhythmic expression of GmCRY1a with a latitudinal cline in photoperiodic flowering of soybean. Proc Natl Acad Sci U S A. 2008 Dec;105(52):21028–33. doi: 10.1073/pnas.0810585105 19106300 PMC2607247

[105] Giliberto L, Perrotta G, Pallara P, Weller JL, Fraser PD, Bramley PM, et al. Manipulation of the Blue Light Photoreceptor Cryptochrome 2 in Tomato Affects Vegetative Development, Flowering Time, and Fruit Antioxidant Content. Plant Physiol. 2005 Jan 1. doi: 10.1104/pp.104.051987 15618424 PMC548851

[106] Deng XW, Xu D, Zhu D. The role of COP1 in repression of photoperiodic flowering. Vol. 5, F1000Research. Faculty of 1000 Ltd; 2016. 10.12688/f1000research.7346.1 PMC4756798 26949521

[107] Holm M, Ma LG, Qu LJ, Deng XW. Two interacting bZIP proteins are direct targets of COP1-mediated control of light-dependent gene expression in Arabidopsis. Genes Dev. 2002 May 15;16(10):1247–59. doi: 10.1101/gad.969702 12023303 PMC186273

[108] Visscher PM, Wray NR, Zhang Q, Sklar P, McCarthy MI, Brown MA, et al. 10 Years of GWAS Discovery: Biology, Function, and Translation. Vol. 101, American Journal of Human Genetics. Elsevier; 2017. p. 5–22. 28686856 10.1016/j.ajhg.2017.06.005 PMC5501872

