# Supplementary figures and images for: Genome-wide association study reveals GmFulb as candidate gene for maturity time and reproductive length in soybeans (Glycine max)
Source: PLoS One. 2024 Jan 19;19(1):e0294123. doi: 10.1371/journal.pone.0294123 (PMC10798547; doi:10.1371/journal.pone.0294123)

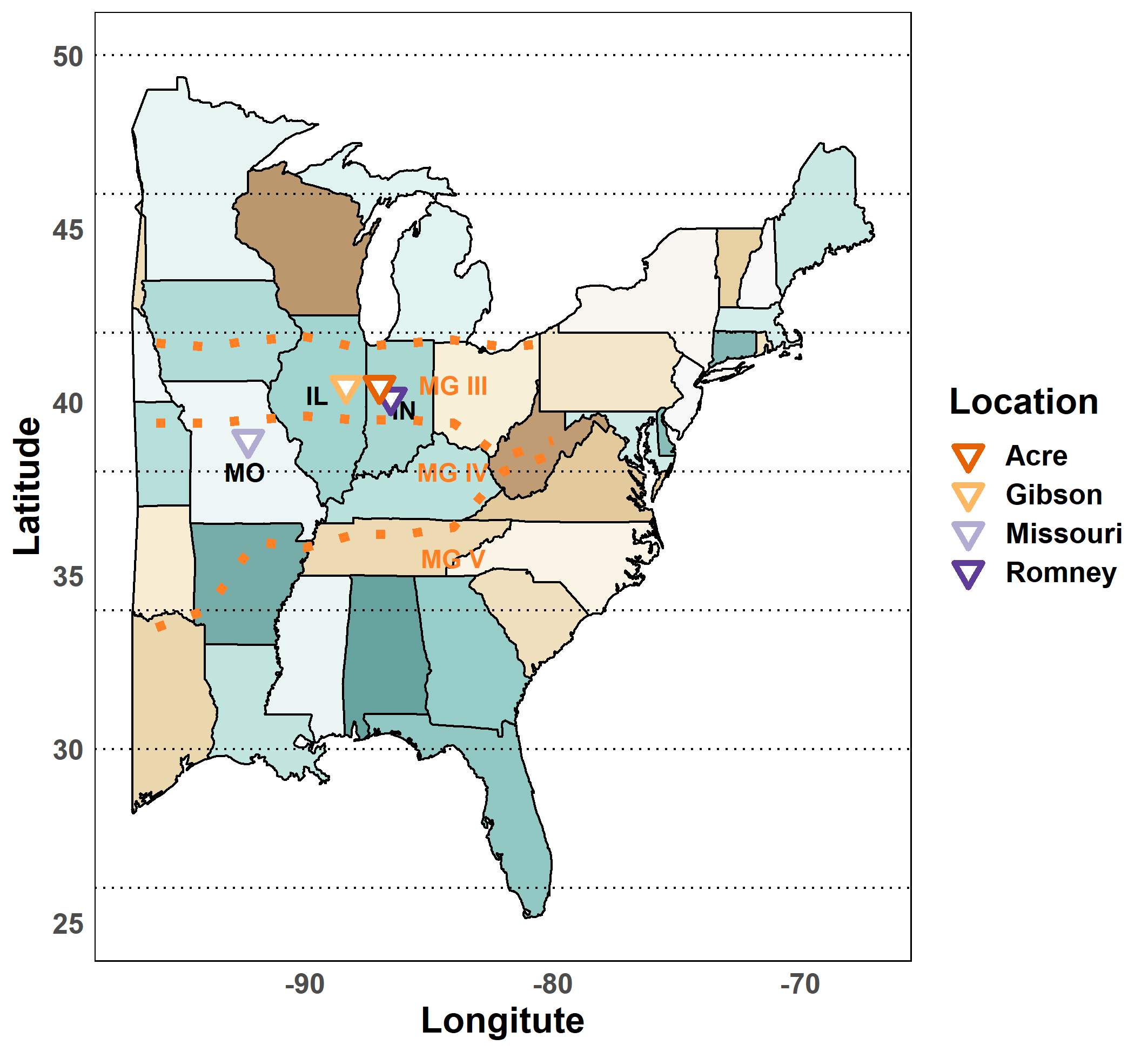

Supplement: S1 Fig — Orange squares delimit MG III and IV adaptation regions. (TIF) [file pone.0294123.s001.tif]

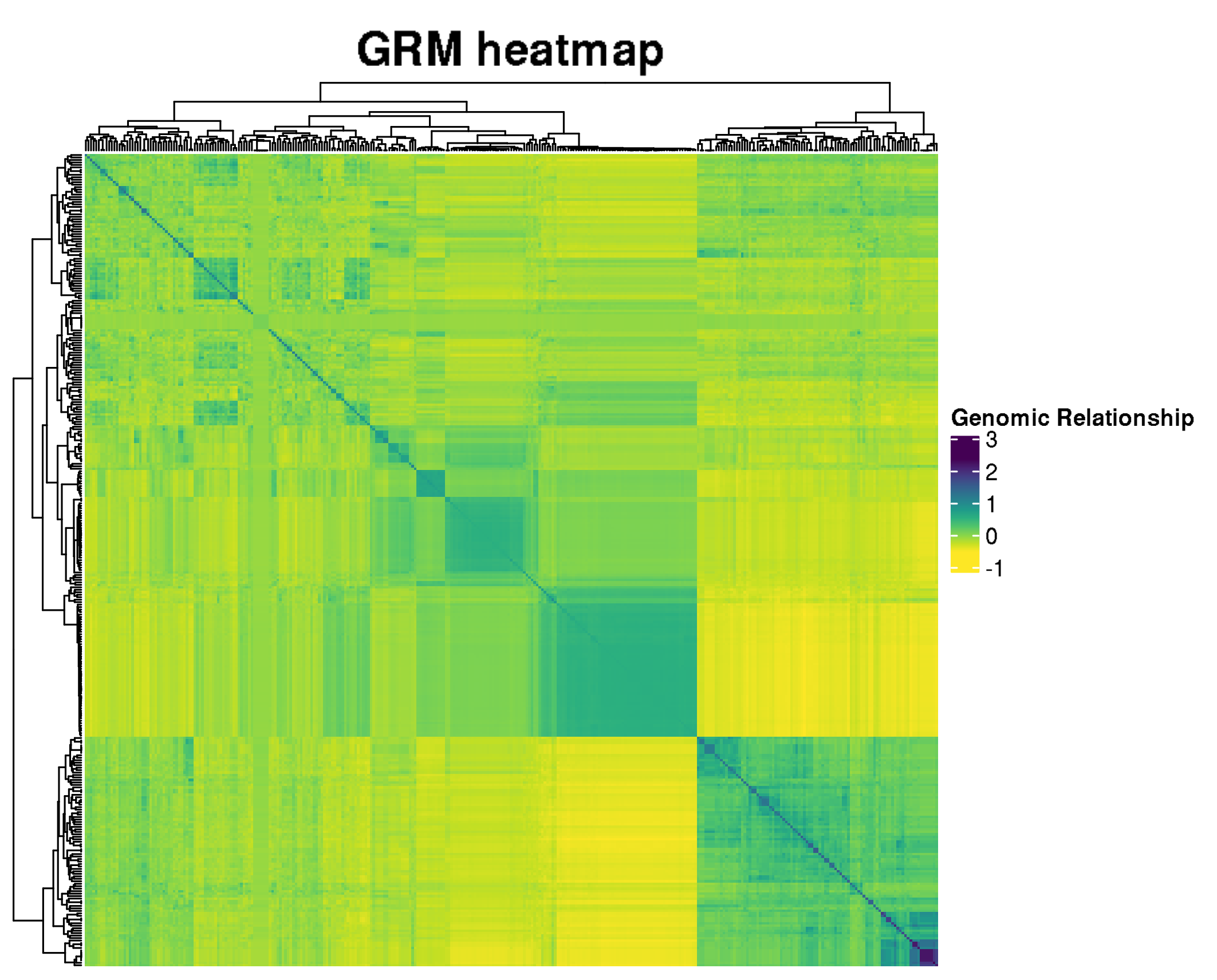

Supplement: S2 Fig — (TIF) [file pone.0294123.s002.tif]

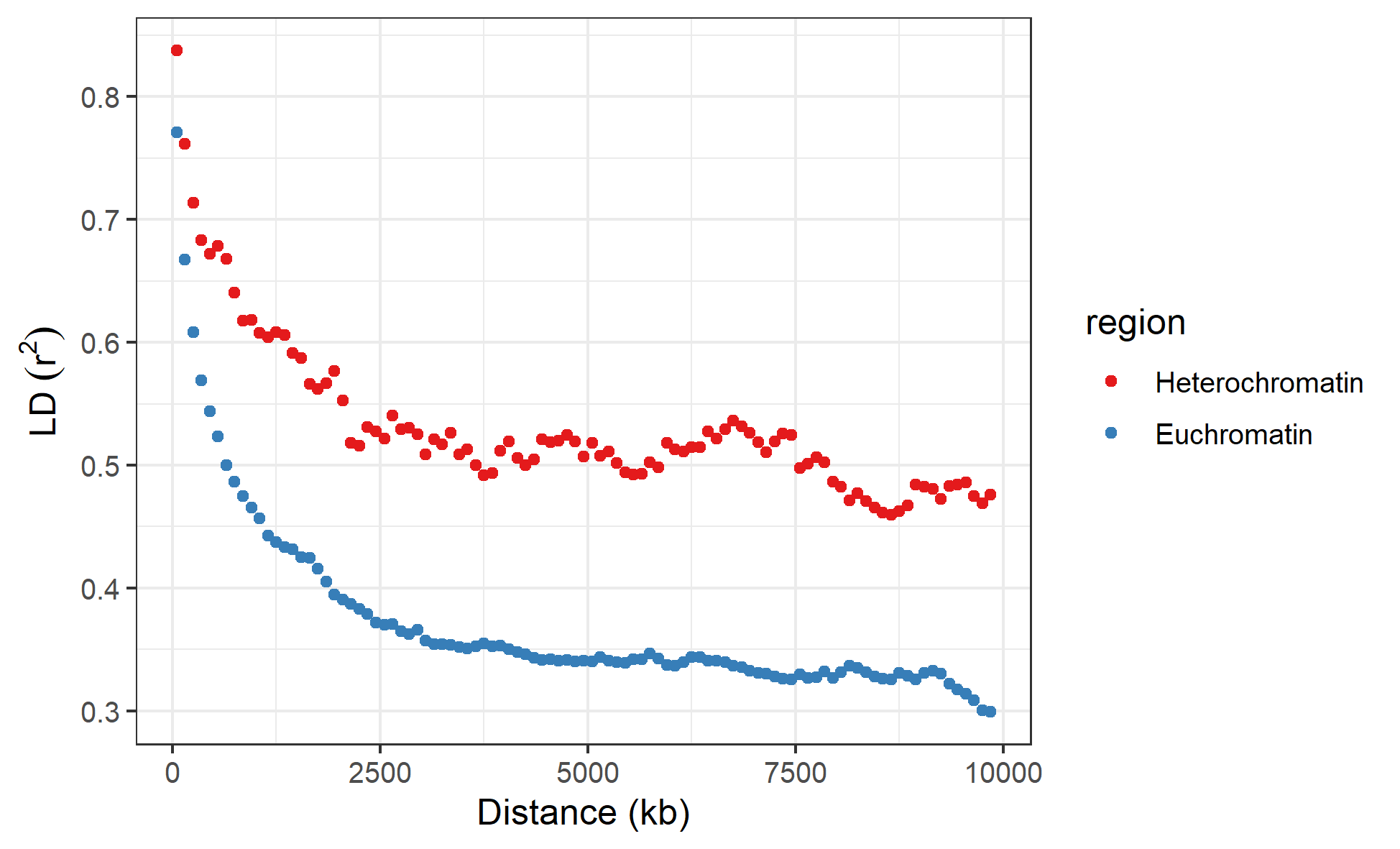

Supplement: S3 Fig — (TIF) [file pone.0294123.s003.tif]

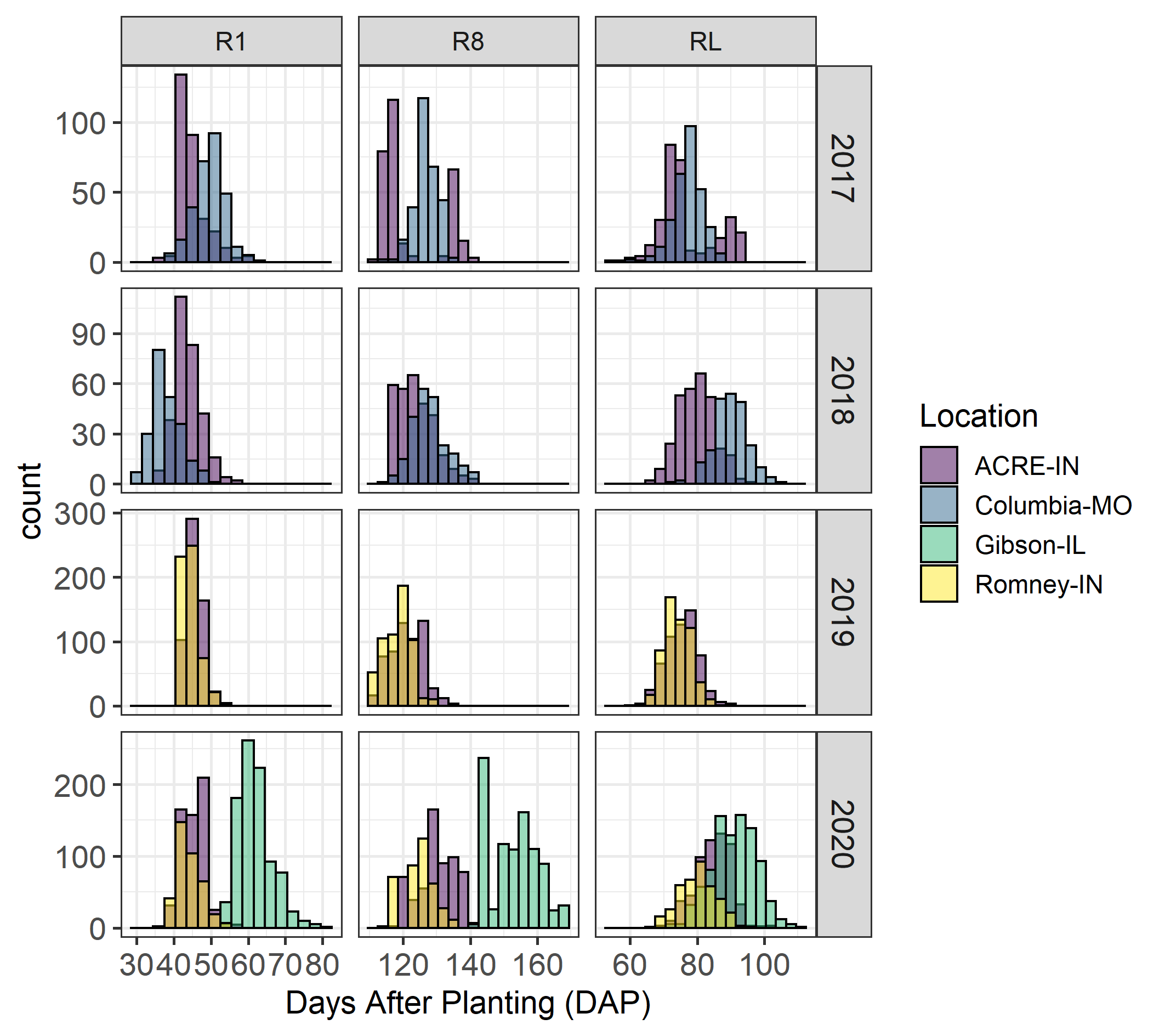

Supplement: S4 Fig — Data are measurements of R1 and R8 in days after planting (DAP), and RL as the number of days between R1 and R8 from four years and four locations. (TIF) [file pone.0294123.s004.tif]

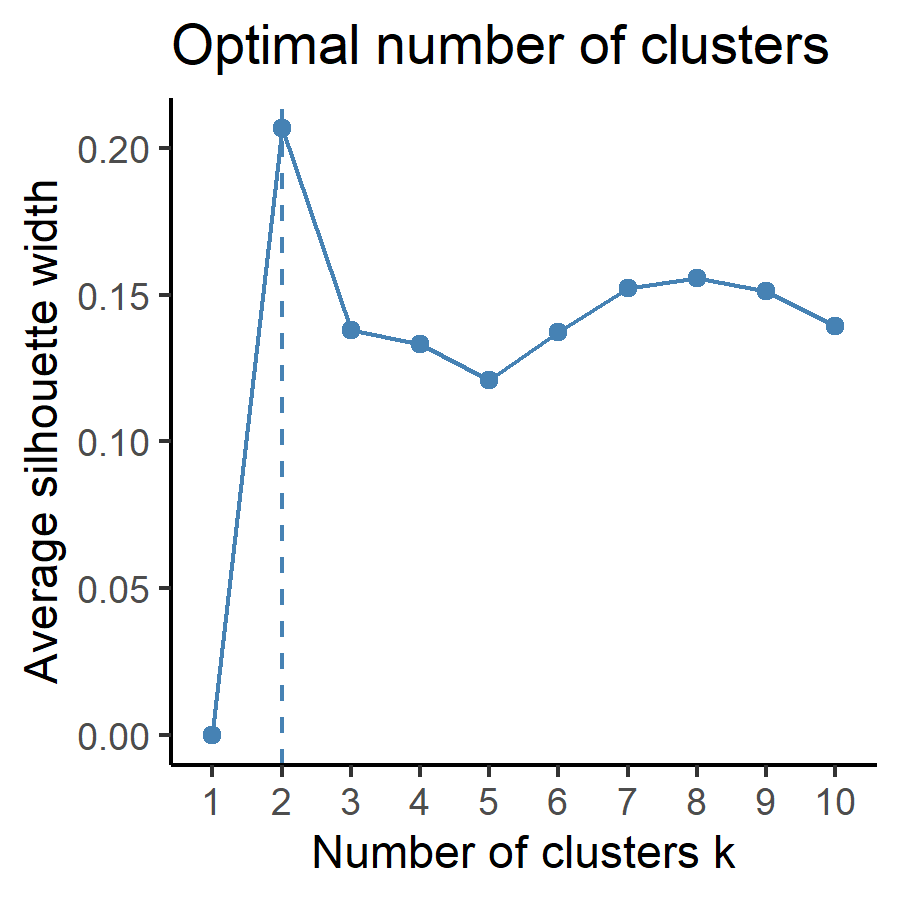

Supplement: S5 Fig — (TIF) [file pone.0294123.s005.tif]

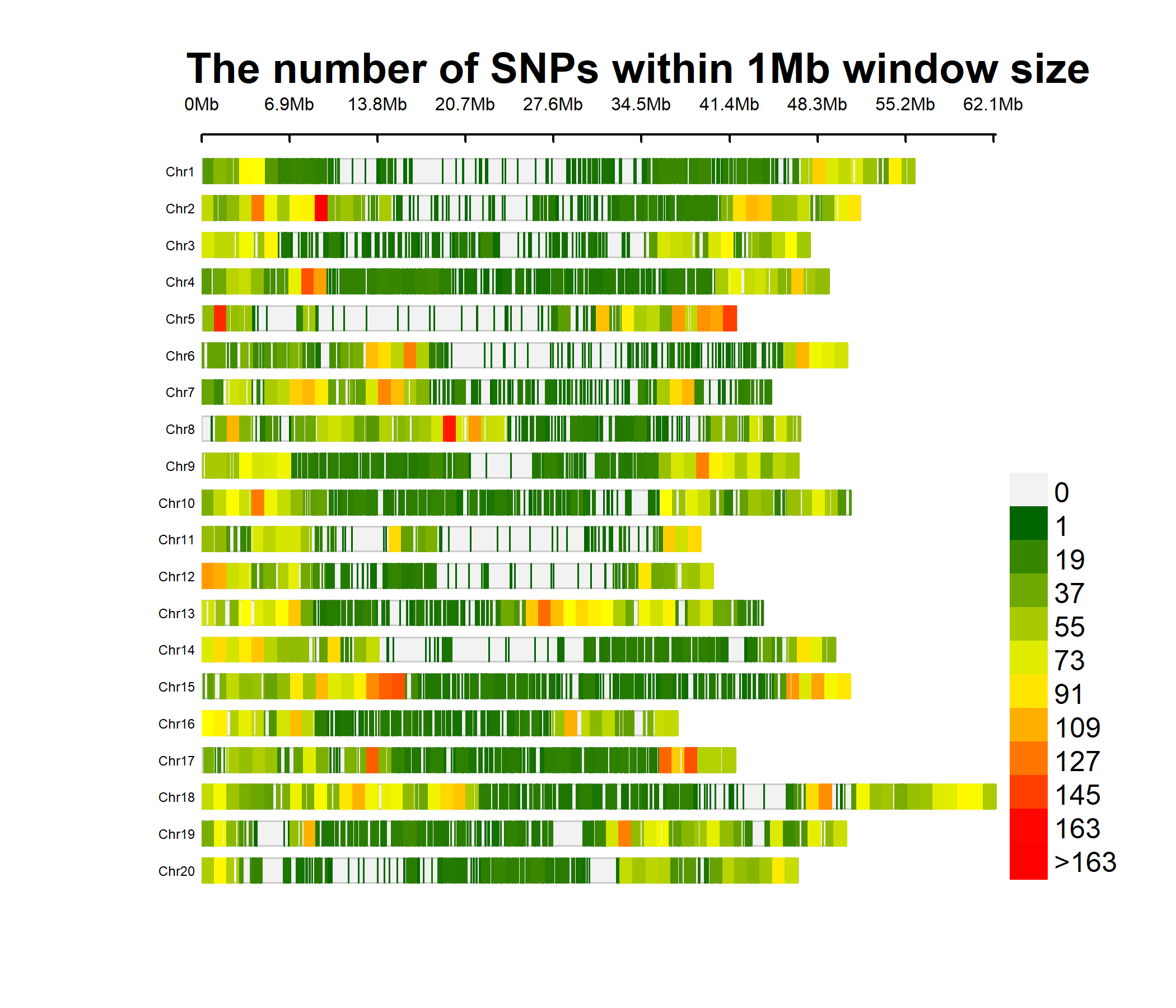

Supplement: S6 Fig — (TIF) [file pone.0294123.s006.tif]

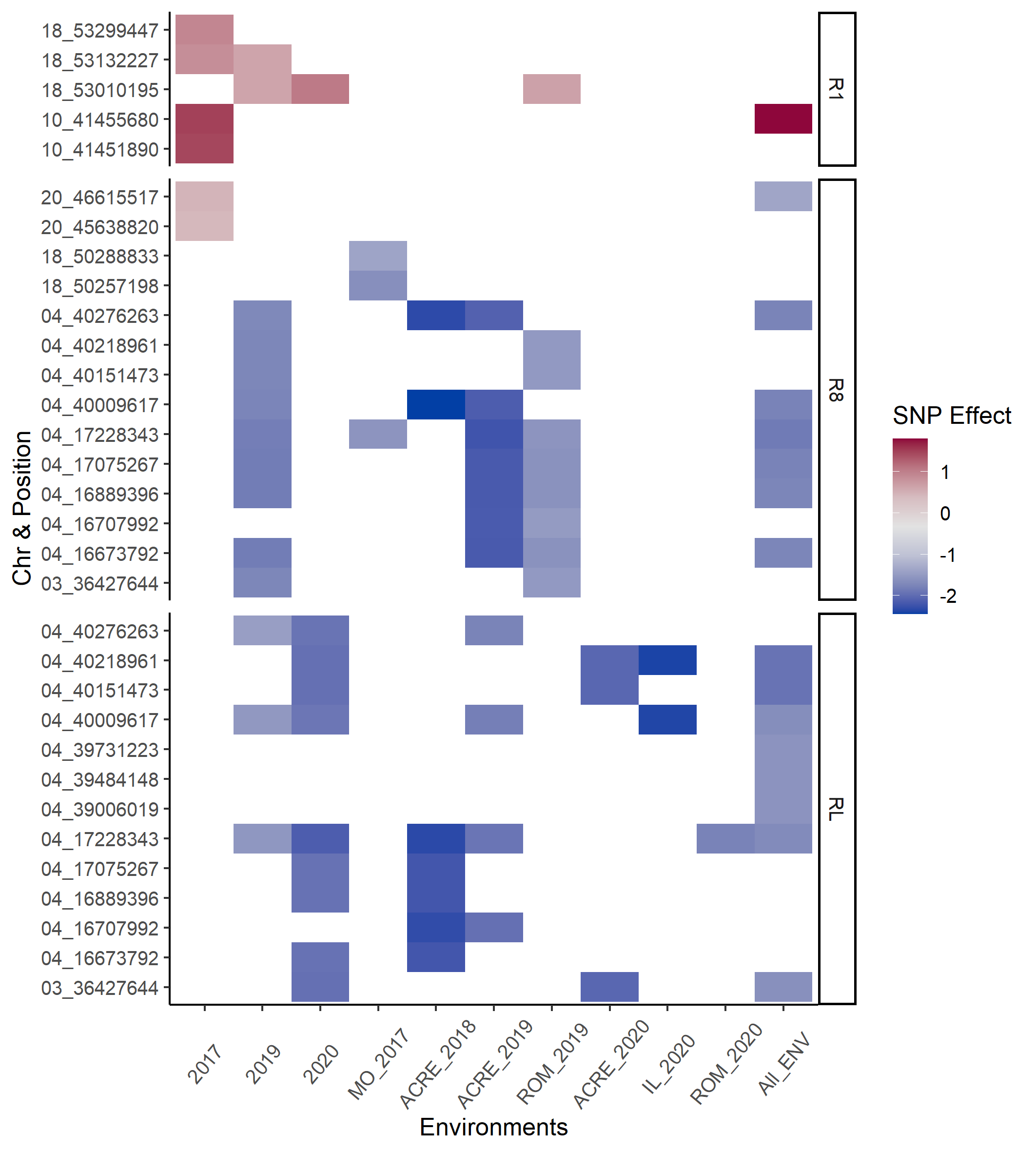

Supplement: S7 Fig — R1 is flowering time, R8 is maturity time, and RL is reproductive length. The plot is color coded by the effect of the SNPs for each trait. SNP positions are based on the Wm82.a2.v1 genome assembly. (TIF) [file pone.0294123.s007.tif]

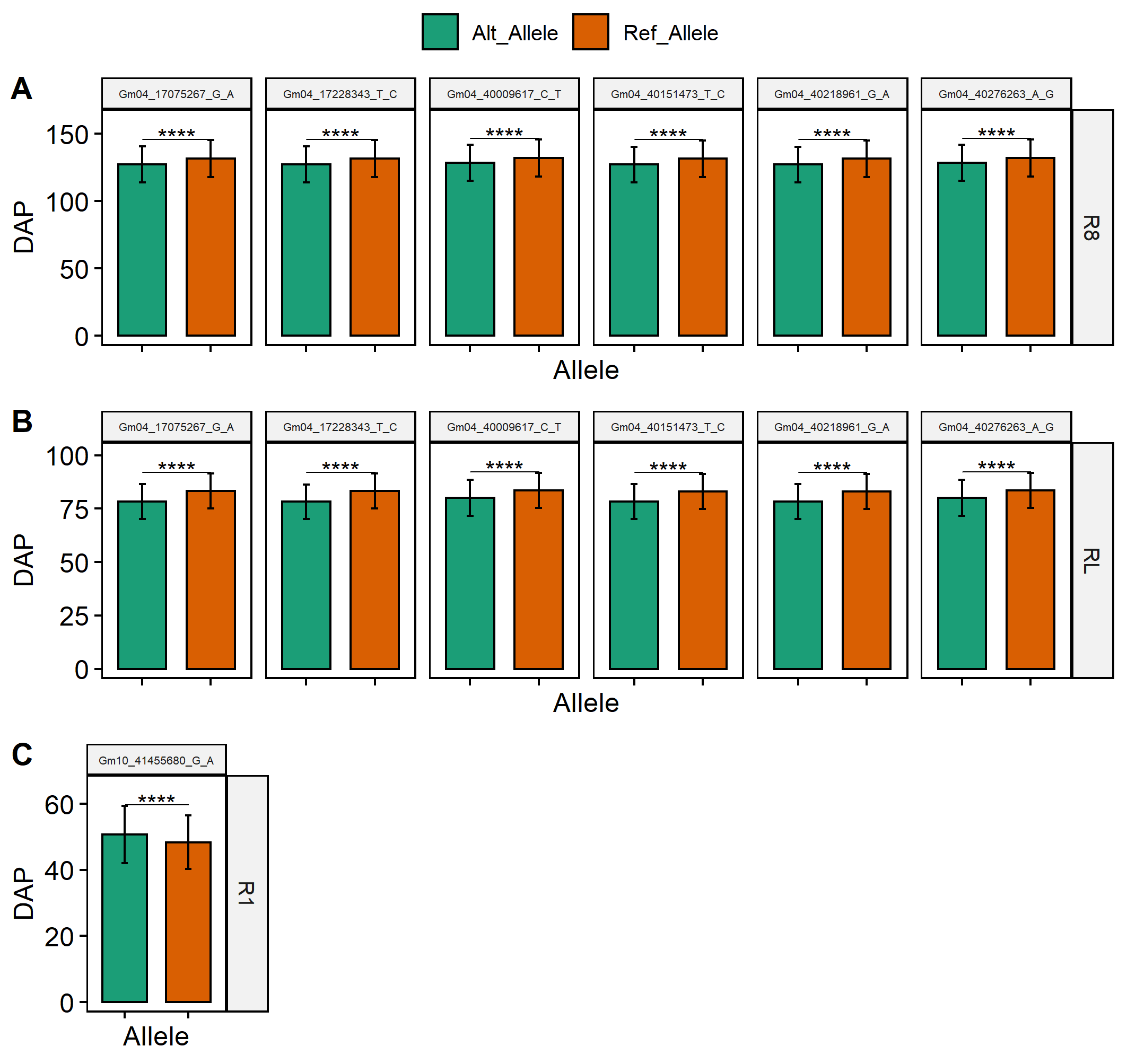

Supplement: S8 Fig — Bar plots show the differences in R8 (A), RL (B), and R1 (C). **** indicates significant differences at a p-value ≤ 0.0001 between the two groups. DAP is days after planting. (TIF) [file pone.0294123.s008.tif]
