# Supplementary material for: Genome-wide association study reveals GmFulb as candidate gene for maturity time and reproductive length in soybeans (Glycine max)
Source: PLoS One. 2024 Jan 19;19(1):e0294123. doi: 10.1371/journal.pone.0294123 (PMC10798547; doi:10.1371/journal.pone.0294123)
Supplement: S1 Table — (PDF) [file pone.0294123.s009.pdf]

S1 Table. Planting dates of experimental environments.

Environment[a]	Location	year	Planting date
**E1**	Columbia- MO	2017	16-May
**E2**	Columbia- MO	2018	15-May
**E3**	ACRE-IN	2017	18-May
**E4**	ACRE-IN	2018	22-May
**E5**	ACRE-IN	2019	4-Jun
**E6**	ACRE-IN	2020	24-May
**E7**	Romney-IN	2019	8-Jun
**E8**	Romney-IN	2020	27-May
**E9**	Gibson-Il	2020	23-Apr

[a] Individual environments represent the combination of location and year.
