# Supplementary material for: Genome-wide association study reveals GmFulb as candidate gene for maturity time and reproductive length in soybeans (Glycine max)
Source: PLoS One. 2024 Jan 19;19(1):e0294123. doi: 10.1371/journal.pone.0294123 (PMC10798547; doi:10.1371/journal.pone.0294123)
Supplement: S2 Table — (PDF) [file pone.0294123.s010.pdf]

S2 Table. Mean flowering (R1), and maturity (R8) time in days after planting (DAP) and mean reproductive length (RL) in number of days per accession across all environments.

Accession	R1		R8		RL		Accession	R1		R8		RL	
	mean	SD	mean	SD	mean	SD		mean	SD	mean	SD	mean	SD
**PI153243**	50.5	7.7	127.2	12.8	76.6	7.7	**PI548543**	4.6	128.7	5.5	88.3	5.5	4.6
**PI153292**	45.2	8.0	127.2	13.0	81.9	9.0	**PI548546**	9.0	131.9	15.1	80.1	8.5	9.0
**PI170380**	50.2	7.9	125.8	13.2	75.6	6.8	**PI548547**	6.8	131.1	13.1	81.9	8.4	6.8
**PI189926**	49.2	7.9	130.2	15.6	81.0	9.8	**PI548549**	8.4	133.7	14.8	84.9	9.2	8.4
**PI189928**	47.1	6.2	125.5	15.7	78.4	10.0	**PI548555**	9.0	138.1	14.2	89.9	6.8	9.0
**PI189935**	50.6	8.4	132.9	15.8	82.3	8.1	**PI548558**	6.4	126.7	12.3	79.3	7.6	6.4
**PI209331**	46.0	6.5	126.7	13.6	80.7	8.3	**PI548559**	NA	133.0	NA	83.0	NA	NA
**PI248402**	52.9	9.5	136.2	15.0	83.4	7.6	**PI548562**	6.7	122.8	12.0	76.8	6.4	6.7
**PI253660A**	47.5	5.3	126.3	12.8	78.9	8.5	**PI548574**	10.6	119.5	3.5	76.0	7.1	10.6
**PI253661B**	53.0	10.1	122.5	12.4	69.5	4.4	**PI548585**	7.4	129.4	12.4	82.6	8.0	7.4
**PI283331**	50.7	8.3	127.8	13.5	77.1	7.2	**PI548597**	7.1	122.2	12.3	77.5	8.0	7.1
**PI370055**	47.4	7.0	125.7	14.0	78.4	8.2	**PI548598**	8.3	137.1	15.5	87.1	9.9	8.3
**PI371611**	49.4	7.1	134.0	14.3	84.6	9.3	**PI548602**	11.0	138.3	15.8	84.9	7.8	11.0
**PI398881**	48.3	6.9	122.9	11.3	74.7	5.8	**PI548603**	10.1	136.1	14.7	82.6	6.5	10.1
**PI404173B**	55.9	11.1	134.6	15.6	78.7	7.1	**PI548613**	6.4	128.7	14.9	83.0	9.8	6.4
**PI417526**	48.5	6.7	127.7	14.5	79.2	8.8	**PI548614**	8.1	126.8	14.4	79.2	8.1	8.1
**PI417573**	50.5	8.7	130.5	14.2	80.0	8.5	**PI548622**	6.4	124.5	0.7	84.0	5.7	6.4
**PI424159B**	48.3	7.5	135.8	13.6	87.5	7.5	**PI548632**	7.3	126.1	12.0	78.6	7.4	7.3
**PI424405A**	46.2	7.2	128.7	11.5	82.5	6.4	**PI548633**	10.1	128.4	12.7	78.6	6.7	10.1
**PI424405B**	50.3	7.4	136.0	13.5	85.7	7.8	**PI548634**	9.9	121.5	3.5	78.5	6.4	9.9
**PI427136**	51.4	8.7	129.7	14.1	77.7	6.2	**PI548635**	8.7	127.0	10.8	78.3	3.3	8.7
**PI432359**	49.4	7.6	135.4	14.8	86.0	9.2	**PI548636**	10.3	134.9	14.5	85.3	6.2	10.3
**PI437103**	51.1	7.0	123.0	11.6	71.9	6.4	**PI548652**	7.5	129.4	11.8	83.4	6.1	7.5
**PI437124**	47.2	6.6	123.6	12.0	76.5	6.8	**PI548670**	10.1	131.5	16.2	82.4	9.0	10.1
**PI437317**	46.3	7.7	125.0	15.6	78.7	9.4	**PI548671**	6.3	134.5	16.2	79.5	10.7	6.3
**PI437338**	48.8	6.7	129.1	13.2	80.3	8.8	**PI548678**	9.6	138.1	16.4	81.0	8.7	9.6
**PI437357**	49.9	7.3	124.7	12.3	74.8	6.0	**PI548679**	10.1	138.2	15.4	85.5	8.9	10.1
**PI437359**	50.5	9.2	129.6	12.4	79.1	6.3	**PI548682**	7.1	134.0	14.6	86.1	9.8	7.1
**PI437365**	49.7	8.2	125.3	13.5	75.7	6.8	**PI548684**	9.8	126.3	14.8	77.6	7.5	9.8
**PI437367**	50.2	7.9	134.2	15.0	84.0	8.4	**PI548685**	6.7	127.3	13.0	81.5	7.6	6.7
**PI437377**	51.5	8.2	123.1	12.2	71.6	5.2	**PI548686**	9.2	124.7	14.4	75.7	6.4	9.2
**PI437388**	51.3	7.4	130.5	17.1	79.2	12.6	**PI552538**	8.4	124.6	11.0	79.6	7.7	8.4
**PI437391**	47.2	8.2	129.3	13.4	82.1	7.7	**PI555396**	9.5	134.1	14.8	85.4	7.4	9.5
**PI437392**	47.0	6.9	125.0	11.9	78.0	7.3	**PI555399**	8.5	139.9	14.6	85.5	8.4	8.5
**PI437397**	50.0	10.0	127.8	16.4	77.8	7.8	**PI556511**	7.3	124.9	12.6	81.2	8.5	7.3
**PI437412**	47.8	7.8	121.8	12.7	74.0	5.7	**PI556572**	8.6	133.3	14.3	86.7	7.9	8.6
**PI437422**	49.8	6.1	125.2	11.9	75.3	8.4	**PI556687**	6.8	130.0	12.8	83.7	8.0	6.8
**PI437444**	49.1	7.3	129.0	14.2	79.9	7.8	**PI556778**	10.4	129.4	11.8	81.8	5.8	10.4
**PI437459**	48.1	7.9	129.9	12.5	80.7	9.2	**PI556780**	7.9	135.7	13.9	88.4	8.3	7.9
**PI437461**	50.9	8.4	127.0	12.9	76.1	6.9	**PI556781**	8.5	129.3	13.0	82.8	8.4	8.5

**PI437463B**	45.5	5.9	121.7	12.0	76.2	7.1	**PI556814**	10.0	135.0	14.1	87.9	7.6	10.0
**PI437464**	48.2	7.0	131.8	15.7	83.6	9.4	**PI556816**	9.4	129.8	12.6	82.5	6.4	9.4
**PI437467**	48.8	8.4	126.8	10.7	77.9	5.0	**PI556857**	8.1	135.1	14.1	88.6	7.8	8.1
**PI437487**	49.6	7.8	130.6	14.9	81.0	9.5	**PI556859**	8.9	131.5	13.9	84.8	7.0	8.9
**PI437500B**	46.3	7.9	126.8	11.8	80.5	6.7	**PI556889**	10.5	139.5	14.6	89.1	5.9	10.5
**PI437500C**	45.6	7.3	122.3	11.8	76.7	6.7	**PI556928**	8.6	131.7	13.7	83.2	7.1	8.6
**PI437550C**	48.8	8.3	124.6	11.1	75.9	4.8	**PI556931**	7.3	126.1	10.8	81.9	6.8	7.3
**PI437641B**	47.6	7.2	122.8	12.9	75.3	7.3	**PI556932**	10.0	137.1	13.3	84.7	6.4	10.0
**PI437711B**	51.8	8.6	133.5	17.1	81.7	10.0	**PI556989**	7.3	124.7	10.2	78.7	5.0	7.3
**PI437788B**	54.0	NA	133.0	NA	79.0	NA	**PI559931**	7.3	128.3	5.3	85.8	6.4	7.3
**PI437789**	55.0	NA	133.0	NA	78.0	NA	**PI560206**	7.4	137.8	14.3	88.3	7.7	7.4
**PI437790**	53.0	NA	131.0	NA	78.0	NA	**PI560207**	7.7	137.4	16.3	85.1	10.3	7.7
**PI437877C**	43.0	NA	128.0	NA	85.0	NA	**PI561321**	6.8	125.2	15.3	79.7	9.5	6.8
**PI437888B**	51.0	NA	121.0	NA	70.0	NA	**PI561330A**	6.1	122.1	12.4	75.4	6.7	6.1
**PI437894**	47.6	6.5	128.3	13.4	80.7	7.8	**PI564718**	9.0	126.5	13.1	77.6	6.6	9.0
**PI437903**	52.0	9.7	125.5	11.3	73.5	5.1	**PI567250B**	NA	132.0	NA	79.0	NA	NA
**PI438073**	54.0	NA	133.0	NA	79.0	NA	**PI567351B**	9.0	135.5	16.6	83.0	9.2	9.0
**PI438079**	50.9	9.1	127.1	13.1	76.2	5.0	**PI567366A**	9.4	128.8	11.5	75.5	7.8	9.4
**PI438124B**	48.8	6.8	125.5	11.5	76.7	6.6	**PI567404A**	9.1	129.8	14.7	78.1	8.5	9.1
**PI438252C**	49.5	9.1	128.2	12.2	78.7	6.2	**PI567527**	9.4	137.8	14.4	85.7	6.1	9.4
**PI438303**	55.0	NA	132.0	NA	77.0	NA	**PI567767B**	8.1	133.0	12.3	85.1	5.8	8.1
**PI438310**	51.6	8.1	123.9	11.7	72.3	6.3	**PI568245**	7.2	132.1	12.9	84.9	8.7	7.2
**PI438312**	62.0	NA	123.0	NA	61.0	NA	**PI574532**	8.3	125.4	11.7	79.1	6.2	8.3
**PI438341**	44.5	8.2	123.5	13.1	78.9	8.3	**PI574534**	7.4	128.6	13.0	84.1	7.2	7.4
**PI438357B**	50.5	8.2	133.6	15.5	83.1	9.3	**PI574541**	6.8	133.5	14.8	84.5	8.7	6.8
**PI438409**	49.1	8.3	123.0	12.5	73.9	10.0	**PI576160**	7.3	127.7	14.6	81.8	9.1	7.3
**PI438486**	50.6	7.6	125.9	12.1	75.4	7.1	**PI576166**	10.0	136.3	12.9	88.5	6.3	10.0
**PI438495**	59.0	NA	129.0	NA	70.0	NA	**PI577798**	9.6	138.1	15.1	87.8	7.5	9.6
**PI438500**	47.3	8.1	129.1	10.6	81.8	6.2	**PI578057**	8.4	133.2	16.2	83.2	9.2	8.4
**PI475822B**	48.4	7.9	125.0	11.2	76.6	7.1	**PI584441**	7.7	130.2	14.5	82.1	8.0	7.7
**PI475822C**	56.6	11.4	136.5	18.1	79.8	9.8	**PI584470**	6.1	121.0	2.6	79.0	5.6	6.1
**PI479740**	45.9	7.5	124.6	10.8	78.7	6.8	**PI586980**	7.4	123.6	13.1	78.1	7.6	7.4
**PI507676**	47.8	8.1	123.3	12.8	75.5	7.4	**PI590932**	8.5	138.1	16.2	88.6	9.5	8.5
**PI507692B**	49.8	8.7	124.4	12.0	74.5	5.0	**PI591490**	7.4	138.1	17.0	87.8	12.0	7.4
**PI507696C**	49.4	8.7	144.3	16.6	94.9	8.7	**PI591492**	5.9	132.6	14.7	84.6	10.3	5.9
**PI507708**	47.3	7.3	124.9	12.4	77.6	5.7	**PI591493**	8.4	133.9	15.6	83.2	9.5	8.4
**PI515961**	50.5	9.0	141.4	14.4	90.9	7.2	**PI591494**	8.4	133.8	15.2	83.5	9.1	8.4
**PI518663**	46.9	7.3	129.1	14.4	82.2	8.9	**PI591495**	7.1	136.9	14.7	84.3	9.3	7.1
**PI518667**	46.0	NA	134.0	NA	88.0	NA	**PI591497**	7.1	132.1	11.7	85.1	6.4	7.1
**PI518668**	51.6	8.1	139.4	15.7	87.8	10.1	**PI591498**	7.3	130.6	13.3	82.2	8.1	7.3
**PI518671**	40.9	3.4	132.1	4.4	91.2	5.1	**PI591499**	7.4	132.8	15.2	83.7	9.5	7.4
**PI518673**	49.3	10.0	131.2	11.3	81.9	6.3	**PI591503**	8.1	129.9	11.4	82.5	6.6	8.1
**PI518675**	44.4	7.2	124.4	10.6	80.0	7.4	**PI591504**	9.2	130.3	14.2	82.1	6.4	9.2
**PI525454**	50.0	10.7	134.0	16.3	84.0	7.2	**PI591505**	8.3	131.1	14.9	82.7	8.5	8.3

**PI533654**	49.8	8.0	131.9	12.9	82.1	7.2	**PI591506**	6.3	127.8	9.8	82.6	6.9	6.3
**PI534645**	44.4	8.0	124.8	10.2	81.6	7.1	**PI591507**	7.4	131.5	14.1	83.6	8.5	7.4
**PI534646**	46.4	7.2	131.9	15.3	85.5	10.1	**PI591509**	8.8	130.6	13.7	83.6	7.8	8.8
**PI534647**	45.8	8.0	126.2	10.2	80.4	6.4	**PI591510**	7.4	128.8	14.4	81.4	8.6	7.4
**PI534648**	44.1	7.3	129.5	11.9	85.4	7.7	**PI591512**	8.1	128.1	11.9	80.9	5.7	8.1
**PI538401B**	49.3	7.0	135.1	14.9	85.8	9.2	**PI591513**	8.7	131.6	14.0	84.0	7.0	8.7
**PI540555**	46.5	7.7	131.4	12.0	84.9	7.6	**PI591515**	7.7	129.5	14.2	84.5	9.3	7.7
**PI542043**	47.8	8.6	131.8	13.3	85.4	8.4	**PI591516**	8.8	132.5	15.3	84.6	8.2	8.8
**PI542044**	47.6	7.9	128.7	11.5	81.1	6.3	**PI591517**	8.8	129.8	13.5	82.1	7.2	8.8
**PI542709**	47.2	8.1	127.1	11.2	79.9	6.1	**PI591518**	7.9	132.4	15.5	85.1	8.9	7.9
**PI542711**	46.2	7.0	130.7	11.9	84.5	8.2	**PI591519**	8.0	130.7	14.0	83.9	7.3	8.0
**PI543793**	48.3	7.8	137.9	13.6	89.7	9.0	**PI591521**	8.9	132.5	15.2	84.1	7.9	8.9
**PI547471**	52.5	8.6	136.1	16.4	83.6	9.4	**PI591526**	8.6	130.6	14.6	82.8	8.1	8.6
**PI547477**	49.5	6.8	135.0	14.6	85.5	9.4	**PI591528**	7.6	131.1	13.4	83.1	8.1	7.6
**PI547484**	47.7	7.3	128.1	13.2	80.4	7.8	**PI591530**	7.8	132.2	14.2	84.4	7.9	7.8
**PI547497**	48.8	5.8	133.5	15.2	84.8	10.6	**PI591531**	7.5	128.7	13.5	80.3	7.0	7.5
**PI547500**	49.3	8.7	128.8	13.1	79.5	8.3	**PI591532**	8.9	131.0	13.8	81.4	9.2	8.9
**PI547501**	45.6	6.7	127.7	14.3	82.1	8.2	**PI591533**	7.6	128.1	11.8	81.8	6.8	7.6
**PI547545**	49.8	6.8	134.1	13.4	84.3	8.5	**PI591534**	6.7	124.1	10.9	79.0	6.0	6.7
**PI547598**	48.9	6.7	133.6	13.4	84.7	9.2	**PI591535**	7.2	130.7	14.3	82.1	7.9	7.2
**PI547617**	50.3	9.4	133.7	14.5	83.4	8.0	**PI591536**	8.4	135.1	15.5	86.1	8.7	8.4
**PI547655**	48.7	6.5	131.4	10.7	82.7	6.4	**PI591537**	7.4	133.6	15.5	86.9	10.4	7.4
**PI547657**	48.9	8.3	132.5	14.2	83.6	7.7	**PI591538**	7.4	129.3	14.1	82.8	8.6	7.4
**PI547663**	47.5	6.8	126.7	10.4	79.2	7.0	**PI591539**	7.4	130.1	14.6	82.4	8.3	7.4
**PI547798**	47.0	7.3	125.9	12.3	78.9	6.7	**PI591540**	7.5	126.9	11.9	79.8	7.4	7.5
**PI547800**	49.0	8.6	125.9	10.8	76.9	4.7	**PI591561**	8.9	131.8	15.7	85.7	10.4	8.9
**PI547801**	49.8	6.1	128.0	12.1	78.2	9.3	**PI592946**	9.9	129.9	15.0	78.9	7.1	9.9
**PI547802**	49.2	7.6	129.4	13.8	80.2	7.8	**PI593256**	9.2	134.9	14.6	86.2	6.8	9.2
**PI547811**	46.1	7.6	123.5	10.6	78.5	8.2	**PI593258**	7.8	130.3	13.3	85.3	7.2	7.8
**PI547813**	47.9	7.8	127.5	15.6	79.6	9.0	**PI593463**	8.0	127.6	11.4	80.8	5.2	8.0
**PI547814**	46.5	7.4	124.8	11.3	78.3	6.5	**PI593654**	6.5	126.9	11.6	81.6	7.6	6.5
**PI547847**	49.2	8.1	131.3	14.2	82.1	7.4	**PI595363**	7.9	128.0	14.6	83.1	9.6	7.9
**PI547860**	47.1	7.7	131.3	14.5	84.2	8.8	**PI595754**	7.3	128.1	12.1	80.5	6.9	7.3
**PI547864**	46.8	8.5	131.1	14.1	84.3	7.7	**PI595926**	7.5	122.7	11.2	77.4	6.7	7.5
**PI547869**	48.1	7.6	129.6	12.2	81.5	6.8	**PI596407**	7.4	125.2	10.5	77.3	5.8	7.4
**PI547876**	48.0	8.7	133.7	14.3	85.7	7.9	**PI597382**	8.0	133.6	13.4	87.4	7.0	8.0
**PI547879**	50.9	9.5	132.9	13.6	82.0	6.0	**PI597387**	7.5	132.1	14.6	86.6	8.5	7.5
**PI547885**	48.1	8.3	130.3	14.6	82.1	7.6	**PI598124**	5.7	126.0	1.4	91.0	4.2	5.7
**PI547886**	47.8	7.5	130.9	14.3	83.1	8.0	**PI602449**	9.2	133.0	12.8	82.3	5.7	9.2
**PI548174**	48.5	6.6	129.1	14.3	80.5	8.1	**PI602450**	7.8	133.3	12.8	81.8	7.6	7.8
**PI548177**	50.6	7.6	130.2	12.0	79.6	7.0	**PI603186**	7.2	130.4	13.6	78.9	9.7	7.2
**PI548180**	48.5	6.6	123.3	12.0	74.8	6.4	**PI603188**	7.8	129.7	12.0	80.1	7.1	7.8
**PI548191**	52.5	9.7	134.2	16.6	81.6	8.5	**PI603193**	6.9	131.4	12.4	81.9	8.2	6.9
**PI548192**	50.7	6.4	135.4	14.1	84.7	9.2	**PI603201**	8.8	138.0	16.7	80.2	9.9	8.8

**PI548193**	51.8	9.2	135.6	15.8	83.5	7.6	**PI603205**	7.5	134.2	12.8	82.6	6.4	7.5
**PI548194**	50.7	9.9	132.2	14.6	81.5	6.6	**PI603396**	8.0	127.5	14.3	74.1	7.3	8.0
**PI548198**	55.0	8.5	119.5	3.5	64.5	12.0	**PI603428D**	6.5	128.1	11.5	79.2	7.0	6.5
**PI548204**	50.0	5.6	124.5	14.5	74.5	10.5	**PI603434**	7.0	124.2	11.1	76.7	5.4	7.0
**PI548222**	49.9	10.2	136.8	14.9	86.9	7.5	**PI603442**	7.7	122.4	12.5	77.9	6.0	7.7
**PI548231**	45.9	6.4	127.6	11.4	81.7	6.9	**PI603454**	8.0	132.4	13.6	83.6	7.7	8.0
**PI548242**	47.0	8.2	128.5	13.4	81.4	7.3	**PI603564A**	6.2	133.0	12.1	82.8	9.6	6.2
**PI548247**	49.7	7.4	131.6	13.0	81.9	7.2	**PI603564B**	8.2	136.8	14.6	82.9	8.0	8.2
**PI548250**	55.1	11.1	139.8	14.4	84.7	6.0	**PI603564C**	7.4	137.4	15.7	85.9	10.6	7.4
**PI548253**	47.3	8.0	132.1	14.2	84.8	8.2	**PI603571B**	9.5	133.6	12.7	85.6	7.0	9.5
**PI548260**	54.3	5.5	131.0	3.6	76.7	7.5	**PI603915D**	8.8	133.5	13.7	85.6	6.5	8.8
**PI548262**	46.5	8.7	131.4	13.3	84.9	6.5	**PI606748**	8.2	135.8	13.5	89.4	8.1	8.2
**PI548267**	52.1	7.0	131.7	11.8	79.7	7.8	**PI606749**	7.6	139.2	14.0	91.1	7.5	7.6
**PI548275**	49.0	7.7	132.5	13.9	83.5	7.6	**PI610670**	7.2	126.5	12.3	79.0	6.3	7.2
**PI548286**	47.7	9.3	132.2	13.2	84.5	7.0	**PI612594**	7.1	130.0	13.7	85.0	8.0	7.1
**PI548297**	51.8	9.1	135.0	14.7	83.2	6.8	**PI612750**	7.4	124.3	13.1	80.3	8.0	7.4
**PI548304**	46.2	6.4	123.8	13.2	77.5	7.7	**PI612932**	7.8	123.4	11.4	77.1	5.2	7.8
**PI548333**	47.9	9.3	134.5	13.6	86.6	7.9	**PI614153**	7.2	130.8	12.5	86.6	7.8	7.2
**PI548339**	52.2	8.7	133.7	16.0	81.5	10.5	**PI614154**	8.0	125.6	10.5	81.8	6.1	8.0
**PI548340**	49.0	10.2	128.0	14.5	80.8	7.6	**PI614155**	8.2	134.6	12.0	88.1	6.7	8.2
**PI548365**	46.8	8.1	126.2	16.2	79.3	8.9	**PI614832**	7.9	126.8	11.5	81.3	5.8	7.9
**PI548366**	48.8	8.7	121.2	12.0	73.9	6.1	**PI615555**	7.7	127.7	12.1	80.8	6.2	7.7
**PI548367**	48.6	8.9	127.4	13.1	78.8	6.9	**PI615556**	6.4	131.5	14.0	82.5	8.3	6.4
**PI548373**	49.7	7.3	127.0	14.9	77.3	9.0	**PI633729**	7.8	131.2	14.0	83.9	7.3	7.8
**PI548381**	46.7	7.7	122.1	12.1	75.4	6.0	**PI633732**	7.3	133.9	15.3	89.4	10.3	7.3
**PI548383**	49.4	6.5	129.0	14.7	79.6	10.1	**PI634761**	8.2	128.1	13.4	79.9	8.1	8.2
**PI548400**	52.7	10.1	135.7	15.2	83.0	6.1	**PI634762**	7.3	131.1	12.7	81.0	7.9	7.3
**PI548410**	51.0	8.5	134.4	15.2	83.4	9.1	**PI634763**	8.8	134.4	13.4	83.6	7.1	8.8
**PI548412**	47.3	8.2	130.8	14.4	83.5	7.7	**PI634764**	8.1	129.2	16.3	80.1	10.1	8.1
**PI548421**	48.3	6.3	129.0	14.7	80.7	8.9	**PI634765**	7.5	130.4	14.2	81.5	8.5	7.5
**PI548517**	49.8	11.5	133.5	17.1	83.7	9.5	**PI636464**	7.9	127.7	10.2	81.3	5.8	7.9
**PI548518**	46.9	6.2	126.1	14.0	79.2	8.8	**PI636695**	7.0	135.0	14.9	84.7	11.2	7.0
**PI548522**	43.8	4.6	123.5	7.5	79.8	10.7	**PI636696**	11.2	140.4	13.5	84.3	6.9	11.2
**PI548525**	50.6	9.0	127.4	14.1	76.8	5.9	**PI639740**	10.6	130.5	0.7	86.0	9.9	10.6
**PI548532**	49.7	7.8	130.3	13.0	80.7	7.4	**PI642055**	9.4	140.7	16.2	86.3	8.7	9.4
**PI548541**	53.3	9.0	139.1	14.7	85.8	7.1	**PI643146**	8.5	128.5	0.7	86.5	9.2	8.5
**PI548542**	46.4	7.5	126.3	11.6	79.9	6.2							

NA is present when the accession had only one data point and the standard deviation (sd) could not be estimated.
