# Supplementary material for: Genome-wide association study reveals GmFulb as candidate gene for maturity time and reproductive length in soybeans (Glycine max)
Source: PLoS One. 2024 Jan 19;19(1):e0294123. doi: 10.1371/journal.pone.0294123 (PMC10798547; doi:10.1371/journal.pone.0294123)
Supplement: S3 Table — (PDF) [file pone.0294123.s011.pdf]

S3 Table. Descriptive statistics of phenotypic variation, genotypic variance (G) and broad-sense heritability (H^2) of days to flowering (R1), days to maturity (R8), and reproductive length for 329 *G.* max USDA accessions evaluated at nine environments.

Trait	Environment[a]	Year	Location	min	max	mean	sd	CV	H entry (%)	h^2 (%)
**R1**	All environments		All environments	31	80	48.5	8.2	16.9	65	60
**R1**	E1	2017	Columbia-MO	39	62	49.6	4.1	8.2		
**R1**	E2	2018	Columbia-MO	31	51	37.9	3.9	10.2		
**R1**	E3	2017	ACRE-IN	36	61	45.2	3.8	8.4		
**R1**	E4	2018	ACRE-IN	36	57	43.8	3.6	8.2		
**R1**	E5	2019	ACRE-IN	42	55	45.6	2.2	4.8		
**R1**	E6	2020	ACRE-IN	37	57	45.4	3.1	6.9		
**R1**	E7	2019	Romney-IN	41	53	44.4	2.2	4.9		
**R1**	E8	2020	Romney-IN	37	54	44.2	3.2	7.1		
**R1**	E9	2020	Gibson-IL	54	80	61.9	4.4	7.1		
**R8**	All environments		All environments	110	169	130.2	13.7	10.5	69	54
**R8**	E1	2017	Columbia-MO	115	134	126.9	3.4	2.7		
**R8**	E2	2018	Columbia-MO	116	142	128.0	5.3	4.2		
**R8**	E3	2017	ACRE-IN	112	140	121.9	8.9	7.4		
**R8**	E4	2018	ACRE-IN	115	142	123.7	5.56	4.5		
**R8**	E5	2019	ACRE-IN	110	135	121.1	4.8	3.9		
**R8**	E6	2020	ACRE-IN	117	141	129.9	5.7	4.4		
**R8**	E7	2019	Romney-IN	110	136	120.8	5.3	4.4		
**R8**	E8	2020	Romney-IN	114	136	124.4	4.9	3.9		
**R8**	E9	2020	Gibson-IL	141	169	152.9	7.2	4.7		
**RL**	All environments		All environments	55	111	81.7	8.46	10.4	57	50
**RL**	E1	2017	Columbia-MO	60	88	77.29	4.4	5.7		
**RL**	E2	2018	Columbia-MO	73	105	90.03	5.03	5.6		
**RL**	E3	2017	ACRE-IN	55	94	76.79	8.46	11.0		
**RL**	E4	2018	ACRE-IN	67	95	79.89	5.35	6.7		
**RL**	E5	2019	ACRE-IN	63	89	45.47	4.57	10.1		
**RL**	E6	2020	ACRE-IN	65	102	84.67	5.35	6.3		
**RL**	E7	2019	Romney-IN	61	87	76.46	5.46	7.1		
**RL**	E8	2020	Romney-IN	66	95	80.14	5.33	6.7		
**RL**	E9	2020	Gibson-IL	71	111	90.96	6.48	7.1		

[a] Individual environments represent the combination of location and year.
