# Supplementary material for: Genome-wide association study reveals GmFulb as candidate gene for maturity time and reproductive length in soybeans (Glycine max)
Source: PLoS One. 2024 Jan 19;19(1):e0294123. doi: 10.1371/journal.pone.0294123 (PMC10798547; doi:10.1371/journal.pone.0294123)
Supplement: S4 Table — (PDF) [file pone.0294123.s012.pdf]

S4 Table. Percentage of SNPs by heterochromatic and euchromatic regions of each chromosome.

Chromosome	Percentage of SNPs (%)	
	Heterochromatin	Euchromatin
1	31	69
2	13	87
3	23	77
4	39	61
5	14	86
6	11	89
7	11	89
8	15	85
9	41	59
10	32	68
11	36	64
12	25	75
13	29	71
14	38	62
15	15	85
16	22	78
17	22	78
18	16	84
19	33	67
20	33	67
Total	24	76
