# Supplementary material for: Genome-wide association study reveals GmFulb as candidate gene for maturity time and reproductive length in soybeans (Glycine max)
Source: PLoS One. 2024 Jan 19;19(1):e0294123. doi: 10.1371/journal.pone.0294123 (PMC10798547; doi:10.1371/journal.pone.0294123)
Supplement: S5 Table — (PDF) [file pone.0294123.s013.pdf]

S5 Table. Linkage Disequilibrium (LD) decay rate across 20 chromosomes within euchromatic and heterochromatic regions.

Chr	LD decay rate (kb)	
	**Euchromatic**	**Heterochromatic**
1	350	8350
2	2250	4350
3	1450	4750
4	450	9150
5	2250	7550
6	1450	8750
7	6450	9550
8	8250	8650
9	650	6050
10	2350	8250
11	650	4350
12	450	7850
13	3750	7450
14	250	8850
15	1850	9750
16	650	7950
17	1550	6050
18	2650	5150
19	3550	9650
20	9450	7750
Across all	2150	8650
