# Supplementary material for: Genome-wide association study reveals GmFulb as candidate gene for maturity time and reproductive length in soybeans (Glycine max)
Source: PLoS One. 2024 Jan 19;19(1):e0294123. doi: 10.1371/journal.pone.0294123 (PMC10798547; doi:10.1371/journal.pone.0294123)
Supplement: S6 Table — (PDF) [file pone.0294123.s014.pdf]

S6 Table. Summary of single-nucleotide polymorphisms (SNPs) significantly associated with flowering time (R1), maturity time (R8), and reproductive length (RL) in three hundred twenty-nine G. max accessions across environments and years.

Chr	SNP Wm82.a2[a]	SoySNP50k_ID[a]	Chr Reg[b]	Loc Genome [a]	Trait	-log(p-val)	Var Exp	Eff	Reported QTLs[a]	Known *E* Genes [c]	Distance (kb) from known *E* genes[c]
**10**	Gm10_41455680_G_A *	ss715607080	Eu	Int	R1	4.4	0.05	1.79	R8 full maturity 10-g4.1, Reproductive stage length 4-g2.1, Reproductive stage length 4-g2.2, Reproductive period 3-g5, Seed protein 7-g7 and seed weight 4-g10	E2	3839
**4**	Gm04_17228343_T_C *	ss715587206	Het	Int	R8	4.71	0.02	-1.85	-	E1La E1Lb	19530 8892
**4**	Gm04_17075267_G_A *	ss715587203	Het	Int	R8	4.4	0.02	-1.77	-	E1La E1Lb	19683 9045
**4**	Gm04_40009617_C_T *	ss715587845	Het	Int	R8	4.66	0.02	-1.77	-	E1La E1Lb	3251 13889
**4**	Gm04_40276263_A_G *	ss715587857	Het	Int	R8	4.7	0.02	-1.76	-	E1La E1Lb	3517 26120
**4**	Gm04_16673792_G_A	ss715587192	Het	Int	R8	4.4	0.02	-1.77	-	E1La E1Lb	20084 9446
**4**	Gm04_16889396_T_C	ss715587197	Het	Int	R8	4.4	0.01 9	-1.77	-	E1La E1Lb	19869 9231
**20**	Gm20_46615517_A_C	ss715638773	Eu	Int	R8	4.33	0.02	-1.36	First flower 6-g4, R8 full maturity 8-g13, Reproductive period 3-g1	E4	13374
**4**	Gm04_39977826_G_A	ss715587844	Het	Int	R8	4.17	0.01	-1.6	-	E1La E1Lb	3219 13857
**4**	Gm04_40151473_T_C *	ss715587852	Het	Int	R8	4.18	0.01	-1.68	-	E1La E1Lb	3393 14031
**4**	Gm04_40218961_G_A *	ss715587856	Het	Int	R8	4.18	0.01	-1.68	-	E1La E1Lb	3461 14098
**3**	Gm03_36427644_C_T	ss715585727	Het	CDS	RL	4.17	0.04	-1.62	First flower 4-g10, First flower 3-g2, R8 full maturity 3-g3	-	-

**4**	Gm04_40151473_T_C<br>*	ss715587852	Het	Int	RL	5.63	0.05	-1.92	-	E1La<br>E1Lb	3393<br>14031
**4**	Gm04_40218961_G_A<br>*	ss715587856	Het	Int	RL	5.63	0.05	-1.92	-	E1La<br>E1Lb	3461<br>14098
**4**	Gm04_17228343_T_C<br>*	ss715587206	Het	Int	RL	4.24	0.04	-1.68	-	E1La<br>E1Lb	19530<br>8892
**4**	Gm04_40009617_C_T<br>*	ss715587845	Het	Int	RL	4.35	0.04	-1.65	-	E1La<br>E1Lb	3251<br>13889
**4**	Gm04_39006019_T_C	ss715587808	Het	Int	RL	4.22	0.04	-1.58	-	E1La<br>E1Lb	2248<br>12885
**4**	Gm04_39484148_T_C	ss715587823	Het	Int	RL	4.22	0.04	-1.58	-	E1La<br>E1Lb	2726<br>13364
**4**	Gm04_39731223_A_G	ss715587834	Het	Int	RL	4.22	0.04	-1.58	-	E1La<br>E1Lb	2972<br>13611

Chr is chromosome, SNP Wm82.a2 is the SNP positions based on Wm82.a2, Chr Reg is chromosomal region where SNP is located, Loc Genome is the location in the genome, Var Exp is variance explained, Eff is the SNP effect, Het is Heterochromatin, Eu is Euchromatin, Int is intergenic, CDS coding sequence, R8 is days to maturity, RL is reproductive length, R1 days to flowering, [a] Information obtained from SoyBase; [b] Information obtained from Wen et al., (2015); [c] Information obtained from Zimmer et al., (2021); * SNPs selected for candidate gene selection (tagging SNPs).
