# Supplementary material for: Genome-wide association study reveals GmFulb as candidate gene for maturity time and reproductive length in soybeans (Glycine max)
Source: PLoS One. 2024 Jan 19;19(1):e0294123. doi: 10.1371/journal.pone.0294123 (PMC10798547; doi:10.1371/journal.pone.0294123)
Supplement: S7 Table — (PDF) [file pone.0294123.s015.pdf]

S7 Table. Summary of significant single-nucleotide polymorphisms (SNPs) associated with flowering time (R1), maturity time (R8), and reproductive length (RL) in three hundred twenty-nine G. max accessions by individual environments, years, and across all environments and years.

Chr	SNP ID[a]	Pos Wm82.a1[b]	Pos Wm82.a2[b]	Chr Reg[c]	Loc Genome[b]	Env[d]	Trait	-log(p-val)	Var Exp	Eff
**Gm10**	40905094_T_C	40905094	41451890	Eu	Int	2017	R1	4.23	0.11	1.45
**Gm10**	40908884_G_A	40908884	41455680	Eu	Int	2017	R1	4.46	0.115	1.49
**Gm10**	40908884_G_A	40908884	41455680	Eu	Int	All_ENV	R1	4.4	0.05	1.79
**Gm18**	57279697_A_G	57279697	53010195	Eu	Int	Romney, IN_2019	R1	4.13	0.09	0.65
**Gm18**	57279697_A_G	57279697	53010195	Eu	Int	2019	R1	4.24	0.651	0.62
**Gm18**	57279697_A_G	57279697	53010195	Eu	Int	2020	R1	4.25	0.013	1.05
**Gm18**	57401711_A_G	57401711	53132227	Eu	Int	2017	R1	4.6	0.037	0.85
**Gm18**	57401711_A_G	57401711	53132227	Eu	Int	2019	R1	4.21	0.649	0.62
**Gm18**	57569299_A_G	57569299	53299447	Eu	Int	2017	R1	4.34	0.045	0.93
**Gm03**	38448001_C_T	38448001	36427644	Eu	CDS	Romney, IN_2019	R8	4.64	0.15	-1.51
**Gm03**	38448001_C_T	38448001	36427644	Eu	CDS	2019	R8	4.37	0.15	-1.73
**Gm04**	15633879_G_A	15633879	16673792	Het	Int	ACRE, IN_2019	R8	4.19	0.22	-2.16
**Gm04**	15633879_G_A	15633879	16673792	Het	Int	Romney, IN_2019	R8	4.98	0.17	-1.60
**Gm04**	15633879_G_A	15633879	16673792	Het	Int	2019	R8	4.28	0.073	-1.83
**Gm04**	15633879_G_A	15633879	16673792	Het	Int	All_ENV	R8	4.4	0.02	-1.74
**Gm04**	15667941_T_C	15667941	16707992	Het	Int	ACRE, IN_2019	R8	4.22	0.21	-2.15
**Gm04**	15667941_T_C	15667941	16707992	Het	Int	Romney, IN_2019	R8	4.47	0.15	-1.49
**Gm04**	15849345_T_C	15849345	16889396	Het	Int	ACRE, IN_2019	R8	4.19	0.22	-2.16
**Gm04**	15849345_T_C	15849345	16889396	Het	Int	Romney, IN_2019	R8	4.98	0.17	-1.60
**Gm04**	15849345_T_C	15849345	16889396	Het	Int	2019	R8	4.60	0.17	-1.83
**Gm04**	15849345_T_C	15849345	16889396	Het	Int	All_ENV	R8	4.25	0.019	-1.74
**Gm04**	16031274_G_A	16031274	17075267	Het	Int	ACRE, IN_2019	R8	4.19	1.18	-2.16
**Gm04**	16031274_G_A	16031274	17075267	Het	Int	Romney, IN_2019	R8	4.98	0.17	-1.60
**Gm04**	16031274_G_A	16031274	17075267	Het	Int	2019	R8	4.60	0.17	-1.83
**Gm04**	16031274_G_A	16031274	17075267	Het	Int	All_ENV	R8	4.4	0.02	-1.77
**Gm04**	16183920_T_C	16183920	17228343	Het	Int	Columbia, MO_2017	R8	4.32	0.45	-1.57
**Gm04**	16183920_T_C	16183920	17228343	Het	Int	ACRE, IN_2019	R8	4.33	0.23	-2.22
**Gm04**	16183920_T_C	16183920	17228343	Het	Int	Romney, IN_2019	R8	4.76	0.16	-1.57
**Gm04**	16183920_T_C	16183920	17228343	Het	Int	2019	R8	4.53	0.17	-1.82

**Gm04**	16183920_T_C	16183920	17228343	Het	Int	All_ENV	R8	4.71	0.02	-1.85
**Gm04**	36874657_C_T	36874657	40009617	Het	Int	ACRE, IN_2018	R8	4.51	0.20	-2.44
**Gm04**	36874657_C_T	36874657	40009617	Het	Int	ACRE, IN_2019	R8	4.32	0.21	-2.13
**Gm04**	36874657_C_T	36874657	40009617	Het	Int	2019	R8	4.55	0.20	-1.75
**Gm04**	36874657_C_T	36874657	40009617	Het	Int	All_ENV	R8	4.66	0.02	-1.77
**Gm04**	37010886_T_C	37010886	40151473	Het	Int	Romney, IN_2019	R8	4.64	0.15	-1.51
**Gm04**	37010886_T_C	37010886	40151473	Het	Int	2019	R8	4.37	0.15	-1.73
**Gm04**	37078558_G_A	37078558	40218961	Het	Int	Romney, IN_2019	R8	4.64	0.15	-1.51
**Gm04**	37078558_G_A	37078558	40218961	Het	Int	2019	R8	4.37	0.15	-1.73
**Gm04**	37126858_A_G	37126858	40276263	Het	Int	ACRE, IN_2018	R8	4.20	0.19	-2.32
**Gm04**	37126858_A_G	37126858	40276263	Het	Int	ACRE, IN_2019	R8	4.27	0.20	-2.09
**Gm04**	37126858_A_G	37126858	40276263	Het	Int	2019	R8	4.47	0.15	-1.71
**Gm04**	37126858_A_G	37126858	40276263	Het	Int	All_ENV	R8	4.68	0.02	-1.76
**Gm18**	54531027_T_C	54531027	50257198	Eu	CDS	Columbia, MO_2017	R8	5.00	0.24	-1.63
**Gm18**	54562662_T_C	54562662	50288833	Eu	CDS	Columbia, MO_2017	R8	4.73	0.17	-1.38
**Gm20**	44509848_C_T	44509848	45638820	Eu	5UTR	2017	R8	4.19	0.003	0.42
**Gm20**	45487288_A_C	45487288	46615517	Eu	Int	2017	R8	4.88	0.004	0.46
**Gm20**	45487288_A_C	45487288	46615517	Eu	Int	All_ENV	R8	4.33	0.02	-1.36
**Gm03**	38448001_C_T	38448001	36427644	Eu	CDS	ACRE, IN_2020	RL	4.51	0.15	-2.04
**Gm03**	38448001_C_T	38448001	36427644	Eu	CDS	2020	RL	4.49	0.07	-1.95
**Gm03**	38448001_C_T	38448001	36427644	Eu	CDS	All_ENV	RL	4.17	0.04	-1.62
**Gm04**	15633879_G_A	15633879	16673792	Het	Int	ACRE, IN_2018	RL	4.92	0.18	-2.20
**Gm04**	15633879_G_A	15633879	16673792	Het	Int	2020	RL	4.26	0.07	-1.93
**Gm04**	15667941_T_C	15667941	16707992	Het	Int	ACRE, IN 2018	RL	5.41	0.19	-2.30
**Gm04**	15667941_T_C	15667941	16707992	Het	Int	ACRE, IN_2019	RL	4.65	0.19	-1.95
**Gm04**	15849345_T_C	15849345	16889396	Het	Int	ACRE, IN_2018	RL	4.92	0.18	-2.20
**Gm04**	15849345_T_C	15849345	16889396	Het	Int	2020	RL	4.26	0.07	-1.93
**Gm04**	16031274_G_A	16031274	17075267	Het	Int	ACRE, IN_2018	RL	4.92	0.18	-2.20
**Gm04**	16031274_G_A	16031274	17075267	Het	Int	2020	RL	4.26	0.07	-1.93
**Gm04**	16183920_T_C	16183920	17228343	Het	Int	ACRE, IN_2018	RL	5.37	0.20	-2.33
**Gm04**	16183920_T_C	16183920	17228343	Het	Int	ACRE, IN_2019	RL	4.31	0.18	-1.90
**Gm04**	16183920_T_C	16183920	17228343	Het	Int	Romney, IN_2020	RL	4.23	0.12	-1.77
**Gm04**	16183920_T_C	16183920	17228343	Het	Int	2019	RL	4.31	0.13	-1.54
**Gm04**	16183920_T_C	16183920	17228343	Het	Int	2020	RL	5.00	0.08	-2.13

**Gm04**	16183920_T_C	16183920	17228343	Het	Int	All_ENV	RL	4.24	0.04	-1.68
**Gm04**	35879410_T_C	35879410	39006019	Het	Int	All_ENV	RL	4.22	0.04	-1.58
**Gm04**	36357346_T_C	36357346	39484148	Het	Int	All_ENV	RL	4.22	0.04	-1.58
**Gm04**	36604337_A_G	36604337	39731223	Het	Int	All_ENV	RL	4.22	0.04	-1.58
**Gm04**	36874657_C_T	36874657	40009617	Het	Int	ACRE, IN_2019	RL	4.29	0.17	-1.81
**Gm04**	36874657_C_T	36874657	40009617	Het	Int	Gibson, IL_2020	RL	4.54	0.11	-2.37
**Gm04**	36874657_C_T	36874657	40009617	Het	Int	2019	RL	4.58	0.13	-1.52
**Gm04**	36874657_C_T	36874657	40009617	Het	Int	2020	RL	4.40	0.06	-1.89
**Gm04**	36874657_C_T	36874657	40009617	Het	Int	All_ENV	RL	4.35	0.04	-1.65
**Gm04**	37010886_T_C	37010886	40151473	Het	Int	ACRE, IN_2020	RL	4.51	0.15	-2.04
**Gm04**	37010886_T_C	37010886	40151473	Het	Int	2020	RL	4.49	0.07	-1.95
**Gm04**	37010886_T_C	37010886	40151473	Het	Int	All_ENV	RL	5.63	0.05	-1.92
**Gm04**	37078558_G_A	37078558	40218961	Het	Int	ACRE, IN_2020	RL	4.51	0.15	-2.04
**Gm04**	37078558_G_A	37078558	40218961	Het	Int	Gibson, IL_2020	RL	4.63	0.11	-2.39
**Gm04**	37078558_G_A	37078558	40218961	Het	Int	2020	RL	4.49	0.07	-1.95
**Gm04**	37078558_G_A	37078558	40218961	Het	Int	All_ENV	RL	5.63	0.05	-1.92
**Gm04**	37126858_A_G	37126858	40276263	Het	Int	ACRE, IN 2019	RL	4.15	0.16	-1.76
**Gm04**	37126858_A_G	37126858	40276263	Het	Int	2019	RL	4.26	0.12	-1.45
**Gm04**	37126858_A_G	37126858	40276263	Het	Int	2020	RL	4.56	0.07	-1.91

Chr is chromosome, Pos Wm82.a1 is SNPs position in Wm82.a1.v1 genome assembly, Pos Wm82.a2 is SNPs position in Wm82.a2.v1 genome assembly, Chr Reg is chromosomal region where SNP is located, Loc Genome is the location in the genome, Var Exp is variance explained, Eff is the SNP effect, Het is Heterochromatin, Eu is Euchromatin, Int is intergenic, CDS coding sequence, R8 is days to maturity, RL is reproductive length, R1 days to flowering, [a]all SNP IDs start with "BARC_1.01_Gm" and the number of chromosome and the positions are based on Wm82.a1.v1 genome assembly; [b]Information obtained from SoyBase; [c] Information obtained from Wen et al., (2015); [d]The significant SNPs were obtained from the association analysis of individual environments, years, and across all environments and years; * SNPs selected for candidate gene selection (tagging SNPs).
