# Supplementary material for: Genome-wide association study reveals GmFulb as candidate gene for maturity time and reproductive length in soybeans (Glycine max)
Source: PLoS One. 2024 Jan 19;19(1):e0294123. doi: 10.1371/journal.pone.0294123 (PMC10798547; doi:10.1371/journal.pone.0294123)
Supplement: S8 Table — (PDF) [file pone.0294123.s016.pdf]

S8 Table. List of potential candidate genes by tagging SNPs with average accuracy and descriptions for flowering time (R1), maturity time (R8), and reproductive length (RL).

Gene	Chr	Gene Pos[a]	Tag SNPs ID	QTL peak	Trait	Avg Acc %[c]	Description	Gene name TAIR[d]	Gene Bank ID[e]
Glyma.04g123700	Gm04	16002630-16006606	15633879_G_A	Peak 1	R8/RL	87.60	Solute: sodium symporters, urea transmembrane transporters	AT5G45380.1	-
Glyma.04g123800	Gm04	16010092-16013287	15633879_G_A	Peak 1	R8/RL	87.31	Alternative oxidase	-	-
Glyma.04g123900	Gm04	16016060-16026862	15633879_G_A	Peak 1	R8/RL	86.76	Heteroglycan glucosidase 1	AT3G23640.1	-
Glyma.04g124000	Gm04	16034262-16035430	15633879_G_A	Peak 1	R8/RL	87.52	Unknown protein	-	-
Glyma.04g124100	Gm04	16077062-16078008	15633879_G_A/16031274_G_A	Peak 1	R8/RL	88.70	Disease resistance-responsive (dirigent-like protein) family protein	AT1G65870.1	-
Glyma.04g124200	Gm04	16077062-16078008	15633879_G_A/16031274_G_A	Peak 1	R8/RL	88.55	BZIP transcription factor/ABA-responsive element binding protein 3	AT3G56850.1	-
Glyma.04g124300	Gm04	16092707-16099713	15633879_G_A/16031274_G_A	Peak 1	R8/RL	88.40	Far-red elongated hypocotyls 3	AT3G22170.1	-
Glyma.04g124400	Gm04	16123330-16126794	15633879_G_A/16031274_G_A	Peak 1	R8/RL	88.91	Unknown protein	-	-
Glyma.04g124500	Gm04	16284712-16286641	15633879_G_A/16031274_G_A	Peak 1	R8/RL	88.50	Protein of unknown function (DUF1191)	AT4G22900.1	-
Glyma.04g124600	Gm04	16329983-16332898	15633879_G_A/16031274_G_A	Peak 1	R8/RL	88.32	FAR1-related sequence 5	AT4G38180.1	-
Glyma.04g124700	Gm04	16346707-16347780	15633879_G_A	Peak 1	R8/RL	86.00	Unknown protein	-	-
Glyma.04g124800	Gm04	16368438-16390781	15633879_G_A/16031274_G_A	Peak 1	R8/RL	89.60	Zinc induced facilitator-like 1	AT5G13750.1	-
Glyma.04g124900	Gm04	16399212-16402624	15633879_G_A/16031274_G_A	Peak 1	R8/RL	90.30	Uncharacterized protein LOC102668196 isoform X1 [Glycine max]	AT1G48560.2	-
Glyma.04g125000	Gm04	16409044-16409645	15633879_G_A/16031274_G_A	Peak 1	R8/RL	89.70	NB-ARC domain-containing disease resistance protein	AT3G07040.1	-
Glyma.04g125100	Gm04	16472994-16473578	15633879_G_A/16031274_G_A	Peak 1	R8/RL	89.63	SOS3-interacting protein 3	AT4G30960.1	-

Glyma.04g125200	Gm04	16474623-16475810	15633879_G_A/16031274_G_A	Peak 1	R8/RL	89.86	Regulator of Vps4 activity in the MVB pathway protein	AT4G35730.1	-
Glyma.04g125300	Gm04	16500908-16508912	15633879_G_A/16031274_G_A	Peak 1	R8/RL	89.43	NB-ARC domain-containing disease resistance protein	AT3G07040.1	-
Glyma.04g125400	Gm04	16529335-16529723	15633879_G_A/16031274_G_A	Peak 1	R8/RL	88.70	GRF zinc finger	-	-
Glyma.04g125500	Gm04	16532221-16536664	15633879_G_A/16031274_G_A	Peak 1	R8/RL	90.00	SU(VAR)3-9 homolog 3	AT1G73100.1	-
Glyma.04g125600	Gm04	16622379-16634652	15633879_G_A/16031274_G_A	Peak 1	R8/RL	94.75	Zinc ion binding	AT3G54360.1	-
Glyma.04g125700	Gm04	16709512-16714749	15633879_G_A/16031274_G_A	Peak 1	R8/RL	91.97	Myb domain protein 33	AT5G06100.2	-
Glyma.04g125800	Gm04	16726595-16738293	15633879_G_A/16031274_G_A	Peak 1	R8/RL	91.82	Vacuolar protein sorting 26A	AT5G53530.1	-
Glyma.04g125900	Gm04	16809159-16809753	15633879_G_A/16031274_G_A	Peak 1	R8/RL	94.19	Protein kinase superfamily protein	AT5G56790.1	-
Glyma.04g126000	Gm04	16811865-16817931	15633879_G_A/16031274_G_A	Peak 1	R8/RL	92.78	Protein kinase superfamily protein	AT5G56790.1	-
Glyma.04g126100	Gm04	16937746-16942191	15633879_G_A/16031274_G_A	Peak 1	R8/RL	92.50	Unknown protein	AT2G38695.1	-
Glyma.04g126300	Gm04	16964286-16965431	15633879_G_A	Peak 1	R8/RL	85.30	Membrane steroid binding protein 1	AT5G52240.1	-
Glyma.04g126400	Gm04	17000680-17005885	15633879_G_A/16031274_G_A	Peak 1	R8/RL	95.23	Global transcription factor group B1	AT1G65440.2	-
Glyma.04g126500	Gm04	17010198-17015041	15633879_G_A/16183920_T_C	Peak 1	R8/RL	92.51	Multidrug resistance-associated protein 2	AT2G34660.1	-
Glyma.04g126600	Gm04	17030276-17031139	16183920_T_C/15633879_G_A	Peak 1	R8/RL	93.20	BTB/POZ domain-containing protein	AT4G10800.1	-
Glyma.04g126700	Gm04	17057705-17060281	16183920_T_C	Peak 1	R8/RL	92.46	ARM repeat superfamily protein	AT1G12930.1	-
Glyma.04g126800	Gm04	17060524-17063365	15633879_G_A	Peak 1	R8/RL	90.89	Translation initiation factor 2, small GTP-binding protein	AT4G11160.1	-
Glyma.04g126900	Gm04	17096980-17102005	15633879_G_A/16031274_G_A	Peak 1	R8/RL	94.50	Uridine-ribohydrolase 1	AT2G36310.1	-
Glyma.04g127000	Gm04	17123614-17124372	15633879_G_A/16031274_G_A/16183920_T_C	Peak 1	R8/RL	94.45	CBS domain-containing protein with a domain of unknown function (DUF21	AT1G03270.1	-

Glyma.04g127100	Gm04	17126079-17130669	15633879_G_A/16031274_G_A/16183920_T_C	Peak 1	R8/RL	93.98	Cysteine-rich RLK (RECEPTOR-like protein kinase) 10	AT4G23180.1	-
Glyma.04g127200	Gm04	17136690-17142016	15633879_G_A/16031274_G_A/16183920_T_C	Peak 1	R8/RL	93.24	Unknown protein	-	-
Glyma.04g127300	Gm04	17182350-17182837	15633879_G_A/16031274_G_A/16183920_T_C	Peak 1	R8/RL	94.46	Unknown protein	-	-
Glyma.04g127400	Gm04	17255113-17256083	15633879_G_A/16031274_G_A	Peak 1	R8/RL	89.18	Unknown protein	AT4G02550.2	-
Glyma.04g127500	Gm04	17256467-1725,923	15633879_G_A/16031274_G_A/16183920_T_C	Peak 1	R8/RL	92.49	Pentatricopeptide repeat (PPR) superfamily protein	AT1G11290.1	-
Glyma.04g127600	Gm04	17386901-17387809	15633879_G_A/16031274_G_A	Peak 1	R8/RL	89.17	C2H2 and C2HC zinc fingers superfamily protein	AT5G10970.1	-
Glyma.04g127700	Gm04	17503761-17506319	16031274_G_A15633879_G_A	Peak 1	R8/RL	88.00	BED zinc finger, hAT family dimerisation domain	AT3G42170.1	-
Glyma.04g127800	Gm04	17506510-17506734	15633879_G_A/16031274_G_A	Peak 1	R8/RL	89.45	Unknown protein	-	-
Glyma.04g127900	Gm04	17508855-17512554	15633879_G_A	Peak 1	R8/RL	87.18	Nodulin MtN21 /EamA-like transporter family protein	AT5G07050.1	-
Glyma.04g128000	Gm04	17687258-17689497	16031274_G_A	Peak 1	R8/RL	90.80	Unknown protein	-	-
Glyma.04g128400	Gm04	17759715-17762227	16031274_G_A	Peak 1	R8/RL	91.18	Zinc ion binding	AT2G44580.1	-
Glyma.04g128500	Gm04	17809136-17810282	16031274_G_A	Peak 1	R8/RL	90.20	Late embryogenesis abundant protein, group 1 protein	AT1G32560.1	-
Glyma.04g128600	Gm04	17866415-17867698	16031274_G_A	Peak 1	R8/RL	90.25	Alpha/beta-Hydrolases superfamily protein	AT1G68620.1	-
Glyma.04g128700	Gm04	17902621-17908873	16031274_G_A	Peak 1	R8/RL	89.04	SCP1-like small phosphatase 4	AT5G46410.2	-
Glyma.04g128800	Gm04	17927529-17936517	16031274_G_A	Peak 1	R8/RL	88.93	C2H2 zinc-finger protein SERRATE (SE)	AT2G27100.1	-
Glyma.04g128900	Gm04	17931394-17931507	16031274_G_A	Peak 1	R8/RL	89.10	Unknown protein	-	-
Glyma.04g129000	Gm04	17964578-17969250	16031274_G_A	Peak 1	R8/RL	89.90	Unknown protein	AT5G04910.1	-
Glyma.04g129100	Gm04	17974120-17976827	16031274_G_A/16183920_T_C	Peak 1	R8/RL	90.92	Tetratricopeptide repeat (TPR)-like superfamily protein	AT3G57430.1	-

Glyma.04g129200	Gm04	18028813-18036924	16031274_G_A	Peak 1	R8/RL	91.29	Aminophospholipid ATPase 1	<u>AT5G04930.1</u>	-
Glyma.04g129300	Gm04	18076546-18077311	16031274_G_A	Peak 1	R8/RL	89.80	GRAM domain family protein	AT4G01600.1	-
Glyma.04g159300	Gm04	39283912-39295261	37078558_G_A	Peak 2	R8/RL	90.80	AGAMOUS-like 8/MADS-box transcription factor 6 [Glycine max]	<u>AT5G60910.1</u>	-
Glyma.04g159400	Gm04	39320127-39321864	37078558_G_A	Peak 2	R8/RL	90.40	Unknown protein	-	-
Glyma.04g159500	Gm04	39451100-39453905	37078558_G_A	Peak 2	R8/RL	90.85	Saposin B domain-containing protein	AT3G51730.1	-
Glyma.04g159600	Gm04	39578853-39583603	37078558_G_A	Peak 2	R8/RL	90.50	Squamosa promoter-binding protein-like (SBP domain) transcription factor family protein	AT1G69170.1	-
Glyma.04g159700	Gm04	39586882-39589922	37078558_G_A	Peak 2	R8/RL	90.40	Reversibly glycosylated polypeptide 3	AT3G08900.1	-
Glyma.04g159800	Gm04	39601379-39606991	37078558_G_A	Peak 2	R8/RL	90.93	PIF1-like helicase	-	-
Glyma.04g159900	Gm04	39633309-39634727	37078558_G_A	Peak 2	R8/RL	90.73	Tetratricopeptide repeat (TPR)-like superfamily protein	<u>AT3G23020.1</u>	-
Glyma.04g160000	Gm04	39655881-39658301	37078558_G_A	Peak 2	R8/RL	91.17	COBRA-like extracellular glycosyl-phosphatidyl inositol-anchored protein family	AT5G15630.1	-
Glyma.04g160200	Gm04	39725839-39726383	37010886_T_C/ 37078558_G_A	Peak 2	R8/RL	89.42	Unknown protein	<u>AT1G21280.1</u>	-
Glyma.04g160300	Gm04	39737018-39741052	37010886_T_C/ 37078558_G_A37126858_A_G	Peak 2	R8/RL	83.33	Transducin/WD40 repeat-like superfamily protein	AT5G60940.1	-
Glyma.04g160500	Gm04	39750017-39752209	37078558_G_A	Peak 2	R8/RL	90.23	WAPL (Wings apart-like protein regulation of heterochromatin) protein	AT1G11060.1	-
Glyma.04g160600	Gm04	39809314-39814127	37078558_G_A	Peak 2	R8/RL	90.30	PIF1 helicase	AT3G51690.1	-
Glyma.04g160700	Gm04	39818871-39825716	37078558_G_A	Peak 2	R8/RL	89.90	Galactose oxidase/kelch repeat superfamily protein	AT1G18610.1	-
Glyma.04g160800	Gm04	39840898-39854321	37078558_G_A	Peak 2	R8/RL	90.20	Histone-lysine N-methyltransferases	AT3G26850.1	-
Glyma.04g161000	Gm04	39923410-39928355	37010886_T_C37078558_G_A	Peak 2	R8/RL	92.00	LONGIFOLIA 2-like isoform X5 [Glycine max];	AT1G74160.1	-

Glyma.04g161200	Gm04	39942189-39946987	37078558_G_A	Peak 2	R8/RL	89.51	Ribosomal protein L31e family protein	AT2G19740.1	-
Glyma.04g161300	Gm04	39978926-39980814	37126858_A_G	Peak 2	R8/RL	86.04	Ribosomal protein L1p/L10e family)	AT1G08360.1	-
Glyma.04g161400	Gm04	40001447-40004338	37078558_G_A	Peak 2	R8/RL	93.75	TEOSINTE BRANCHED 1, cycloidea and PCF transcription factor 5)	AT5G60970.1	-
Glyma.04g161500	Gm04	40052142-40060432	37126858_A_G/36874657_C_T	Peak 2	R8/RL	85.00	PIF1 helicase	AT3G51700.1	-
Glyma.04g161600	Gm04	40088117-40090295	36874657_C_T	Peak 2	R8/RL	94.40	Protein of unknown function (DUF1118))	AT5G08050.1	-
Glyma.04g161700	Gm04	40104836-40105385	37010886_T_C37078558_G_A	Peak 2	R8/RL	92.50	Myb/SANT-like DNA-binding domain protein	AT4G02550.2	-
Glyma.04g161800	Gm04	40109124-40109663	37010886_T_C/37078558_G_A	Peak 2	R8/RL	95.00	(Cyclin/Brf1-like TBP-binding protein)	AT3G09360.1	-
Glyma.04g161900	Gm04	40147800-40148585	37010886_T_C37078558_G_A	Peak 2	R8/RL	93.81	Unknown protein	-	-
Glyma.04g162000	Gm04	40157051..40157476	37010886_T_C37078558_G_A	Peak 2	R8/RL	91.89	Transmembrane protein	AT1G53035.1	-
Glyma.04g162200	Gm04	40212903-40217728	37010886_T_C37078558_G_A	Peak 2	R8/RL	97.00	Homeobox-like protein;(source:Araport11)	AT1G74220.1	-
Glyma.04g162300	Gm04	40236769-40236930	37010886_T_C37078558_G_A	Peak 2	R8/RL	88.50	Unknown protein	-	-
Glyma.04g162400	Gm04	40236981-40239376	37078558_G_A	Peak 2	R8/RL	95.92	Exocyst subunit exo70 family protein E2	AT5G61010.1	-
Glyma.04g162500	Gm04	40265908-40266378	37078558_G_A	Peak 2	R8/RL	94.01	Cytochrome oxidase 2	ATMG00160.1	-
Glyma.04g162600	Gm04	40278029-40282430	37010886_T_C37078558_G_A	Peak 2	R8/RL	89.00	Uncharacterized protein LOC100792679 isoform X1 [Glycine max]	AT5G08010.1	-
Glyma.04g162700	Gm04	40326865-40331483	37078558_G_A	Peak 2	R8/RL	91.07	3-phosphoserine phosphatase	AT1G18640.2	-
Glyma.04g162800	Gm04	40387251-40393343	37078558_G_A	Peak 2	R8/RL	91.00	Gamma subunit of Mt ATP synthase	AT2G33040.1	-
Glyma.04g162900	Gm04	40388840-40389153	37078558_G_A	Peak 2	R8/RL	93.20	Unknown protein	-	-
Glyma.04g163000	Gm04	40423906-40429027	37010886_T_C37078558_G_A	Peak 2	R8/RL	92.10	Mitochondrial substrate carrier family protein	AT1G74240.1	-

Glyma.04g163100	Gm04	40431122-40438954	37078558_G_A	Peak 2	R8/RL	92.98	PHD finger-containing protein. Interacts with BDT1, acts with other PHD proteins to associate with flowering genes and thereby suppress their transcription.	AT5G61120.1	-
Glyma.04g163200	Gm04	40464589-40467748	37078558_G_A	Peak 2	R8/RL	88.90	Heat shock protein 101	AT1G74310.1	-
Glyma.04g163300	Gm04	40483595-40484853	37078558_G_A	Peak 2	R8/RL	89.95	Acyl-CoA N-acyltransferases (NAT) superfamily protein	AT4G19985.1	-
Glyma.04g163600	Gm04	40582252-40596618	37078558_G_A	Peak 2	R8/RL	89.83	Dentin sialophosphoprotein-like protein	AT5G07940.1	N
Glyma.04g163900	Gm04	40730419-40731291	37078558_G_A	Peak 2	R8/RL	89.60	Serine carboxypeptidase-like 46)	AT2G33530.1	-
Glyma.04g164000	Gm04	40803633-40804670	37078558_G_A	Peak 2	R8/RL	89.90	Myb/SANT-like DNA-binding domain protein;(source:Araport11)	AT5G05800.1	-
Glyma.04g164100	Gm04	40811977-40820483	37078558_G_A	Peak 2	R8/RL	89.85	Leucine-rich repeat (LRR) family protein	AT5G07910.1	-
Glyma.04g164400	Gm04	40973232..40975775	37010886_T_C37078558_G_A	Peak 2	R8/RL	86.50	Soluble N-ethylmaleimide-sensitive factor adaptor protein 33	AT5G61210.1	-
Glyma.04g164500	Gm04	41027892-41033697	37010886_T_C37078558_G_A37126858_A_G	Peak 2	R8/RL	87.70	Glucuronidase 2	AT5G07830.1	-
Glyma.04g164600	Gm04	41086167-41089544	37010886_T_C37126858_A_G	Peak 2	R8/RL	88.41	Varicose-related	AT3G13290.1	-
Glyma.04g164700	Gm04	41098678-41099094	37010886_T_C37126858_A_G	Peak 2	R8/RL	86.11	Protein of unknown function (DUF1278))	AT1G76750.1	-
Glyma.04g164800	Gm04	41104671-41105875	37126858_A_G	Peak 2	R8/RL	84.51	Remorin family protein	AT5G61280.1	-
Glyma.04g165400	Gm04	41326473-41330652	37126858_A_G	Peak 2	R8/RL	85.78	Leucine-rich repeat protein kinase family protein	AT1G74360.1	-
Glyma.10g180000	Gm10	41336339-41337449	40908884_G_A	Peak 3	R1	85.92	AUX/IAA transcriptional regulator family protein)	AT1G04240.1	-
Glyma.10g180200	Gm10	41387259-41392378	40908884_G_A	Peak 3	R1	85.99	Magnesium transporter 9	AT5G64560.1	-
Glyma.10g180300	Gm10	41398806-41402748	40908884_G_A	Peak 3	R1	86.70	DREB2A-interacting protein 2	AT2G30580.1	-
Glyma.10g180600	Gm10	41417050-41421842	40908884_G_A	Peak 3	R1	85.50	Cryptochrome 2	AT1G04400.1	DQ401047.1 / AB498935.1/AB498936.1/

Glyma.10g180800	Gm10	41440863-41443235	40908884_G_A	Peak 3	R1	93.29	Myb domain protein 15	AT3G23250.1	-
Glyma.10g180900	Gm10	41454466-41454978	40908884_G_A	Peak 3	R1	90.65	Unknown protein	AT4G14380.1	-
Glyma.10g181000	Gm10	41457399-41458480	40908884_G_A	Peak 3	R1	86.11	Unknown protein	AT4G14380.1	-
Glyma.10g181100	Gm10	41462028-41467447	40908884_G_A	Peak 3	R1	86.38	XB3 ortholog 5 in Arabidopsis thaliana	AT3G23280.2	-
Glyma.10g181600	Gm10	41499182-41500142	40908884_G_A	Peak 3	R1	96.40	Unknown protein		-
Glyma.10g181700	Gm10	41505696-41512460	40908884_G_A	Peak 3	R1	86.17	Casein kinase I	AT4G14340.1	-
Glyma.10g182300	Gm10	41561515-41570949	40908884_G_A	Peak 3	R1	89.08	COP1-interacting protein-related	AT5G43310.3	-
Glyma.10g182400	Gm10	41572844-41575499	40908884_G_A	Peak 3	R1	89.22	Protein phosphatase 2C family protein	AT3G23360.1	-

Chr is chromosome, Gen Pos is gene positions in Wm82.a1.v1 genome assembly, R8 is days to maturity, RL is reproductive length, R1 days to flowering, [a]Tagging SNPs IDs start with “BARC_1.01_Gm” and the number of chromosome and the positions are based on Wm82.a1; [b] Average accuracies obtained by AccuTool. [d] Orthologs genes name in Arabidopsis. [e] Gene bank ID from reference sequences (RefSeqs) of the gene in *Glycine max.* available at NCBI website
