# Supplementary material for: Genome-wide association study reveals GmFulb as candidate gene for maturity time and reproductive length in soybeans (Glycine max)
Source: PLoS One. 2024 Jan 19;19(1):e0294123. doi: 10.1371/journal.pone.0294123 (PMC10798547; doi:10.1371/journal.pone.0294123)
Supplement: S9 Table — (PDF) [file pone.0294123.s017.pdf]

S9 Table. Correspondence between tagging SNPs and known E genes measured by average accuracy obtained from AcuTool.

Gene	Pos. (bp)[a]	Chr	Average Accuracy						
			Gm10 41455680[a]	Gm04 17228343[a]	Gm04 17075267[a]	Gm04 40009617[a]	Gm04 40276263[a]	Gm04 40151473[a]	Gm04 40218961[a]
**E1La**	36,758,125<br>36,758,770	4		78.2	79.3	73	83	83	83
**E1Lb**	26,120,011<br>26,120,532	4		50	30	50	50	50	50
**E2 /Glyma.10g221500**	45,294,735<br>45,316,121	10	43.4						
**E8**	9,337,214<br>9,341,731	4		73	73	70	71	66	65

[a] Gene and SNP positions are based on Wm82.a2.v1 genome assembly.
