# Supplementary material for: Genome-wide association study reveals GmFulb as candidate gene for maturity time and reproductive length in soybeans (Glycine max)
Source: PLoS One. 2024 Jan 19;19(1):e0294123. doi: 10.1371/journal.pone.0294123 (PMC10798547; doi:10.1371/journal.pone.0294123)
Supplement: S1 File — (DOCX) [file pone.0294123.s018.docx]

Supplementary Material

Table S1. Planting dates of experimental environments.

| Environment^a^ | Location | year | Planting date |
| --- | --- | --- | --- |
| E1 | Columbia- MO | 2017 | 16-May |
| E2 | Columbia- MO | 2018 | 15-May |
| E3 | ACRE-IN | 2017 | 18-May |
| E4 | ACRE-IN | 2018 | 22-May |
| E5 | ACRE-IN | 2019 | 4-Jun |
| E6 | ACRE-IN | 2020 | 24-May |
| E7 | Romney-IN | 2019 | 8-Jun |
| E8 | Romney-IN | 2020 | 27-May |
| E9 | Gibson-Il | 2020 | 23-Apr |

^a^ Individual environments represent the combination of location and year.

Table S2. Mean flowering (R1), and maturity (R8) time in days after planting (DAP) and mean reproductive length (RL) in number of days per accessions across all environments.

| Accession | R1 | | R8 | | RL | | Accession | R1 | | R8 | | RL | |
| --- | --- | --- | --- | --- | --- | --- | --- | --- | --- | --- | --- | --- | --- |
|  | mean | SD | mean | SD | mean | SD |  | mean | SD | mean | SD | mean | SD |
| PI153243 | 50.5 | 7.7 | 127.2 | 12.8 | 76.6 | 7.7 | **PI548543** | 4.6 | 128.7 | 5.5 | 88.3 | 5.5 | 4.6 |
| PI153292 | 45.2 | 8.0 | 127.2 | 13.0 | 81.9 | 9.0 | **PI548546** | 9.0 | 131.9 | 15.1 | 80.1 | 8.5 | 9.0 |
| PI170380 | 50.2 | 7.9 | 125.8 | 13.2 | 75.6 | 6.8 | **PI548547** | 6.8 | 131.1 | 13.1 | 81.9 | 8.4 | 6.8 |
| PI189926 | 49.2 | 7.9 | 130.2 | 15.6 | 81.0 | 9.8 | **PI548549** | 8.4 | 133.7 | 14.8 | 84.9 | 9.2 | 8.4 |
| PI189928 | 47.1 | 6.2 | 125.5 | 15.7 | 78.4 | 10.0 | **PI548555** | 9.0 | 138.1 | 14.2 | 89.9 | 6.8 | 9.0 |
| PI189935 | 50.6 | 8.4 | 132.9 | 15.8 | 82.3 | 8.1 | **PI548558** | 6.4 | 126.7 | 12.3 | 79.3 | 7.6 | 6.4 |
| PI209331 | 46.0 | 6.5 | 126.7 | 13.6 | 80.7 | 8.3 | **PI548559** | NA | 133.0 | NA | 83.0 | NA | NA |
| PI248402 | 52.9 | 9.5 | 136.2 | 15.0 | 83.4 | 7.6 | **PI548562** | 6.7 | 122.8 | 12.0 | 76.8 | 6.4 | 6.7 |
| PI253660A | 47.5 | 5.3 | 126.3 | 12.8 | 78.9 | 8.5 | **PI548574** | 10.6 | 119.5 | 3.5 | 76.0 | 7.1 | 10.6 |
| PI253661B | 53.0 | 10.1 | 122.5 | 12.4 | 69.5 | 4.4 | **PI548585** | 7.4 | 129.4 | 12.4 | 82.6 | 8.0 | 7.4 |
| PI283331 | 50.7 | 8.3 | 127.8 | 13.5 | 77.1 | 7.2 | **PI548597** | 7.1 | 122.2 | 12.3 | 77.5 | 8.0 | 7.1 |
| PI370055 | 47.4 | 7.0 | 125.7 | 14.0 | 78.4 | 8.2 | **PI548598** | 8.3 | 137.1 | 15.5 | 87.1 | 9.9 | 8.3 |
| PI371611 | 49.4 | 7.1 | 134.0 | 14.3 | 84.6 | 9.3 | **PI548602** | 11.0 | 138.3 | 15.8 | 84.9 | 7.8 | 11.0 |
| PI398881 | 48.3 | 6.9 | 122.9 | 11.3 | 74.7 | 5.8 | **PI548603** | 10.1 | 136.1 | 14.7 | 82.6 | 6.5 | 10.1 |
| PI404173B | 55.9 | 11.1 | 134.6 | 15.6 | 78.7 | 7.1 | **PI548613** | 6.4 | 128.7 | 14.9 | 83.0 | 9.8 | 6.4 |
| PI417526 | 48.5 | 6.7 | 127.7 | 14.5 | 79.2 | 8.8 | **PI548614** | 8.1 | 126.8 | 14.4 | 79.2 | 8.1 | 8.1 |
| PI417573 | 50.5 | 8.7 | 130.5 | 14.2 | 80.0 | 8.5 | **PI548622** | 6.4 | 124.5 | 0.7 | 84.0 | 5.7 | 6.4 |
| PI424159B | 48.3 | 7.5 | 135.8 | 13.6 | 87.5 | 7.5 | **PI548632** | 7.3 | 126.1 | 12.0 | 78.6 | 7.4 | 7.3 |
| PI424405A | 46.2 | 7.2 | 128.7 | 11.5 | 82.5 | 6.4 | **PI548633** | 10.1 | 128.4 | 12.7 | 78.6 | 6.7 | 10.1 |
| PI424405B | 50.3 | 7.4 | 136.0 | 13.5 | 85.7 | 7.8 | **PI548634** | 9.9 | 121.5 | 3.5 | 78.5 | 6.4 | 9.9 |
| PI427136 | 51.4 | 8.7 | 129.7 | 14.1 | 77.7 | 6.2 | **PI548635** | 8.7 | 127.0 | 10.8 | 78.3 | 3.3 | 8.7 |
| PI432359 | 49.4 | 7.6 | 135.4 | 14.8 | 86.0 | 9.2 | **PI548636** | 10.3 | 134.9 | 14.5 | 85.3 | 6.2 | 10.3 |
| PI437103 | 51.1 | 7.0 | 123.0 | 11.6 | 71.9 | 6.4 | **PI548652** | 7.5 | 129.4 | 11.8 | 83.4 | 6.1 | 7.5 |
| PI437124 | 47.2 | 6.6 | 123.6 | 12.0 | 76.5 | 6.8 | **PI548670** | 10.1 | 131.5 | 16.2 | 82.4 | 9.0 | 10.1 |
| PI437317 | 46.3 | 7.7 | 125.0 | 15.6 | 78.7 | 9.4 | **PI548671** | 6.3 | 134.5 | 16.2 | 79.5 | 10.7 | 6.3 |
| PI437338 | 48.8 | 6.7 | 129.1 | 13.2 | 80.3 | 8.8 | **PI548678** | 9.6 | 138.1 | 16.4 | 81.0 | 8.7 | 9.6 |
| PI437357 | 49.9 | 7.3 | 124.7 | 12.3 | 74.8 | 6.0 | **PI548679** | 10.1 | 138.2 | 15.4 | 85.5 | 8.9 | 10.1 |
| PI437359 | 50.5 | 9.2 | 129.6 | 12.4 | 79.1 | 6.3 | **PI548682** | 7.1 | 134.0 | 14.6 | 86.1 | 9.8 | 7.1 |
| PI437365 | 49.7 | 8.2 | 125.3 | 13.5 | 75.7 | 6.8 | **PI548684** | 9.8 | 126.3 | 14.8 | 77.6 | 7.5 | 9.8 |
| PI437367 | 50.2 | 7.9 | 134.2 | 15.0 | 84.0 | 8.4 | **PI548685** | 6.7 | 127.3 | 13.0 | 81.5 | 7.6 | 6.7 |
| PI437377 | 51.5 | 8.2 | 123.1 | 12.2 | 71.6 | 5.2 | **PI548686** | 9.2 | 124.7 | 14.4 | 75.7 | 6.4 | 9.2 |
| PI437388 | 51.3 | 7.4 | 130.5 | 17.1 | 79.2 | 12.6 | **PI552538** | 8.4 | 124.6 | 11.0 | 79.6 | 7.7 | 8.4 |
| PI437391 | 47.2 | 8.2 | 129.3 | 13.4 | 82.1 | 7.7 | **PI555396** | 9.5 | 134.1 | 14.8 | 85.4 | 7.4 | 9.5 |
| PI437392 | 47.0 | 6.9 | 125.0 | 11.9 | 78.0 | 7.3 | **PI555399** | 8.5 | 139.9 | 14.6 | 85.5 | 8.4 | 8.5 |
| PI437397 | 50.0 | 10.0 | 127.8 | 16.4 | 77.8 | 7.8 | **PI556511** | 7.3 | 124.9 | 12.6 | 81.2 | 8.5 | 7.3 |
| PI437412 | 47.8 | 7.8 | 121.8 | 12.7 | 74.0 | 5.7 | **PI556572** | 8.6 | 133.3 | 14.3 | 86.7 | 7.9 | 8.6 |
| PI437422 | 49.8 | 6.1 | 125.2 | 11.9 | 75.3 | 8.4 | **PI556687** | 6.8 | 130.0 | 12.8 | 83.7 | 8.0 | 6.8 |
| PI437444 | 49.1 | 7.3 | 129.0 | 14.2 | 79.9 | 7.8 | **PI556778** | 10.4 | 129.4 | 11.8 | 81.8 | 5.8 | 10.4 |
| PI437459 | 48.1 | 7.9 | 129.9 | 12.5 | 80.7 | 9.2 | **PI556780** | 7.9 | 135.7 | 13.9 | 88.4 | 8.3 | 7.9 |
| PI437461 | 50.9 | 8.4 | 127.0 | 12.9 | 76.1 | 6.9 | **PI556781** | 8.5 | 129.3 | 13.0 | 82.8 | 8.4 | 8.5 |
| PI437463B | 45.5 | 5.9 | 121.7 | 12.0 | 76.2 | 7.1 | **PI556814** | 10.0 | 135.0 | 14.1 | 87.9 | 7.6 | 10.0 |
| PI437464 | 48.2 | 7.0 | 131.8 | 15.7 | 83.6 | 9.4 | **PI556816** | 9.4 | 129.8 | 12.6 | 82.5 | 6.4 | 9.4 |
| PI437467 | 48.8 | 8.4 | 126.8 | 10.7 | 77.9 | 5.0 | **PI556857** | 8.1 | 135.1 | 14.1 | 88.6 | 7.8 | 8.1 |
| PI437487 | 49.6 | 7.8 | 130.6 | 14.9 | 81.0 | 9.5 | **PI556859** | 8.9 | 131.5 | 13.9 | 84.8 | 7.0 | 8.9 |
| PI437500B | 46.3 | 7.9 | 126.8 | 11.8 | 80.5 | 6.7 | **PI556889** | 10.5 | 139.5 | 14.6 | 89.1 | 5.9 | 10.5 |
| PI437500C | 45.6 | 7.3 | 122.3 | 11.8 | 76.7 | 6.7 | **PI556928** | 8.6 | 131.7 | 13.7 | 83.2 | 7.1 | 8.6 |
| PI437550C | 48.8 | 8.3 | 124.6 | 11.1 | 75.9 | 4.8 | **PI556931** | 7.3 | 126.1 | 10.8 | 81.9 | 6.8 | 7.3 |
| PI437641B | 47.6 | 7.2 | 122.8 | 12.9 | 75.3 | 7.3 | **PI556932** | 10.0 | 137.1 | 13.3 | 84.7 | 6.4 | 10.0 |
| PI437711B | 51.8 | 8.6 | 133.5 | 17.1 | 81.7 | 10.0 | **PI556989** | 7.3 | 124.7 | 10.2 | 78.7 | 5.0 | 7.3 |
| PI437788B | 54.0 | NA | 133.0 | NA | 79.0 | NA | **PI559931** | 7.3 | 128.3 | 5.3 | 85.8 | 6.4 | 7.3 |
| PI437789 | 55.0 | NA | 133.0 | NA | 78.0 | NA | **PI560206** | 7.4 | 137.8 | 14.3 | 88.3 | 7.7 | 7.4 |
| PI437790 | 53.0 | NA | 131.0 | NA | 78.0 | NA | **PI560207** | 7.7 | 137.4 | 16.3 | 85.1 | 10.3 | 7.7 |
| PI437877C | 43.0 | NA | 128.0 | NA | 85.0 | NA | **PI561321** | 6.8 | 125.2 | 15.3 | 79.7 | 9.5 | 6.8 |
| PI437888B | 51.0 | NA | 121.0 | NA | 70.0 | NA | **PI561330A** | 6.1 | 122.1 | 12.4 | 75.4 | 6.7 | 6.1 |
| PI437894 | 47.6 | 6.5 | 128.3 | 13.4 | 80.7 | 7.8 | **PI564718** | 9.0 | 126.5 | 13.1 | 77.6 | 6.6 | 9.0 |
| PI437903 | 52.0 | 9.7 | 125.5 | 11.3 | 73.5 | 5.1 | **PI567250B** | NA | 132.0 | NA | 79.0 | NA | NA |
| PI438073 | 54.0 | NA | 133.0 | NA | 79.0 | NA | **PI567351B** | 9.0 | 135.5 | 16.6 | 83.0 | 9.2 | 9.0 |
| PI438079 | 50.9 | 9.1 | 127.1 | 13.1 | 76.2 | 5.0 | **PI567366A** | 9.4 | 128.8 | 11.5 | 75.5 | 7.8 | 9.4 |
| PI438124B | 48.8 | 6.8 | 125.5 | 11.5 | 76.7 | 6.6 | **PI567404A** | 9.1 | 129.8 | 14.7 | 78.1 | 8.5 | 9.1 |
| PI438252C | 49.5 | 9.1 | 128.2 | 12.2 | 78.7 | 6.2 | **PI567527** | 9.4 | 137.8 | 14.4 | 85.7 | 6.1 | 9.4 |
| PI438303 | 55.0 | NA | 132.0 | NA | 77.0 | NA | **PI567767B** | 8.1 | 133.0 | 12.3 | 85.1 | 5.8 | 8.1 |
| PI438310 | 51.6 | 8.1 | 123.9 | 11.7 | 72.3 | 6.3 | **PI568245** | 7.2 | 132.1 | 12.9 | 84.9 | 8.7 | 7.2 |
| PI438312 | 62.0 | NA | 123.0 | NA | 61.0 | NA | **PI574532** | 8.3 | 125.4 | 11.7 | 79.1 | 6.2 | 8.3 |
| PI438341 | 44.5 | 8.2 | 123.5 | 13.1 | 78.9 | 8.3 | **PI574534** | 7.4 | 128.6 | 13.0 | 84.1 | 7.2 | 7.4 |
| PI438357B | 50.5 | 8.2 | 133.6 | 15.5 | 83.1 | 9.3 | **PI574541** | 6.8 | 133.5 | 14.8 | 84.5 | 8.7 | 6.8 |
| PI438409 | 49.1 | 8.3 | 123.0 | 12.5 | 73.9 | 10.0 | **PI576160** | 7.3 | 127.7 | 14.6 | 81.8 | 9.1 | 7.3 |
| PI438486 | 50.6 | 7.6 | 125.9 | 12.1 | 75.4 | 7.1 | **PI576166** | 10.0 | 136.3 | 12.9 | 88.5 | 6.3 | 10.0 |
| PI438495 | 59.0 | NA | 129.0 | NA | 70.0 | NA | **PI577798** | 9.6 | 138.1 | 15.1 | 87.8 | 7.5 | 9.6 |
| PI438500 | 47.3 | 8.1 | 129.1 | 10.6 | 81.8 | 6.2 | **PI578057** | 8.4 | 133.2 | 16.2 | 83.2 | 9.2 | 8.4 |
| PI475822B | 48.4 | 7.9 | 125.0 | 11.2 | 76.6 | 7.1 | **PI584441** | 7.7 | 130.2 | 14.5 | 82.1 | 8.0 | 7.7 |
| PI475822C | 56.6 | 11.4 | 136.5 | 18.1 | 79.8 | 9.8 | **PI584470** | 6.1 | 121.0 | 2.6 | 79.0 | 5.6 | 6.1 |
| PI479740 | 45.9 | 7.5 | 124.6 | 10.8 | 78.7 | 6.8 | **PI586980** | 7.4 | 123.6 | 13.1 | 78.1 | 7.6 | 7.4 |
| PI507676 | 47.8 | 8.1 | 123.3 | 12.8 | 75.5 | 7.4 | **PI590932** | 8.5 | 138.1 | 16.2 | 88.6 | 9.5 | 8.5 |
| PI507692B | 49.8 | 8.7 | 124.4 | 12.0 | 74.5 | 5.0 | **PI591490** | 7.4 | 138.1 | 17.0 | 87.8 | 12.0 | 7.4 |
| PI507696C | 49.4 | 8.7 | 144.3 | 16.6 | 94.9 | 8.7 | **PI591492** | 5.9 | 132.6 | 14.7 | 84.6 | 10.3 | 5.9 |
| PI507708 | 47.3 | 7.3 | 124.9 | 12.4 | 77.6 | 5.7 | **PI591493** | 8.4 | 133.9 | 15.6 | 83.2 | 9.5 | 8.4 |
| PI515961 | 50.5 | 9.0 | 141.4 | 14.4 | 90.9 | 7.2 | **PI591494** | 8.4 | 133.8 | 15.2 | 83.5 | 9.1 | 8.4 |
| PI518663 | 46.9 | 7.3 | 129.1 | 14.4 | 82.2 | 8.9 | **PI591495** | 7.1 | 136.9 | 14.7 | 84.3 | 9.3 | 7.1 |
| PI518667 | 46.0 | NA | 134.0 | NA | 88.0 | NA | **PI591497** | 7.1 | 132.1 | 11.7 | 85.1 | 6.4 | 7.1 |
| PI518668 | 51.6 | 8.1 | 139.4 | 15.7 | 87.8 | 10.1 | **PI591498** | 7.3 | 130.6 | 13.3 | 82.2 | 8.1 | 7.3 |
| PI518671 | 40.9 | 3.4 | 132.1 | 4.4 | 91.2 | 5.1 | **PI591499** | 7.4 | 132.8 | 15.2 | 83.7 | 9.5 | 7.4 |
| PI518673 | 49.3 | 10.0 | 131.2 | 11.3 | 81.9 | 6.3 | **PI591503** | 8.1 | 129.9 | 11.4 | 82.5 | 6.6 | 8.1 |
| PI518675 | 44.4 | 7.2 | 124.4 | 10.6 | 80.0 | 7.4 | **PI591504** | 9.2 | 130.3 | 14.2 | 82.1 | 6.4 | 9.2 |
| PI525454 | 50.0 | 10.7 | 134.0 | 16.3 | 84.0 | 7.2 | **PI591505** | 8.3 | 131.1 | 14.9 | 82.7 | 8.5 | 8.3 |
| PI533654 | 49.8 | 8.0 | 131.9 | 12.9 | 82.1 | 7.2 | **PI591506** | 6.3 | 127.8 | 9.8 | 82.6 | 6.9 | 6.3 |
| PI534645 | 44.4 | 8.0 | 124.8 | 10.2 | 81.6 | 7.1 | **PI591507** | 7.4 | 131.5 | 14.1 | 83.6 | 8.5 | 7.4 |
| PI534646 | 46.4 | 7.2 | 131.9 | 15.3 | 85.5 | 10.1 | **PI591509** | 8.8 | 130.6 | 13.7 | 83.6 | 7.8 | 8.8 |
| PI534647 | 45.8 | 8.0 | 126.2 | 10.2 | 80.4 | 6.4 | **PI591510** | 7.4 | 128.8 | 14.4 | 81.4 | 8.6 | 7.4 |
| PI534648 | 44.1 | 7.3 | 129.5 | 11.9 | 85.4 | 7.7 | **PI591512** | 8.1 | 128.1 | 11.9 | 80.9 | 5.7 | 8.1 |
| PI538401B | 49.3 | 7.0 | 135.1 | 14.9 | 85.8 | 9.2 | **PI591513** | 8.7 | 131.6 | 14.0 | 84.0 | 7.0 | 8.7 |
| PI540555 | 46.5 | 7.7 | 131.4 | 12.0 | 84.9 | 7.6 | **PI591515** | 7.7 | 129.5 | 14.2 | 84.5 | 9.3 | 7.7 |
| PI542043 | 47.8 | 8.6 | 131.8 | 13.3 | 85.4 | 8.4 | **PI591516** | 8.8 | 132.5 | 15.3 | 84.6 | 8.2 | 8.8 |
| PI542044 | 47.6 | 7.9 | 128.7 | 11.5 | 81.1 | 6.3 | **PI591517** | 8.8 | 129.8 | 13.5 | 82.1 | 7.2 | 8.8 |
| PI542709 | 47.2 | 8.1 | 127.1 | 11.2 | 79.9 | 6.1 | **PI591518** | 7.9 | 132.4 | 15.5 | 85.1 | 8.9 | 7.9 |
| PI542711 | 46.2 | 7.0 | 130.7 | 11.9 | 84.5 | 8.2 | **PI591519** | 8.0 | 130.7 | 14.0 | 83.9 | 7.3 | 8.0 |
| PI543793 | 48.3 | 7.8 | 137.9 | 13.6 | 89.7 | 9.0 | **PI591521** | 8.9 | 132.5 | 15.2 | 84.1 | 7.9 | 8.9 |
| PI547471 | 52.5 | 8.6 | 136.1 | 16.4 | 83.6 | 9.4 | **PI591526** | 8.6 | 130.6 | 14.6 | 82.8 | 8.1 | 8.6 |
| PI547477 | 49.5 | 6.8 | 135.0 | 14.6 | 85.5 | 9.4 | **PI591528** | 7.6 | 131.1 | 13.4 | 83.1 | 8.1 | 7.6 |
| PI547484 | 47.7 | 7.3 | 128.1 | 13.2 | 80.4 | 7.8 | **PI591530** | 7.8 | 132.2 | 14.2 | 84.4 | 7.9 | 7.8 |
| PI547497 | 48.8 | 5.8 | 133.5 | 15.2 | 84.8 | 10.6 | **PI591531** | 7.5 | 128.7 | 13.5 | 80.3 | 7.0 | 7.5 |
| PI547500 | 49.3 | 8.7 | 128.8 | 13.1 | 79.5 | 8.3 | **PI591532** | 8.9 | 131.0 | 13.8 | 81.4 | 9.2 | 8.9 |
| PI547501 | 45.6 | 6.7 | 127.7 | 14.3 | 82.1 | 8.2 | **PI591533** | 7.6 | 128.1 | 11.8 | 81.8 | 6.8 | 7.6 |
| PI547545 | 49.8 | 6.8 | 134.1 | 13.4 | 84.3 | 8.5 | **PI591534** | 6.7 | 124.1 | 10.9 | 79.0 | 6.0 | 6.7 |
| PI547598 | 48.9 | 6.7 | 133.6 | 13.4 | 84.7 | 9.2 | **PI591535** | 7.2 | 130.7 | 14.3 | 82.1 | 7.9 | 7.2 |
| PI547617 | 50.3 | 9.4 | 133.7 | 14.5 | 83.4 | 8.0 | **PI591536** | 8.4 | 135.1 | 15.5 | 86.1 | 8.7 | 8.4 |
| PI547655 | 48.7 | 6.5 | 131.4 | 10.7 | 82.7 | 6.4 | **PI591537** | 7.4 | 133.6 | 15.5 | 86.9 | 10.4 | 7.4 |
| PI547657 | 48.9 | 8.3 | 132.5 | 14.2 | 83.6 | 7.7 | **PI591538** | 7.4 | 129.3 | 14.1 | 82.8 | 8.6 | 7.4 |
| PI547663 | 47.5 | 6.8 | 126.7 | 10.4 | 79.2 | 7.0 | **PI591539** | 7.4 | 130.1 | 14.6 | 82.4 | 8.3 | 7.4 |
| PI547798 | 47.0 | 7.3 | 125.9 | 12.3 | 78.9 | 6.7 | **PI591540** | 7.5 | 126.9 | 11.9 | 79.8 | 7.4 | 7.5 |
| PI547800 | 49.0 | 8.6 | 125.9 | 10.8 | 76.9 | 4.7 | **PI591561** | 8.9 | 131.8 | 15.7 | 85.7 | 10.4 | 8.9 |
| PI547801 | 49.8 | 6.1 | 128.0 | 12.1 | 78.2 | 9.3 | **PI592946** | 9.9 | 129.9 | 15.0 | 78.9 | 7.1 | 9.9 |
| PI547802 | 49.2 | 7.6 | 129.4 | 13.8 | 80.2 | 7.8 | **PI593256** | 9.2 | 134.9 | 14.6 | 86.2 | 6.8 | 9.2 |
| PI547811 | 46.1 | 7.6 | 123.5 | 10.6 | 78.5 | 8.2 | **PI593258** | 7.8 | 130.3 | 13.3 | 85.3 | 7.2 | 7.8 |
| PI547813 | 47.9 | 7.8 | 127.5 | 15.6 | 79.6 | 9.0 | **PI593463** | 8.0 | 127.6 | 11.4 | 80.8 | 5.2 | 8.0 |
| PI547814 | 46.5 | 7.4 | 124.8 | 11.3 | 78.3 | 6.5 | **PI593654** | 6.5 | 126.9 | 11.6 | 81.6 | 7.6 | 6.5 |
| PI547847 | 49.2 | 8.1 | 131.3 | 14.2 | 82.1 | 7.4 | **PI595363** | 7.9 | 128.0 | 14.6 | 83.1 | 9.6 | 7.9 |
| PI547860 | 47.1 | 7.7 | 131.3 | 14.5 | 84.2 | 8.8 | **PI595754** | 7.3 | 128.1 | 12.1 | 80.5 | 6.9 | 7.3 |
| PI547864 | 46.8 | 8.5 | 131.1 | 14.1 | 84.3 | 7.7 | **PI595926** | 7.5 | 122.7 | 11.2 | 77.4 | 6.7 | 7.5 |
| PI547869 | 48.1 | 7.6 | 129.6 | 12.2 | 81.5 | 6.8 | **PI596407** | 7.4 | 125.2 | 10.5 | 77.3 | 5.8 | 7.4 |
| PI547876 | 48.0 | 8.7 | 133.7 | 14.3 | 85.7 | 7.9 | **PI597382** | 8.0 | 133.6 | 13.4 | 87.4 | 7.0 | 8.0 |
| PI547879 | 50.9 | 9.5 | 132.9 | 13.6 | 82.0 | 6.0 | **PI597387** | 7.5 | 132.1 | 14.6 | 86.6 | 8.5 | 7.5 |
| PI547885 | 48.1 | 8.3 | 130.3 | 14.6 | 82.1 | 7.6 | **PI598124** | 5.7 | 126.0 | 1.4 | 91.0 | 4.2 | 5.7 |
| PI547886 | 47.8 | 7.5 | 130.9 | 14.3 | 83.1 | 8.0 | **PI602449** | 9.2 | 133.0 | 12.8 | 82.3 | 5.7 | 9.2 |
| PI548174 | 48.5 | 6.6 | 129.1 | 14.3 | 80.5 | 8.1 | **PI602450** | 7.8 | 133.3 | 12.8 | 81.8 | 7.6 | 7.8 |
| PI548177 | 50.6 | 7.6 | 130.2 | 12.0 | 79.6 | 7.0 | **PI603186** | 7.2 | 130.4 | 13.6 | 78.9 | 9.7 | 7.2 |
| PI548180 | 48.5 | 6.6 | 123.3 | 12.0 | 74.8 | 6.4 | **PI603188** | 7.8 | 129.7 | 12.0 | 80.1 | 7.1 | 7.8 |
| PI548191 | 52.5 | 9.7 | 134.2 | 16.6 | 81.6 | 8.5 | **PI603193** | 6.9 | 131.4 | 12.4 | 81.9 | 8.2 | 6.9 |
| PI548192 | 50.7 | 6.4 | 135.4 | 14.1 | 84.7 | 9.2 | **PI603201** | 8.8 | 138.0 | 16.7 | 80.2 | 9.9 | 8.8 |
| PI548193 | 51.8 | 9.2 | 135.6 | 15.8 | 83.5 | 7.6 | **PI603205** | 7.5 | 134.2 | 12.8 | 82.6 | 6.4 | 7.5 |
| PI548194 | 50.7 | 9.9 | 132.2 | 14.6 | 81.5 | 6.6 | **PI603396** | 8.0 | 127.5 | 14.3 | 74.1 | 7.3 | 8.0 |
| PI548198 | 55.0 | 8.5 | 119.5 | 3.5 | 64.5 | 12.0 | **PI603428D** | 6.5 | 128.1 | 11.5 | 79.2 | 7.0 | 6.5 |
| PI548204 | 50.0 | 5.6 | 124.5 | 14.5 | 74.5 | 10.5 | **PI603434** | 7.0 | 124.2 | 11.1 | 76.7 | 5.4 | 7.0 |
| PI548222 | 49.9 | 10.2 | 136.8 | 14.9 | 86.9 | 7.5 | **PI603442** | 7.7 | 122.4 | 12.5 | 77.9 | 6.0 | 7.7 |
| PI548231 | 45.9 | 6.4 | 127.6 | 11.4 | 81.7 | 6.9 | **PI603454** | 8.0 | 132.4 | 13.6 | 83.6 | 7.7 | 8.0 |
| PI548242 | 47.0 | 8.2 | 128.5 | 13.4 | 81.4 | 7.3 | **PI603564A** | 6.2 | 133.0 | 12.1 | 82.8 | 9.6 | 6.2 |
| PI548247 | 49.7 | 7.4 | 131.6 | 13.0 | 81.9 | 7.2 | **PI603564B** | 8.2 | 136.8 | 14.6 | 82.9 | 8.0 | 8.2 |
| PI548250 | 55.1 | 11.1 | 139.8 | 14.4 | 84.7 | 6.0 | **PI603564C** | 7.4 | 137.4 | 15.7 | 85.9 | 10.6 | 7.4 |
| PI548253 | 47.3 | 8.0 | 132.1 | 14.2 | 84.8 | 8.2 | **PI603571B** | 9.5 | 133.6 | 12.7 | 85.6 | 7.0 | 9.5 |
| PI548260 | 54.3 | 5.5 | 131.0 | 3.6 | 76.7 | 7.5 | **PI603915D** | 8.8 | 133.5 | 13.7 | 85.6 | 6.5 | 8.8 |
| PI548262 | 46.5 | 8.7 | 131.4 | 13.3 | 84.9 | 6.5 | **PI606748** | 8.2 | 135.8 | 13.5 | 89.4 | 8.1 | 8.2 |
| PI548267 | 52.1 | 7.0 | 131.7 | 11.8 | 79.7 | 7.8 | **PI606749** | 7.6 | 139.2 | 14.0 | 91.1 | 7.5 | 7.6 |
| PI548275 | 49.0 | 7.7 | 132.5 | 13.9 | 83.5 | 7.6 | **PI610670** | 7.2 | 126.5 | 12.3 | 79.0 | 6.3 | 7.2 |
| PI548286 | 47.7 | 9.3 | 132.2 | 13.2 | 84.5 | 7.0 | **PI612594** | 7.1 | 130.0 | 13.7 | 85.0 | 8.0 | 7.1 |
| PI548297 | 51.8 | 9.1 | 135.0 | 14.7 | 83.2 | 6.8 | **PI612750** | 7.4 | 124.3 | 13.1 | 80.3 | 8.0 | 7.4 |
| PI548304 | 46.2 | 6.4 | 123.8 | 13.2 | 77.5 | 7.7 | **PI612932** | 7.8 | 123.4 | 11.4 | 77.1 | 5.2 | 7.8 |
| PI548333 | 47.9 | 9.3 | 134.5 | 13.6 | 86.6 | 7.9 | **PI614153** | 7.2 | 130.8 | 12.5 | 86.6 | 7.8 | 7.2 |
| PI548339 | 52.2 | 8.7 | 133.7 | 16.0 | 81.5 | 10.5 | **PI614154** | 8.0 | 125.6 | 10.5 | 81.8 | 6.1 | 8.0 |
| PI548340 | 49.0 | 10.2 | 128.0 | 14.5 | 80.8 | 7.6 | **PI614155** | 8.2 | 134.6 | 12.0 | 88.1 | 6.7 | 8.2 |
| PI548365 | 46.8 | 8.1 | 126.2 | 16.2 | 79.3 | 8.9 | **PI614832** | 7.9 | 126.8 | 11.5 | 81.3 | 5.8 | 7.9 |
| PI548366 | 48.8 | 8.7 | 121.2 | 12.0 | 73.9 | 6.1 | **PI615555** | 7.7 | 127.7 | 12.1 | 80.8 | 6.2 | 7.7 |
| PI548367 | 48.6 | 8.9 | 127.4 | 13.1 | 78.8 | 6.9 | **PI615556** | 6.4 | 131.5 | 14.0 | 82.5 | 8.3 | 6.4 |
| PI548373 | 49.7 | 7.3 | 127.0 | 14.9 | 77.3 | 9.0 | **PI633729** | 7.8 | 131.2 | 14.0 | 83.9 | 7.3 | 7.8 |
| PI548381 | 46.7 | 7.7 | 122.1 | 12.1 | 75.4 | 6.0 | **PI633732** | 7.3 | 133.9 | 15.3 | 89.4 | 10.3 | 7.3 |
| PI548383 | 49.4 | 6.5 | 129.0 | 14.7 | 79.6 | 10.1 | **PI634761** | 8.2 | 128.1 | 13.4 | 79.9 | 8.1 | 8.2 |
| PI548400 | 52.7 | 10.1 | 135.7 | 15.2 | 83.0 | 6.1 | **PI634762** | 7.3 | 131.1 | 12.7 | 81.0 | 7.9 | 7.3 |
| PI548410 | 51.0 | 8.5 | 134.4 | 15.2 | 83.4 | 9.1 | **PI634763** | 8.8 | 134.4 | 13.4 | 83.6 | 7.1 | 8.8 |
| PI548412 | 47.3 | 8.2 | 130.8 | 14.4 | 83.5 | 7.7 | **PI634764** | 8.1 | 129.2 | 16.3 | 80.1 | 10.1 | 8.1 |
| PI548421 | 48.3 | 6.3 | 129.0 | 14.7 | 80.7 | 8.9 | **PI634765** | 7.5 | 130.4 | 14.2 | 81.5 | 8.5 | 7.5 |
| PI548517 | 49.8 | 11.5 | 133.5 | 17.1 | 83.7 | 9.5 | **PI636464** | 7.9 | 127.7 | 10.2 | 81.3 | 5.8 | 7.9 |
| PI548518 | 46.9 | 6.2 | 126.1 | 14.0 | 79.2 | 8.8 | **PI636695** | 7.0 | 135.0 | 14.9 | 84.7 | 11.2 | 7.0 |
| PI548522 | 43.8 | 4.6 | 123.5 | 7.5 | 79.8 | 10.7 | **PI636696** | 11.2 | 140.4 | 13.5 | 84.3 | 6.9 | 11.2 |
| PI548525 | 50.6 | 9.0 | 127.4 | 14.1 | 76.8 | 5.9 | **PI639740** | 10.6 | 130.5 | 0.7 | 86.0 | 9.9 | 10.6 |
| PI548532 | 49.7 | 7.8 | 130.3 | 13.0 | 80.7 | 7.4 | **PI642055** | 9.4 | 140.7 | 16.2 | 86.3 | 8.7 | 9.4 |
| PI548541 | 53.3 | 9.0 | 139.1 | 14.7 | 85.8 | 7.1 | **PI643146** | 8.5 | 128.5 | 0.7 | 86.5 | 9.2 | 8.5 |
| PI548542 | 46.4 | 7.5 | 126.3 | 11.6 | 79.9 | 6.2 |  |  |  |  |  |  |  |

NA is present when the accession had only one data point and the standard deviation (sd) could not be estimated

| Trait | Environment^a^ | Year | Location | min | max | mean | sd | CV | H entry (%) | h^2^ (%) |
| --- | --- | --- | --- | --- | --- | --- | --- | --- | --- | --- |
| R1 | All environments |  | All environments | 31 | 80 | 48.5 | 8.2 | 16.9 | 65 | 60 |
| R1 | E1 | 2017 | Columbia-MO | 39 | 62 | 49.6 | 4.1 | 8.2 |  |  |
| R1 | E2 | 2018 | Columbia-MO | 31 | 51 | 37.9 | 3.9 | 10.2 |  |  |
| R1 | E3 | 2017 | ACRE-IN | 36 | 61 | 45.2 | 3.8 | 8.4 |  |  |
| R1 | E4 | 2018 | ACRE-IN | 36 | 57 | 43.8 | 3.6 | 8.2 |  |  |
| R1 | E5 | 2019 | ACRE-IN | 42 | 55 | 45.6 | 2.2 | 4.8 |  |  |
| R1 | E6 | 2020 | ACRE-IN | 37 | 57 | 45.4 | 3.1 | 6.9 |  |  |
| R1 | E7 | 2019 | Romney-IN | 41 | 53 | 44.4 | 2.2 | 4.9 |  |  |
| R1 | E8 | 2020 | Romney-IN | 37 | 54 | 44.2 | 3.2 | 7.1 |  |  |
| R1 | E9 | 2020 | Gibson-IL | 54 | 80 | 61.9 | 4.4 | 7.1 |  |  |
| R8 | All environments |  | All environments | 110 | 169 | 130.2 | 13.7 | 10.5 | 69 | 54 |
| R8 | E1 | 2017 | Columbia-MO | 115 | 134 | 126.9 | 3.4 | 2.7 |  |  |
| R8 | E2 | 2018 | Columbia-MO | 116 | 142 | 128.0 | 5.3 | 4.2 |  |  |
| R8 | E3 | 2017 | ACRE-IN | 112 | 140 | 121.9 | 8.9 | 7.4 |  |  |
| R8 | E4 | 2018 | ACRE-IN | 115 | 142 | 123.7 | 5.56 | 4.5 |  |  |
| R8 | E5 | 2019 | ACRE-IN | 110 | 135 | 121.1 | 4.8 | 3.9 |  |  |
| R8 | E6 | 2020 | ACRE-IN | 117 | 141 | 129.9 | 5.7 | 4.4 |  |  |
| R8 | E7 | 2019 | Romney-IN | 110 | 136 | 120.8 | 5.3 | 4.4 |  |  |
| R8 | E8 | 2020 | Romney-IN | 114 | 136 | 124.4 | 4.9 | 3.9 |  |  |
| R8 | E9 | 2020 | Gibson-IL | 141 | 169 | 152.9 | 7.2 | 4.7 |  |  |
| RL | All environments |  | All environments | 55 | 111 | 81.7 | 8.46 | 10.4 | 57 | 50 |
| RL | E1 | 2017 | Columbia-MO | 60 | 88 | 77.29 | 4.4 | 5.7 |  |  |
| RL | E2 | 2018 | Columbia-MO | 73 | 105 | 90.03 | 5.03 | 5.6 |  |  |
| RL | E3 | 2017 | ACRE-IN | 55 | 94 | 76.79 | 8.46 | 11.0 |  |  |
| RL | E4 | 2018 | ACRE-IN | 67 | 95 | 79.89 | 5.35 | 6.7 |  |  |
| RL | E5 | 2019 | ACRE-IN | 63 | 89 | 45.47 | 4.57 | 10.1 |  |  |
| RL | E6 | 2020 | ACRE-IN | 65 | 102 | 84.67 | 5.35 | 6.3 |  |  |
| RL | E7 | 2019 | Romney-IN | 61 | 87 | 76.46 | 5.46 | 7.1 |  |  |
| RL | E8 | 2020 | Romney-IN | 66 | 95 | 80.14 | 5.33 | 6.7 |  |  |
| RL | E9 | 2020 | Gibson-IL | 71 | 111 | 90.96 | 6.48 | 7.1 |  |  |

Table S3. Descriptive statistics of phenotypic variation, genotypic variance (G) and broad-sense heritability (H^2^) of days to flowering (R1), days to maturity (R8), and reproductive length for 329 *G.* max USDA accessions evaluated at nine environments.

^a^ Individual environments represent the combination of location and year.

Table S4. Percentage of SNPs by heterochromatic and Eurochromatic regions of each chromosome.

| Chromosome | Percentage of SNPs (%) | |
| --- | --- | --- |
|  | **Heterochromatin** | **Euchromatin** |
| 1 | 31 | 69 |
| 2 | 13 | 87 |
| 3 | 23 | 77 |
| 4 | 39 | 61 |
| 5 | 14 | 86 |
| 6 | 11 | 89 |
| 7 | 11 | 89 |
| 8 | 15 | 85 |
| 9 | 41 | 59 |
| 10 | 32 | 68 |
| 11 | 36 | 64 |
| 12 | 25 | 75 |
| 13 | 29 | 71 |
| 14 | 38 | 62 |
| 15 | 15 | 85 |
| 16 | 22 | 78 |
| 17 | 22 | 78 |
| 18 | 16 | 84 |
| 19 | 33 | 67 |
| 20 | 33 | 67 |
| Total | 24 | 76 |

Table S5. Linkage Disequilibrium (LD) decay rate across 20 chromosomes within euchromatin and heterochromatin regions.

| Chr | LD decay rate (kb) | |
| --- | --- | --- |
|  | **Euchromatin** | **Heterochromatin** |
| 1 | 350 | 8350 |
| 2 | 2250 | 4350 |
| 3 | 1450 | 4750 |
| 4 | 450 | 9150 |
| 5 | 2250 | 7550 |
| 6 | 1450 | 8750 |
| 7 | 6450 | 9550 |
| 8 | 8250 | 8650 |
| 9 | 650 | 6050 |
| 10 | 2350 | 8250 |
| 11 | 650 | 4350 |
| 12 | 450 | 7850 |
| 13 | 3750 | 7450 |
| 14 | 250 | 8850 |
| 15 | 1850 | 9750 |
| 16 | 650 | 7950 |
| 17 | 1550 | 6050 |
| 18 | 2650 | 5150 |
| 19 | 3550 | 9650 |
| 20 | 9450 | 7750 |
| Across all | 2150 | 8650 |

Table S6. Summary of single-nucleotide polymorphisms (SNPs) significantly associated with flowering time (R1), maturity time (R8), and reproductive length (RL) in three hundred twenty-nine G. max accessions across environments and years.

| Chr | SNP Wm82.a2^a^ | SoySNP50k_ID^a^ | Chr Reg^b^ | Loc Genome ^a^ | Trait | | -log(p-val) | Var Exp | Eff | Reported QTLs^a^ | Known *E* Genes ^c^ | Distance (kb) from known *E* genes^c^ |  |
| --- | --- | --- | --- | --- | --- | --- | --- | --- | --- | --- | --- | --- | --- |
|  |  |  |  |  |  |  |  |  |  |  |  |  |  |
| 10 | Gm10_41455680_G_A* | [ss715607080](http://www.ncbi.nlm.nih.gov/projects/SNP/snp_ss.cgi?ss=ss715607080) | Eu | Int | | R1 | 4.4 | 0.05 | 1.79 | R8 full maturity 10-g4.1, Reproductive stage length 4-g2.1, Reproductive stage length 4-g2.2, Reproductive period 3-g5, Seed protein 7-g7 and seed weight 4-g10 | E2 | 3839 |  |
| 4 | Gm04_17228343_T_C* | [ss715587206](http://www.ncbi.nlm.nih.gov/projects/SNP/snp_ss.cgi?ss=ss715587206) | Het | Int | | R8 | 4.71 | 0.02 | -1.85 | - | E1La  E1Lb | 19530  8892 |  |
| 4 | Gm04_17075267_G_A* | [ss715587203](http://www.ncbi.nlm.nih.gov/projects/SNP/snp_ss.cgi?ss=ss715587203) | Het | Int | | R8 | 4.4 | 0.02 | -1.77 | - | E1La  E1Lb | 19683  9045 |  |
| 4 | Gm04_40009617_C_T* | [ss715587845](http://www.ncbi.nlm.nih.gov/projects/SNP/snp_ss.cgi?ss=ss715587845) | Het | Int | | R8 | 4.66 | 0.02 | -1.77 | - | E1La  E1Lb | 3251  13889 |  |
| 4 | Gm04_40276263_A_G* | ss715587857 | Het | Int | | R8 | 4.7 | 0.02 | -1.76 | - | E1La  E1Lb | 3517  26120 |  |
| 4 | Gm04_16673792_G_A | [ss715587192](http://www.ncbi.nlm.nih.gov/projects/SNP/snp_ss.cgi?ss=ss715587192) | Het | Int | | R8 | 4.4 | 0.02 | -1.77 | - | E1La  E1Lb | 20084  9446 |  |
| 4 | Gm04_16889396_T_C | [ss715587197](http://www.ncbi.nlm.nih.gov/projects/SNP/snp_ss.cgi?ss=ss715587197) | Het | Int | | R8 | 4.4 | 0.019 | -1.77 | - | E1La  E1Lb | 19869  9231 |  |
| 20 | Gm20_46615517_A_C | [ss715638773](http://www.ncbi.nlm.nih.gov/projects/SNP/snp_ss.cgi?ss=ss715638773) | Eu | Int | | R8 | 4.33 | 0.02 | -1.36 | First flower 6-g4, R8 full maturity 8-g13, Reproductive period 3-g1 | E4 | 13374 |  |
| 4 | Gm04_39977826_G_A | [ss715587844](http://www.ncbi.nlm.nih.gov/projects/SNP/snp_ss.cgi?ss=ss715587844) | Het | Int | | R8 | 4.17 | 0.01 | -1.6 | - | E1La  E1Lb | 3219  13857 |  |
| 4 | Gm04_40151473_T_C* | [ss715587852](http://www.ncbi.nlm.nih.gov/projects/SNP/snp_ss.cgi?ss=ss715587852) | Het | Int | | R8 | 4.18 | 0.01 | -1.68 | - | E1La  E1Lb | 3393  14031 |  |
| 4 | Gm04_40218961_G_A* | [ss715587856](http://www.ncbi.nlm.nih.gov/projects/SNP/snp_ss.cgi?ss=ss715587856) | Het | Int | | R8 | 4.18 | 0.01 | -1.68 | - | E1La  E1Lb | 3461  14098 |  |
| 3 | Gm03_36427644_C_T | [ss715585727](http://www.ncbi.nlm.nih.gov/projects/SNP/snp_ss.cgi?ss=ss715585727) | Het | CDS | | RL | 4.17 | 0.04 | -1.62 | First flower 4-g10, First flower 3-g2, R8 full maturity 3-g3 | - | - |  |
| 4 | Gm04_40151473_T_C* | [ss715587852](http://www.ncbi.nlm.nih.gov/projects/SNP/snp_ss.cgi?ss=ss715587852) | Het | Int | | RL | 5.63 | 0.05 | -1.92 | - | E1La  E1Lb | 3393  14031 |  |
| 4 | Gm04_40218961_G_A* | [ss715587856](http://www.ncbi.nlm.nih.gov/projects/SNP/snp_ss.cgi?ss=ss715587856) | Het | Int | | RL | 5.63 | 0.05 | -1.92 | - | E1La  E1Lb | 3461  14098 |  |
| 4 | Gm04_17228343_T_C* | [ss715587206](http://www.ncbi.nlm.nih.gov/projects/SNP/snp_ss.cgi?ss=ss715587206) | Het | Int | | RL | 4.24 | 0.04 | -1.68 | - | E1La  E1Lb | 19530  8892 |  |
| 4 | Gm04_40009617_C_T* | [ss715587845](http://www.ncbi.nlm.nih.gov/projects/SNP/snp_ss.cgi?ss=ss715587845) | Het | Int | | RL | 4.35 | 0.04 | -1.65 | - | E1La  E1Lb | 3251  13889 |  |
| 4 | Gm04_39006019_T_C | [ss715587808](http://www.ncbi.nlm.nih.gov/projects/SNP/snp_ss.cgi?ss=ss715587808) | Het | Int | | RL | 4.22 | 0.04 | -1.58 | - | E1La  E1Lb | 2248  12885 |  |
| 4 | Gm04_39484148_T_C | [ss715587823](http://www.ncbi.nlm.nih.gov/projects/SNP/snp_ss.cgi?ss=ss715587823) | Het | Int | | RL | 4.22 | 0.04 | -1.58 | - | E1La  E1Lb | 2726  13364 |  |
| 4 | Gm04_39731223_A_G | [ss715587834](http://www.ncbi.nlm.nih.gov/projects/SNP/snp_ss.cgi?ss=ss715587834) | Het | Int | | RL | 4.22 | 0.04 | -1.58 | - | E1La  E1Lb | 2972  13611 |  |

Chr is chromosome, SNP Wm82.a2 is the SNP positions based on Wm82.a2, Chr Reg is chromosomal region where SNP is located, Loc Genome is the location in the genome, Var Exp is variance explained, Eff is the SNP effect, Het is Heterochromatin, Eu is Euchromatin, Int is intergenic, CDS coding sequence, R8 is days to maturity, RL is reproductive length, R1 days to flowering, ^a^ Information obtained from SoyBase; ^b^ Information obtained from Wen et al., (2015); ^c^ Information obtained from Zimmer et al., (2021); * SNPs selected for candidate gene selection (tagging SNPs).

Table S7. Summary of significant single-nucleotide polymorphisms (SNPs) associated with flowering time (R1), maturity time (R8), and reproductive length (RL) in three hundred twenty-nine G. max accessions.

| Chr | SNP ID^a^ | Pos Wm82.a1^b^ | Pos Wm82.a2^b^ | Chr Reg^c^ | Loc Genome^b^ |  | Env^d^ | Trait | -log(p-val) | Var Exp | Eff |
| --- | --- | --- | --- | --- | --- | --- | --- | --- | --- | --- | --- |
| Gm10 | 40905094_T_C | 40905094 | 41451890 | Eu | Int |  | 2017 | R1 | 4.23 | 0.11 | 1.45 |
| Gm10 | 40908884_G_A | 40908884 | 41455680 | Eu | Int |  | 2017 | R1 | 4.46 | 0.115 | 1.49 |
| Gm10 | 40908884_G_A | 40908884 | 41455680 | Eu | Int |  | All_ENV | R1 | 4.4 | 0.05 | 1.79 |
| Gm18 | 57279697_A_G | 57279697 | 53010195 | Eu | Int |  | Romney, IN_2019 | R1 | 4.13 | 0.09 | 0.65 |
| Gm18 | 57279697_A_G | 57279697 | 53010195 | Eu | Int |  | 2019 | R1 | 4.24 | 0.651 | 0.62 |
| Gm18 | 57279697_A_G | 57279697 | 53010195 | Eu | Int |  | 2020 | R1 | 4.25 | 0.013 | 1.05 |
| Gm18 | 57401711_A_G | 57401711 | 53132227 | Eu | Int |  | 2017 | R1 | 4.6 | 0.037 | 0.85 |
| Gm18 | 57401711_A_G | 57401711 | 53132227 | Eu | Int |  | 2019 | R1 | 4.21 | 0.649 | 0.62 |
| Gm18 | 57569299_A_G | 57569299 | 53299447 | Eu | Int |  | 2017 | R1 | 4.34 | 0.045 | 0.93 |
| Gm03 | 38448001_C_T | 38448001 | 36427644 | Eu | CDS |  | Romney, IN_2019 | R8 | 4.64 | 0.15 | -1.51 |
| Gm03 | 38448001_C_T | 38448001 | 36427644 | Eu | CDS |  | 2019 | R8 | 4.37 | 0.15 | -1.73 |
| Gm04 | 15633879_G_A | 15633879 | 16673792 | Het | Int |  | ACRE, IN_2019 | R8 | 4.19 | 0.22 | -2.16 |
| Gm04 | 15633879_G_A | 15633879 | 16673792 | Het | Int |  | Romney, IN_2019 | R8 | 4.98 | 0.17 | -1.60 |
| Gm04 | 15633879_G_A | 15633879 | 16673792 | Het | Int |  | 2019 | R8 | 4.28 | 0.073 | -1.83 |
| Gm04 | 15633879_G_A | 15633879 | 16673792 | Het | Int |  | All_ENV | R8 | 4.4 | 0.02 | -1.74 |
| Gm04 | 15667941_T_C | 15667941 | 16707992 | Het | Int |  | ACRE, IN_2019 | R8 | 4.22 | 0.21 | -2.15 |
| Gm04 | 15667941_T_C | 15667941 | 16707992 | Het | Int |  | Romney, IN_2019 | R8 | 4.47 | 0.15 | -1.49 |
| Gm04 | 15849345_T_C | 15849345 | 16889396 | Het | Int |  | ACRE, IN_2019 | R8 | 4.19 | 0.22 | -2.16 |
| Gm04 | 15849345_T_C | 15849345 | 16889396 | Het | Int |  | Romney, IN_2019 | R8 | 4.98 | 0.17 | -1.60 |
| Gm04 | 15849345_T_C | 15849345 | 16889396 | Het | Int |  | 2019 | R8 | 4.60 | 0.17 | -1.83 |
| Gm04 | 15849345_T_C | 15849345 | 16889396 | Het | Int |  | All_ENV | R8 | 4.25 | 0.019 | -1.74 |
| Gm04 | 16031274_G_A | 16031274 | 17075267 | Het | Int |  | ACRE, IN_2019 | R8 | 4.19 | 1.18 | -2.16 |
| Gm04 | 16031274_G_A | 16031274 | 17075267 | Het | Int |  | Romney, IN_2019 | R8 | 4.98 | 0.17 | -1.60 |
| Gm04 | 16031274_G_A | 16031274 | 17075267 | Het | Int |  | 2019 | R8 | 4.60 | 0.17 | -1.83 |
| Gm04 | 16031274_G_A | 16031274 | 17075267 | Het | Int |  | All_ENV | R8 | 4.4 | 0.02 | -1.77 |
| Gm04 | 16183920_T_C | 16183920 | 17228343 | Het | Int |  | Columbia, MO_2017 | R8 | 4.32 | 0.45 | -1.57 |
| Gm04 | 16183920_T_C | 16183920 | 17228343 | Het | Int |  | ACRE, IN_2019 | R8 | 4.33 | 0.23 | -2.22 |
| Gm04 | 16183920_T_C | 16183920 | 17228343 | Het | Int |  | Romney, IN_2019 | R8 | 4.76 | 0.16 | -1.57 |
| Gm04 | 16183920_T_C | 16183920 | 17228343 | Het | Int |  | 2019 | R8 | 4.53 | 0.17 | -1.82 |
| Gm04 | 16183920_T_C | 16183920 | 17228343 | Het | Int |  | All_ENV | R8 | 4.71 | 0.02 | -1.85 |
| Gm04 | 36874657_C_T | 36874657 | 40009617 | Het | Int |  | ACRE, IN_2018 | R8 | 4.51 | 0.20 | -2.44 |
| Gm04 | 36874657_C_T | 36874657 | 40009617 | Het | Int |  | ACRE, IN_2019 | R8 | 4.32 | 0.21 | -2.13 |
| Gm04 | 36874657_C_T | 36874657 | 40009617 | Het | Int |  | 2019 | R8 | 4.55 | 0.20 | -1.75 |
| Gm04 | 36874657_C_T | 36874657 | 40009617 | Het | Int |  | All_ENV | R8 | 4.66 | 0.02 | -1.77 |
| Gm04 | 37010886_T_C | 37010886 | 40151473 | Het | Int |  | Romney, IN_2019 | R8 | 4.64 | 0.15 | -1.51 |
| Gm04 | 37010886_T_C | 37010886 | 40151473 | Het | Int |  | 2019 | R8 | 4.37 | 0.15 | -1.73 |
| Gm04 | 37078558_G_A | 37078558 | 40218961 | Het | Int |  | Romney, IN_2019 | R8 | 4.64 | 0.15 | -1.51 |
| Gm04 | 37078558_G_A | 37078558 | 40218961 | Het | Int |  | 2019 | R8 | 4.37 | 0.15 | -1.73 |
| Gm04 | 37126858_A_G | 37126858 | 40276263 | Het | Int |  | ACRE, IN_2018 | R8 | 4.20 | 0.19 | -2.32 |
| Gm04 | 37126858_A_G | 37126858 | 40276263 | Het | Int |  | ACRE, IN_2019 | R8 | 4.27 | 0.20 | -2.09 |
| Gm04 | 37126858_A_G | 37126858 | 40276263 | Het | Int |  | 2019 | R8 | 4.47 | 0.15 | -1.71 |
| Gm04 | 37126858_A_G | 37126858 | 40276263 | Het | Int |  | All_ENV | R8 | 4.68 | 0.02 | -1.76 |
| Gm18 | 54531027_T_C | 54531027 | 50257198 | Eu | CDS |  | Columbia, MO_2017 | R8 | 5.00 | 0.24 | -1.63 |
| Gm18 | 54562662_T_C | 54562662 | 50288833 | Eu | CDS |  | Columbia, MO_2017 | R8 | 4.73 | 0.17 | -1.38 |
| Gm20 | 44509848_C_T | 44509848 | 45638820 | Eu | 5UTR |  | 2017 | R8 | 4.19 | 0.003 | 0.42 |
| Gm20 | 45487288_A_C | 45487288 | 46615517 | Eu | Int |  | 2017 | R8 | 4.88 | 0.004 | 0.46 |
| Gm20 | 45487288_A_C | 45487288 | 46615517 | Eu | Int |  | All_ENV | R8 | 4.33 | 0.02 | -1.36 |
| Gm03 | 38448001_C_T | 38448001 | 36427644 | Eu | CDS |  | ACRE, IN_2020 | RL | 4.51 | 0.15 | -2.04 |
| Gm03 | 38448001_C_T | 38448001 | 36427644 | Eu | CDS |  | 2020 | RL | 4.49 | 0.07 | -1.95 |
| Gm03 | 38448001_C_T | 38448001 | 36427644 | Eu | CDS |  | All_ENV | RL | 4.17 | 0.04 | -1.62 |
| Gm04 | 15633879_G_A | 15633879 | 16673792 | Het | Int |  | ACRE, IN_2018 | RL | 4.92 | 0.18 | -2.20 |
| Gm04 | 15633879_G_A | 15633879 | 16673792 | Het | Int |  | 2020 | RL | 4.26 | 0.07 | -1.93 |
| Gm04 | 15667941_T_C | 15667941 | 16707992 | Het | Int |  | ACRE, IN 2018 | RL | 5.41 | 0.19 | -2.30 |
| Gm04 | 15667941_T_C | 15667941 | 16707992 | Het | Int |  | ACRE, IN_2019 | RL | 4.65 | 0.19 | -1.95 |
| Gm04 | 15849345_T_C | 15849345 | 16889396 | Het | Int |  | ACRE, IN_2018 | RL | 4.92 | 0.18 | -2.20 |
| Gm04 | 15849345_T_C | 15849345 | 16889396 | Het | Int |  | 2020 | RL | 4.26 | 0.07 | -1.93 |
| Gm04 | 16031274_G_A | 16031274 | 17075267 | Het | Int |  | ACRE, IN_2018 | RL | 4.92 | 0.18 | -2.20 |
| Gm04 | 16031274_G_A | 16031274 | 17075267 | Het | Int |  | 2020 | RL | 4.26 | 0.07 | -1.93 |
| Gm04 | 16183920_T_C | 16183920 | 17228343 | Het | Int |  | ACRE, IN_2018 | RL | 5.37 | 0.20 | -2.33 |
| Gm04 | 16183920_T_C | 16183920 | 17228343 | Het | Int |  | ACRE, IN_2019 | RL | 4.31 | 0.18 | -1.90 |
| Gm04 | 16183920_T_C | 16183920 | 17228343 | Het | Int |  | Romney, IN_2020 | RL | 4.23 | 0.12 | -1.77 |
| Gm04 | 16183920_T_C | 16183920 | 17228343 | Het | Int |  | 2019 | RL | 4.31 | 0.13 | -1.54 |
| Gm04 | 16183920_T_C | 16183920 | 17228343 | Het | Int |  | 2020 | RL | 5.00 | 0.08 | -2.13 |
| Gm04 | 16183920_T_C | 16183920 | 17228343 | Het | Int |  | All_ENV | RL | 4.24 | 0.04 | -1.68 |
| Gm04 | 35879410_T_C | 35879410 | 39006019 | Het | Int |  | All_ENV | RL | 4.22 | 0.04 | -1.58 |
| Gm04 | 36357346_T_C | 36357346 | 39484148 | Het | Int |  | All_ENV | RL | 4.22 | 0.04 | -1.58 |
| Gm04 | 36604337_A_G | 36604337 | 39731223 | Het | Int |  | All_ENV | RL | 4.22 | 0.04 | -1.58 |
| Gm04 | 36874657_C_T | 36874657 | 40009617 | Het | Int |  | ACRE, IN_2019 | RL | 4.29 | 0.17 | -1.81 |
| Gm04 | 36874657_C_T | 36874657 | 40009617 | Het | Int |  | Gibson, IL_2020 | RL | 4.54 | 0.11 | -2.37 |
| Gm04 | 36874657_C_T | 36874657 | 40009617 | Het | Int |  | 2019 | RL | 4.58 | 0.13 | -1.52 |
| Gm04 | 36874657_C_T | 36874657 | 40009617 | Het | Int |  | 2020 | RL | 4.40 | 0.06 | -1.89 |
| Gm04 | 36874657_C_T | 36874657 | 40009617 | Het | Int |  | All_ENV | RL | 4.35 | 0.04 | -1.65 |
| Gm04 | 37010886_T_C | 37010886 | 40151473 | Het | Int |  | ACRE, IN_2020 | RL | 4.51 | 0.15 | -2.04 |
| Gm04 | 37010886_T_C | 37010886 | 40151473 | Het | Int |  | 2020 | RL | 4.49 | 0.07 | -1.95 |
| Gm04 | 37010886_T_C | 37010886 | 40151473 | Het | Int |  | All_ENV | RL | 5.63 | 0.05 | -1.92 |
| Gm04 | 37078558_G_A | 37078558 | 40218961 | Het | Int |  | ACRE, IN_2020 | RL | 4.51 | 0.15 | -2.04 |
| Gm04 | 37078558_G_A | 37078558 | 40218961 | Het | Int |  | Gibson, IL_2020 | RL | 4.63 | 0.11 | -2.39 |
| Gm04 | 37078558_G_A | 37078558 | 40218961 | Het | Int |  | 2020 | RL | 4.49 | 0.07 | -1.95 |
| Gm04 | 37078558_G_A | 37078558 | 40218961 | Het | Int |  | All_ENV | RL | 5.63 | 0.05 | -1.92 |
| Gm04 | 37126858_A_G | 37126858 | 40276263 | Het | Int |  | ACRE, IN 2019 | RL | 4.15 | 0.16 | -1.76 |
| Gm04 | 37126858_A_G | 37126858 | 40276263 | Het | Int |  | 2019 | RL | 4.26 | 0.12 | -1.45 |
| Gm04 | 37126858_A_G | 37126858 | 40276263 | Het | Int |  | 2020 | RL | 4.56 | 0.07 | -1.91 |

Chr is chromosome, Pos Wm82.a1 is SNPs position in Wm82.a1.v1 genome assembly, Pos Wm82.a2 is SNPs position in Wm82.a2.v1 genome assembly, Chr Reg is chromosomal region where SNP is located, Loc Genome is the location in the genome, Var Exp is variance explained, Eff is the SNP effect, Het is Heterochromatin, Eu is Euchromatin, Int is intergenic, CDS coding sequence, R8 is days to maturity, RL is reproductive length, R1 days to flowering, ^a^all SNP IDs start with “BARC_1.01_Gm” and the number of chromosome and the positions are based on Wm82.a1.v1 genome assembly; ^b^Information obtained from SoyBase; ^c^ Information obtained from Wen et al., (2015); ^d^The significant SNPs were obtained from the association analysis of individual environments, years, and across all environments and years; * SNPs selected for candidate gene selection (tagging SNPs).

Table S8. List of potential candidate genes by tagging SNPs with average accuracy and descriptions for flowering time (R1), maturity time (R8), and reproductive length (RL).

| **Gene** | **Chr** | **Gene Pos^a^** | **Tag SNPs ID** | **QTL peak** | **Trait** | **Avg Acc %^c^** | **Description** | **Gene name TAIR^d^** | **Gene Bank ID^e^** |
| --- | --- | --- | --- | --- | --- | --- | --- | --- | --- |
| Glyma.04g123700 | Gm04 | 16002630-16006606 | 15633879_G_A | Peak 1 | R8/RL | 87.60 | Solute: sodium symporters, urea transmembrane transporters | AT5G45380.1 | - |
| Glyma.04g123800 | Gm04 | 16010092-16013287 | 15633879_G_A | Peak 1 | R8/RL | 87.31 | Alternative oxidase | - | - |
| Glyma.04g123900 | Gm04 | 16016060-16026862 | 15633879_G_A | Peak 1 | R8/RL | 86.76 | Heteroglycan glucosidase 1 | AT3G23640.1 | - |
| Glyma.04g124000 | Gm04 | 16034262-16035430 | 15633879_G_A | Peak 1 | R8/RL | 87.52 | Unknown protein | - | - |
| Glyma.04g124100 | Gm04 | 16077062-16078008 | 15633879_G_A/16031274_G_A | Peak 1 | R8/RL | 88.70 | Disease resistance-responsive (dirigent-like protein) family protein | AT1G65870.1 | - |
| Glyma.04g124200 | Gm04 | 16077062-16078008 | 15633879_G_A/16031274_G_A | Peak 1 | R8/RL | 88.55 | BZIP transcription factor/ABA-responsive element binding protein 3 | AT3G56850.1 | - |
| Glyma.04g124300 | Gm04 | 16092707-16099713 | 15633879_G_A/16031274_G_A | Peak 1 | R8/RL | 88.40 | Far-red elongated hypocotyls 3 | AT3G22170.1 | - |
| Glyma.04g124400 | Gm04 | 16123330-16126794 | 15633879_G_A/16031274_G_A | Peak 1 | R8/RL | 88.91 | Unknown protein | - | - |
| Glyma.04g124500 | Gm04 | 16284712-16286641 | 15633879_G_A/16031274_G_A | Peak 1 | R8/RL | 88.50 | Protein of unknown function (DUF1191) | AT4G22900.1 | - |
| Glyma.04g124600 | Gm04 | 16329983-16332898 | 15633879_G_A/16031274_G_A | Peak 1 | R8/RL | 88.32 | FAR1-related sequence 5 | AT4G38180.1 | - |
| Glyma.04g124700 | Gm04 | 16346707-16347780 | 15633879_G_A | Peak 1 | R8/RL | 86.00 | Unknown protein | - | - |
| Glyma.04g124800 | Gm04 | 16368438-16390781 | 15633879_G_A/16031274_G_A | Peak 1 | R8/RL | 89.60 | Zinc induced facilitator-like 1 | AT5G13750.1 | - |
| Glyma.04g124900 | Gm04 | 16399212-16402624 | 15633879_G_A/16031274_G_A | Peak 1 | R8/RL | 90.30 | Uncharacterized protein LOC102668196 isoform X1 [Glycine max] | AT1G48560.2 | - |
| Glyma.04g125000 | Gm04 | 16409044-16409645 | 15633879_G_A/16031274_G_A | Peak 1 | R8/RL | 89.70 | NB-ARC domain-containing disease resistance protein | AT3G07040.1 | - |
| Glyma.04g125100 | Gm04 | 16472994-16473578 | 15633879_G_A/16031274_G_A | Peak 1 | R8/RL | 89.63 | SOS3-interacting protein 3 | AT4G30960.1 | - |
| Glyma.04g125200 | Gm04 | 16474623-16475810 | 15633879_G_A/16031274_G_A | Peak 1 | R8/RL | 89.86 | Regulator of Vps4 activity in the MVB pathway protein | AT4G35730.1 | - |
| Glyma.04g125300 | Gm04 | 16500908-16508912 | 15633879_G_A/16031274_G_A | Peak 1 | R8/RL | 89.43 | NB-ARC domain-containing disease resistance protein | AT3G07040.1 | - |
| Glyma.04g125400 | Gm04 | 16529335-16529723 | 15633879_G_A/16031274_G_A | Peak 1 | R8/RL | 88.70 | GRF zinc finger | - | - |
| Glyma.04g125500 | Gm04 | 16532221-16536664 | 15633879_G_A/16031274_G_A | Peak 1 | R8/RL | 90.00 | SU(VAR)3-9 homolog 3 | AT1G73100.1 | - |
| Glyma.04g125600 | Gm04 | 16622379-16634652 | 15633879_G_A/16031274_G_A | Peak 1 | R8/RL | 94.75 | Zinc ion binding | AT3G54360.1 | - |
| Glyma.04g125700 | Gm04 | 16709512-16714749 | 15633879_G_A/16031274_G_A | Peak 1 | R8/RL | 91.97 | Myb domain protein 33 | AT5G06100.2 | - |
| Glyma.04g125800 | Gm04 | 16726595-16738293 | 15633879_G_A/16031274_G_A | Peak 1 | R8/RL | 91.82 | Vacuolar protein sorting 26A | AT5G53530.1 | - |
| Glyma.04g125900 | Gm04 | 16809159-16809753 | 15633879_G_A/16031274_G_A | Peak 1 | R8/RL | 94.19 | Protein kinase superfamily protein | AT5G56790.1 | - |
| Glyma.04g126000 | Gm04 | 16811865-16817931 | 15633879_G_A/16031274_G_A | Peak 1 | R8/RL | 92.78 | Protein kinase superfamily protein | AT5G56790.1 | - |
| Glyma.04g126100 | Gm04 | 16937746-16942191 | 15633879_G_A/16031274_G_A | Peak 1 | R8/RL | 92.50 | Unknown protein | AT2G38695.1 | - |
| Glyma.04g126300 | Gm04 | 16964286-16965431 | 15633879_G_A | Peak 1 | R8/RL | 85.30 | Membrane steroid binding protein 1 | AT5G52240.1 | - |
| Glyma.04g126400 | Gm04 | 17000680-17005885 | 15633879_G_A/16031274_G_A | Peak 1 | R8/RL | 95.23 | Global transcription factor group B1 | AT1G65440.2 | - |
| Glyma.04g126500 | Gm04 | 17010198-17015041 | 15633879_G_A/16183920_T_C | Peak 1 | R8/RL | 92.51 | Multidrug resistance-associated protein 2 | AT2G34660.1 | - |
| Glyma.04g126600 | Gm04 | 17030276-17031139 | 16183920_T_C/15633879_G_A | Peak 1 | R8/RL | 93.20 | BTB/POZ domain-containing protein | AT4G10800.1 | - |
| Glyma.04g126700 | Gm04 | 17057705-17060281 | 16183920_T_C | Peak 1 | R8/RL | 92.46 | ARM repeat superfamily protein | AT1G12930.1 | - |
| Glyma.04g126800 | Gm04 | 17060524-17063365 | 15633879_G_A | Peak 1 | R8/RL | 90.89 | Translation initiation factor 2, small GTP-binding protein | AT4G11160.1 | - |
| Glyma.04g126900 | Gm04 | 17096980-17102005 | 15633879_G_A/16031274_G_A | Peak 1 | R8/RL | 94.50 | Uridine-ribohydrolase 1 | AT2G36310.1 | - |
| Glyma.04g127000 | Gm04 | 17123614-17124372 | 15633879_G_A/16031274_G_A/16183920_T_C | Peak 1 | R8/RL | 94.45 | CBS domain-containing protein with a domain of unknown function (DUF21 | AT1G03270.1 | - |
| Glyma.04g127100 | Gm04 | 17126079-17130669 | 15633879_G_A/16031274_G_A/16183920_T_C | Peak 1 | R8/RL | 93.98 | Cysteine-rich RLK (RECEPTOR-like protein kinase) 10 | AT4G23180.1 | - |
| Glyma.04g127200 | Gm04 | 17136690-17142016 | 15633879_G_A/16031274_G_A/16183920_T_C | Peak 1 | R8/RL | 93.24 | Unknown protein | - | - |
| Glyma.04g127300 | Gm04 | 17182350-17182837 | 15633879_G_A/16031274_G_A/16183920_T_C | Peak 1 | R8/RL | 94.46 | Unknown protein | - | - |
| Glyma.04g127400 | Gm04 | 17255113-17256083 | 15633879_G_A/16031274_G_A | Peak 1 | R8/RL | 89.18 | Unknown protein | AT4G02550.2 | - |
| Glyma.04g127500 | Gm04 | 17256467-1725,923 | 15633879_G_A/16031274_G_A/16183920_T_C | Peak 1 | R8/RL | 92.49 | Pentatricopeptide repeat (PPR) superfamily protein | AT1G11290.1 | - |
| Glyma.04g127600 | Gm04 | 17386901-17387809 | 15633879_G_A/16031274_G_A | Peak 1 | R8/RL | 89.17 | C2H2 and C2HC zinc fingers superfamily protein | AT5G10970.1 | - |
| Glyma.04g127700 | Gm04 | 17503761-17506319 | 16031274_G_A15633879_G_A | Peak 1 | R8/RL | 88.00 | BED zinc finger, hAT family dimerisation domain | AT3G42170.1 | - |
| Glyma.04g127800 | Gm04 | 17506510-17506734 | 15633879_G_A/16031274_G_A | Peak 1 | R8/RL | 89.45 | Unknown protein | - | - |
| Glyma.04g127900 | Gm04 | 17508855-17512554 | 15633879_G_A | Peak 1 | R8/RL | 87.18 | Nodulin MtN21 /EamA-like transporter family protein | AT5G07050.1 | - |
| Glyma.04g128000 | Gm04 | 17687258-17689497 | 16031274_G_A | Peak 1 | R8/RL | 90.80 | Unknown protein | - | - |
| Glyma.04g128400 | Gm04 | 17759715-17762227 | 16031274_G_A | Peak 1 | R8/RL | 91.18 | Zinc ion binding | AT2G44580.1 | - |
| Glyma.04g128500 | Gm04 | 17809136-17810282 | 16031274_G_A | Peak 1 | R8/RL | 90.20 | Late embryogenesis abundant protein, group 1 protein | AT1G32560.1 | - |
| Glyma.04g128600 | Gm04 | 17866415-17867698 | 16031274_G_A | Peak 1 | R8/RL | 90.25 | Alpha/beta-Hydrolases superfamily protein | AT1G68620.1 | - |
| Glyma.04g128700 | Gm04 | 17902621-17908873 | 16031274_G_A | Peak 1 | R8/RL | 89.04 | SCP1-like small phosphatase 4 | AT5G46410.2 | - |
| Glyma.04g128800 | Gm04 | 17927529-17936517 | 16031274_G_A | Peak 1 | R8/RL | 88.93 | C2H2 zinc-finger protein SERRATE (SE) | AT2G27100.1 | - |
| Glyma.04g128900 | Gm04 | 17931394-17931507 | 16031274_G_A | Peak 1 | R8/RL | 89.10 | Unknown protein | - | - |
| Glyma.04g129000 | Gm04 | 17964578-17969250 | 16031274_G_A | Peak 1 | R8/RL | 89.90 | Unknown protein | AT5G04910.1 | - |
| Glyma.04g129100 | Gm04 | 17974120-17976827 | 16031274_G_A/16183920_T_C | Peak 1 | R8/RL | 90.92 | Tetratricopeptide repeat (TPR)-like superfamily protein | AT3G57430.1 | - |
| Glyma.04g129200 | Gm04 | 18028813-18036924 | 16031274_G_A | Peak 1 | R8/RL | 91.29 | Aminophospholipid ATPase 1 | AT5G04930.1 | - |
| Glyma.04g129300 | Gm04 | 18076546-18077311 | 16031274_G_A | Peak 1 | R8/RL | 89.80 | GRAM domain family protein | AT4G01600.1 | - |
| Glyma.04g159300 | Gm04 | 39283912-39295261 | 37078558_G_A | Peak 2 | R8/RL | 90.80 | AGAMOUS-like 8/MADS-box transcription factor 6 [Glycine max] | AT5G60910.1 | - |
| Glyma.04g159400 | Gm04 | 39320127-39321864 | 37078558_G_A | Peak 2 | R8/RL | 90.40 | Unknown protein | - | - |
| Glyma.04g159500 | Gm04 | 39451100-39453905 | 37078558_G_A | Peak 2 | R8/RL | 90.85 | Saposin B domain-containing protein | AT3G51730.1 | - |
| Glyma.04g159600 | Gm04 | 39578853-39583603 | 37078558_G_A | Peak 2 | R8/RL | 90.50 | Squamosa promoter-binding protein-like (SBP domain) transcription factor family protein | AT1G69170.1 | - |
| Glyma.04g159700 | Gm04 | 39586882-39589922 | 37078558_G_A | Peak 2 | R8/RL | 90.40 | Reversibly glycosylated polypeptide 3 | AT3G08900.1 | - |
| Glyma.04g159800 | Gm04 | 39601379-39606991 | 37078558_G_A | Peak 2 | R8/RL | 90.93 | PIF1-like helicase | - | - |
| Glyma.04g159900 | Gm04 | 39633309-39634727 | 37078558_G_A | Peak 2 | R8/RL | 90.73 | Tetratricopeptide repeat (TPR)-like superfamily protein | AT3G23020.1 | - |
| Glyma.04g160000 | Gm04 | 39655881-39658301 | 37078558_G_A | Peak 2 | R8/RL | 91.17 | COBRA-like extracellular glycosyl-phosphatidyl inositol-anchored protein family | AT5G15630.1 | - |
| Glyma.04g160200 | Gm04 | 39725839-39726383 | 37010886_T_C/ 37078558_G_A | Peak 2 | R8/RL | 89.42 | Unknown protein | AT1G21280.1 | - |
| Glyma.04g160300 | Gm04 | 39737018-39741052 | 37010886_T_C/ 37078558_G_A37126858_A_G | Peak 2 | R8/RL | 83.33 | Transducin/WD40 repeat-like superfamily protein | AT5G60940.1 | - |
| Glyma.04g160500 | Gm04 | 39750017-39752209 | 37078558_G_A | Peak 2 | R8/RL | 90.23 | WAPL (Wings apart-like protein regulation of heterochromatin) protein | AT1G11060.1 | - |
| Glyma.04g160600 | Gm04 | 39809314-39814127 | 37078558_G_A | Peak 2 | R8/RL | 90.30 | PIF1 helicase | AT3G51690.1 | - |
| Glyma.04g160700 | Gm04 | 39818871-39825716 | 37078558_G_A | Peak 2 | R8/RL | 89.90 | Galactose oxidase/kelch repeat superfamily protein | AT1G18610.1 | - |
| Glyma.04g160800 | Gm04 | 39840898-39854321 | 37078558_G_A | Peak 2 | R8/RL | 90.20 | Histone-lysine N-methyltransferases | AT3G26850.1 | - |
| Glyma.04g161000 | Gm04 | 39923410-39928355 | 37010886_T_C37078558_G_A | Peak 2 | R8/RL | 92.00 | LONGIFOLIA 2-like isoform X5 [Glycine max]; | AT1G74160.1 | - |
| Glyma.04g161200 | Gm04 | 39942189-39946987 | 37078558_G_A | Peak 2 | R8/RL | 89.51 | Ribosomal protein L31e family protein | AT2G19740.1 | - |
| Glyma.04g161300 | Gm04 | 39978926-39980814 | 37126858_A_G | Peak 2 | R8/RL | 86.04 | Ribosomal protein L1p/L10e family) | AT1G08360.1 | - |
| Glyma.04g161400 | Gm04 | 40001447-40004338 | 37078558_G_A | Peak 2 | R8/RL | 93.75 | TEOSINTE BRANCHED 1, cycloidea and PCF transcription factor 5) | AT5G60970.1 | - |
| Glyma.04g161500 | Gm04 | 40052142-40060432 | 37126858_A_G/ 36874657_C_T | Peak 2 | R8/RL | 85.00 | PIF1 helicase | AT3G51700.1 | - |
| Glyma.04g161600 | Gm04 | 40088117-40090295 | 36874657_C_T | Peak 2 | R8/RL | 94.40 | Protein of unknown function (DUF1118)) | AT5G08050.1 | - |
| Glyma.04g161700 | Gm04 | 40104836-40105385 | 37010886_T_C37078558_G_A | Peak 2 | R8/RL | 92.50 | Myb/SANT-like DNA-binding domain protein | AT4G02550.2 | - |
| Glyma.04g161800 | Gm04 | 40109124-40109663 | 37010886_T_C/37078558_G_A | Peak 2 | R8/RL | 95.00 | (Cyclin/Brf1-like TBP-binding protein) | AT3G09360.1 | - |
| Glyma.04g161900 | Gm04 | 40147800-40148585 | 37010886_T_C37078558_G_A | Peak 2 | R8/RL | 93.81 | Unknown protein | - | - |
| Glyma.04g162000 | Gm04 | 40157051..40157476 | 37010886_T_C37078558_G_A | Peak 2 | R8/RL | 91.89 | Transmembrane protein | AT1G53035.1 | - |
| Glyma.04g162200 | Gm04 | 40212903-40217728 | 37010886_T_C37078558_G_A | Peak 2 | R8/RL | 97.00 | Homeobox-like protein;(source:Araport11) | AT1G74220.1 | - |
| Glyma.04g162300 | Gm04 | 40236769-40236930 | 37010886_T_C37078558_G_A | Peak 2 | R8/RL | 88.50 | Unknown protein | - | - |
| Glyma.04g162400 | Gm04 | 40236981-40239376 | 37078558_G_A | Peak 2 | R8/RL | 95.92 | Exocyst subunit exo70 family protein E2 | AT5G61010.1 | - |
| Glyma.04g162500 | Gm04 | 40265908-40266378 | 37078558_G_A | Peak 2 | R8/RL | 94.01 | Cytochrome oxidase 2 | ATMG00160.1 | - |
| Glyma.04g162600 | Gm04 | 40278029-40282430 | 37010886_T_C37078558_G_A | Peak 2 | R8/RL | 89.00 | Uncharacterized protein LOC100792679 isoform X1 [Glycine max] | AT5G08010.1 | - |
| Glyma.04g162700 | Gm04 | 40326865-40331483 | 37078558_G_A | Peak 2 | R8/RL | 91.07 | 3-phosphoserine phosphatase | AT1G18640.2 | - |
| Glyma.04g162800 | Gm04 | 40387251-40393343 | 37078558_G_A | Peak 2 | R8/RL | 91.00 | Gamma subunit of Mt ATP synthase | AT2G33040.1 | - |
| Glyma.04g162900 | Gm04 | 40388840-40389153 | 37078558_G_A | Peak 2 | R8/RL | 93.20 | Unknown protein | - | - |
| Glyma.04g163000 | Gm04 | 40423906-40429027 | 37010886_T_C37078558_G_A | Peak 2 | R8/RL | 92.10 | Mitochondrial substrate carrier family protein | AT1G74240.1 | - |
| Glyma.04g163100 | Gm04 | 40431122-40438954 | 37078558_G_A | Peak 2 | R8/RL | 92.98 | PHD finger-containing protein. Interacts with BDT1, acts with other PHD proteins to associate with flowering genes and thereby suppress their transcription. | AT5G61120.1 | - |
| Glyma.04g163200 | Gm04 | 40464589-40467748 | 37078558_G_A | Peak 2 | R8/RL | 88.90 | Heat shock protein 101 | AT1G74310.1 | - |
| Glyma.04g163300 | Gm04 | 40483595-40484853 | 37078558_G_A | Peak 2 | R8/RL | 89.95 | Acyl-CoA N-acyltransferases (NAT) superfamily protein | AT4G19985.1 | - |
| Glyma.04g163600 | Gm04 | 40582252-40596618 | 37078558_G_A | Peak 2 | R8/RL | 89.83 | Dentin sialophosphoprotein-like protein | AT5G07940.1 | N |
| Glyma.04g163900 | Gm04 | 40730419-40731291 | 37078558_G_A | Peak 2 | R8/RL | 89.60 | Serine carboxypeptidase-like 46) | AT2G33530.1 | - |
| Glyma.04g164000 | Gm04 | 40803633-40804670 | 37078558_G_A | Peak 2 | R8/RL | 89.90 | Myb/SANT-like DNA-binding domain protein;(source:Araport11) | AT5G05800.1 | - |
| Glyma.04g164100 | Gm04 | 40811977-40820483 | 37078558_G_A | Peak 2 | R8/RL | 89.85 | Leucine-rich repeat (LRR) family protein | AT5G07910.1 | - |
| Glyma.04g164400 | Gm04 | 40973232..40975775 | 37010886_T_C37078558_G_A | Peak 2 | R8/RL | 86.50 | Soluble N-ethylmaleimide-sensitive factor adaptor protein 33 | AT5G61210.1 | - |
| Glyma.04g164500 | Gm04 | 41027892-41033697 | 37010886_T_C37078558_G_A37126858_A_G | Peak 2 | R8/RL | 87.70 | Glucuronidase 2 | AT5G07830.1 | - |
| Glyma.04g164600 | Gm04 | 41086167-41089544 | 37010886_T_C37126858_A_G | Peak 2 | R8/RL | 88.41 | Varicose-related | AT3G13290.1 | - |
| Glyma.04g164700 | Gm04 | 41098678-41099094 | 37010886_T_C37126858_A_G | Peak 2 | R8/RL | 86.11 | Protein of unknown function (DUF1278)) | AT1G76750.1 | - |
| Glyma.04g164800 | Gm04 | 41104671-41105875 | 37126858_A_G | Peak 2 | R8/RL | 84.51 | Remorin family protein | AT5G61280.1 | - |
| Glyma.04g165400 | Gm04 | 41326473-41330652 | 37126858_A_G | Peak 2 | R8/RL | 85.78 | Leucine-rich repeat protein kinase family protein | AT1G74360.1 | - |
| Glyma.10g180000 | Gm10 | 41336339-41337449 | 40908884_G_A | Peak 3 | R1 | 85.92 | AUX/IAA transcriptional regulator family protein) | AT1G04240.1 | - |
| Glyma.10g180200 | Gm10 | 41387259-41392378 | 40908884_G_A | Peak 3 | R1 | 85.99 | Magnesium transporter 9 | AT5G64560.1 | - |
| Glyma.10g180300 | Gm10 | 41398806-41402748 | 40908884_G_A | Peak 3 | R1 | 86.70 | DREB2A-interacting protein 2 | AT2G30580.1 | - |
| Glyma.10g180600 | Gm10 | 41417050-41421842 | 40908884_G_A | Peak 3 | R1 | 85.50 | Cryptochrome 2 | AT1G04400.1 | DQ401047.1 / AB498935.1/AB498936.1/ |
| Glyma.10g180800 | Gm10 | 41440863-41443235 | 40908884_G_A | Peak 3 | R1 | 93.29 | Myb domain protein 15 | AT3G23250.1 | - |
| Glyma.10g180900 | Gm10 | 41454466-41454978 | 40908884_G_A | Peak 3 | R1 | 90.65 | Unknown protein | AT4G14380.1 | - |
| Glyma.10g181000 | Gm10 | 41457399-41458480 | 40908884_G_A | Peak 3 | R1 | 86.11 | Unknown protein | AT4G14380.1 | - |
| Glyma.10g181100 | Gm10 | 41462028-41467447 | 40908884_G_A | Peak 3 | R1 | 86.38 | XB3 ortholog 5 in Arabidopsis thaliana | AT3G23280.2 | - |
| Glyma.10g181600 | Gm10 | 41499182-41500142 | 40908884_G_A | Peak 3 | R1 | 96.40 | Unknown protein |  | - |
| Glyma.10g181700 | Gm10 | 41505696-41512460 | 40908884_G_A | Peak 3 | R1 | 86.17 | Casein kinase I | AT4G14340.1 | - |
| Glyma.10g182300 | Gm10 | 41561515-41570949 | 40908884_G_A | Peak 3 | R1 | 89.08 | COP1-interacting protein-related | AT5G43310.3 | - |
| Glyma.10g182400 | Gm10 | 41572844-41575499 | 40908884_G_A | Peak 3 | R1 | 89.22 | Protein phosphatase 2C family protein | AT3G23360.1 | - |

Chr is chromosome, Gen Pos is gene positions in Wm82.a1.v1 genome assembly, R8 is days to maturity, RL is reproductive length, R1 days to flowering, ^a^Tagging SNPs IDs start with “BARC_1.01_Gm” and the number of chromosome and the positions are based on Wm82.a1; ^b^ Average accuracies obtained by AccuTool. ^d^ Orthologs genes name in Arabidopsis. ^e^ Gene bank ID from reference sequences (RefSeqs) of the gene in *Glycine max.* available at NCBI website

| **Gene** | **Pos. (bp)^a^** | **Chr** | **Average Accuracy** | | | | | | |
| --- | --- | --- | --- | --- | --- | --- | --- | --- | --- |
|  |  |  | **Gm10**  **41455680^a^** | **Gm04**  **17228343^a^** | **Gm04**  **17075267^a^** | **Gm04**  **40009617^a^** | **Gm04**  **40276263^a^** | **Gm04**  **40151473^a^** | **Gm04**  **40218961^a^** |
| **E1La** | 36,758,125  36,758,770 | 4 |  | 78.2 | 79.3 | 73 | 83 | 83 | 83 |
| **E1Lb** | 26,120,011  26,120,532 | 4 |  | 50 | 30 | 50 | 50 | 50 | 50 |
| **E2 /Glyma.10g221500** | 45,294,735  45,316,121 | 10 | 43.4 |  |  |  |  |  |  |
| **E8** | 9,337,214  9,341,731 | 4 |  | 73 | 73 | 70 | 71 | 66 | 65 |

Table S9. Correspondence between tagging SNPs and known E genes measured by average accuracy obtained from AcuTool.

**^a^** Gene and SNP positions are based on Wm82.a2.v1 genome assembly.


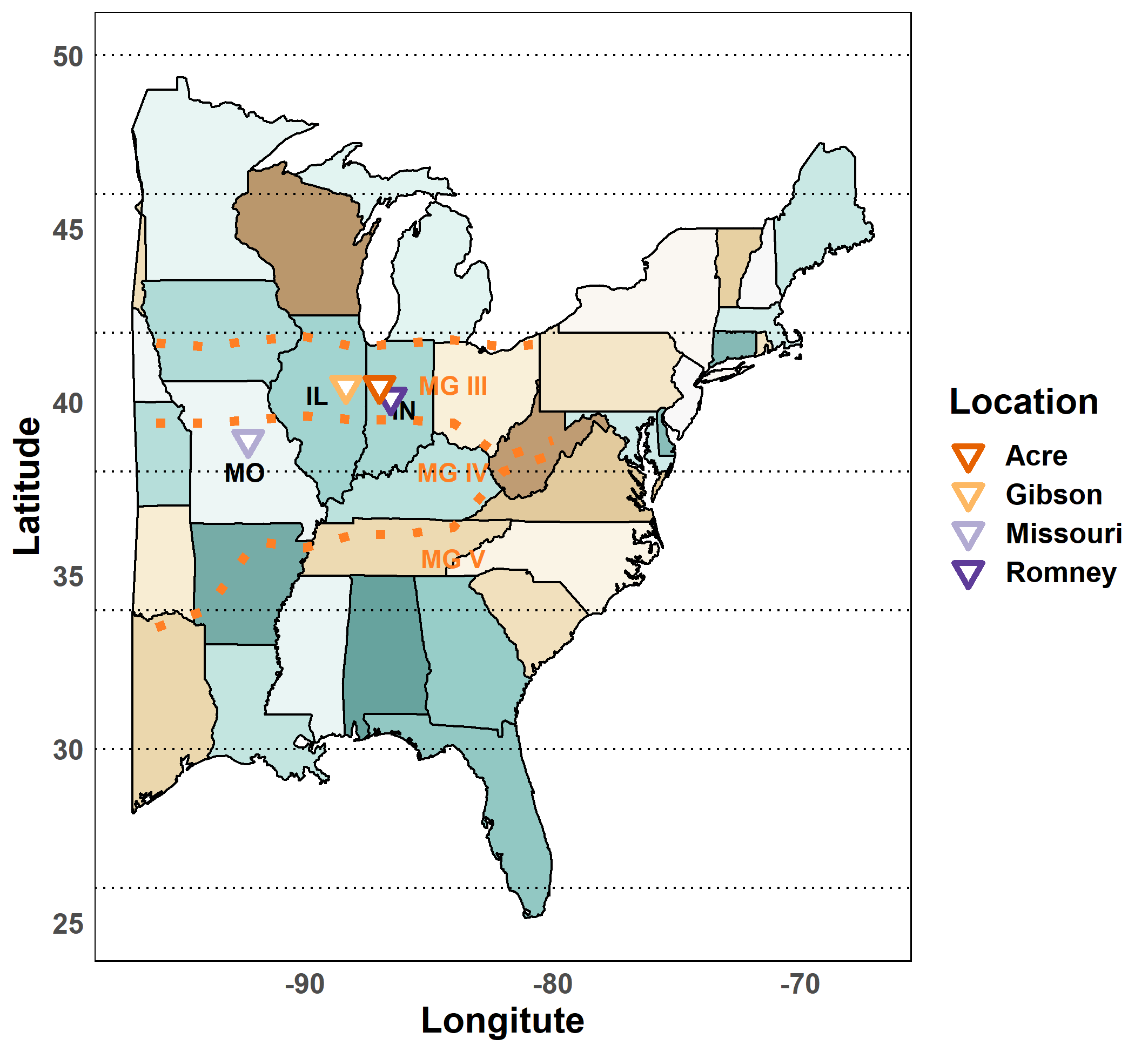


Figure S1. The map shows the four locations where flowering (R1), maturity (R8), and reproductive length (RL) measurements were made from 2017 to 2020. Orange squares delimit MG III and IV adaptation regions.


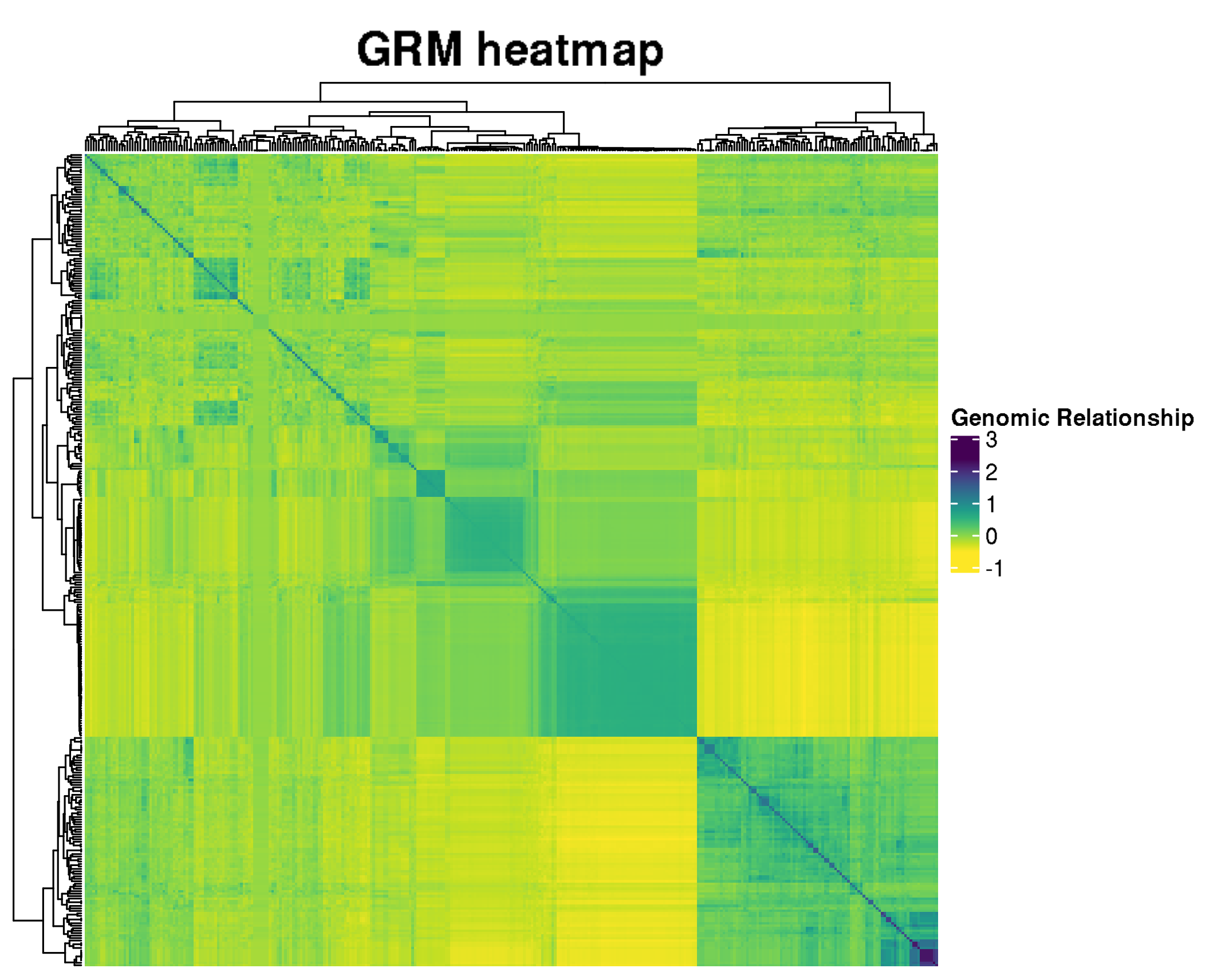


Figure S2. Heatmap from a genomic relationship matrix of the three hundred twenty-nine *G.* max USDA accession used in this study.


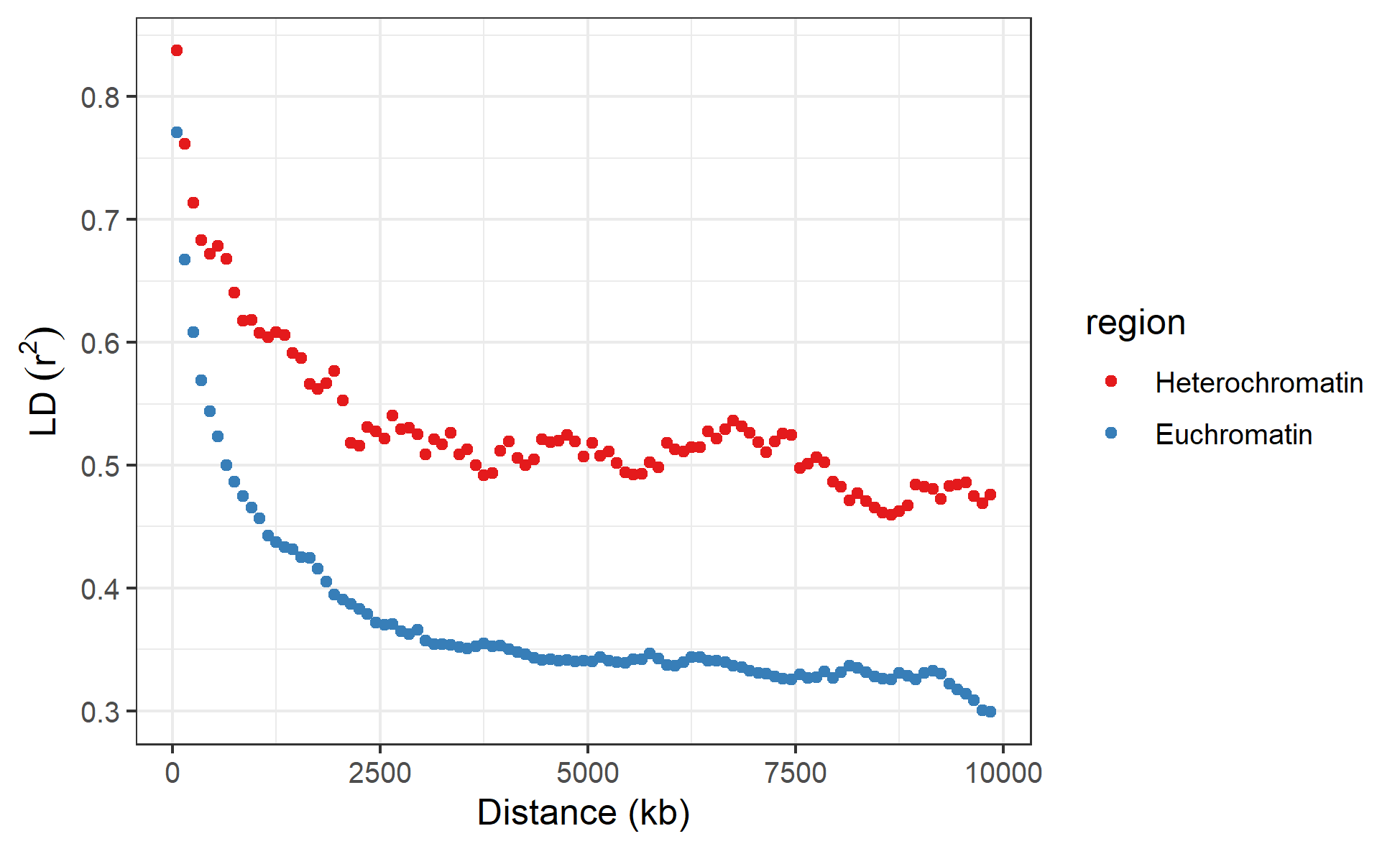


Figure S3. Plots of average squared correlation of allele frequencies (*r^2^*) against distance across the whole genome from heterochromatin (red) and euchromatin regions (blue).


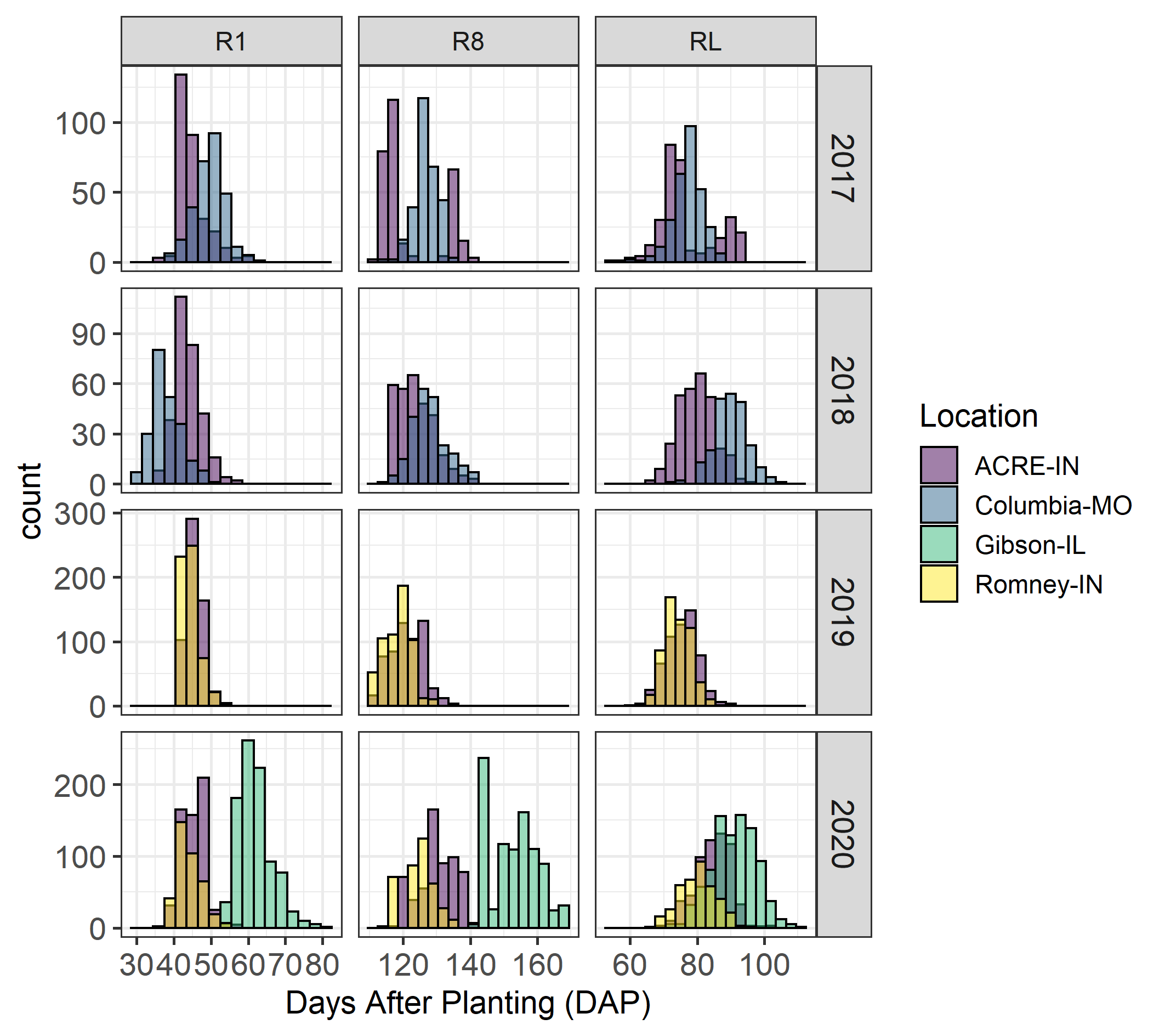


Figure S4. Histograms of the frequency distribution of the growth period traits flowering (R1), maturity (R8), and reproductive length (RL) for three hundred twenty-nine *G. max* accessions. Data are measurements of R1 and R8 in days after planting (DAP), and RL as the number of days between R1 and R8 from four years and four locations.


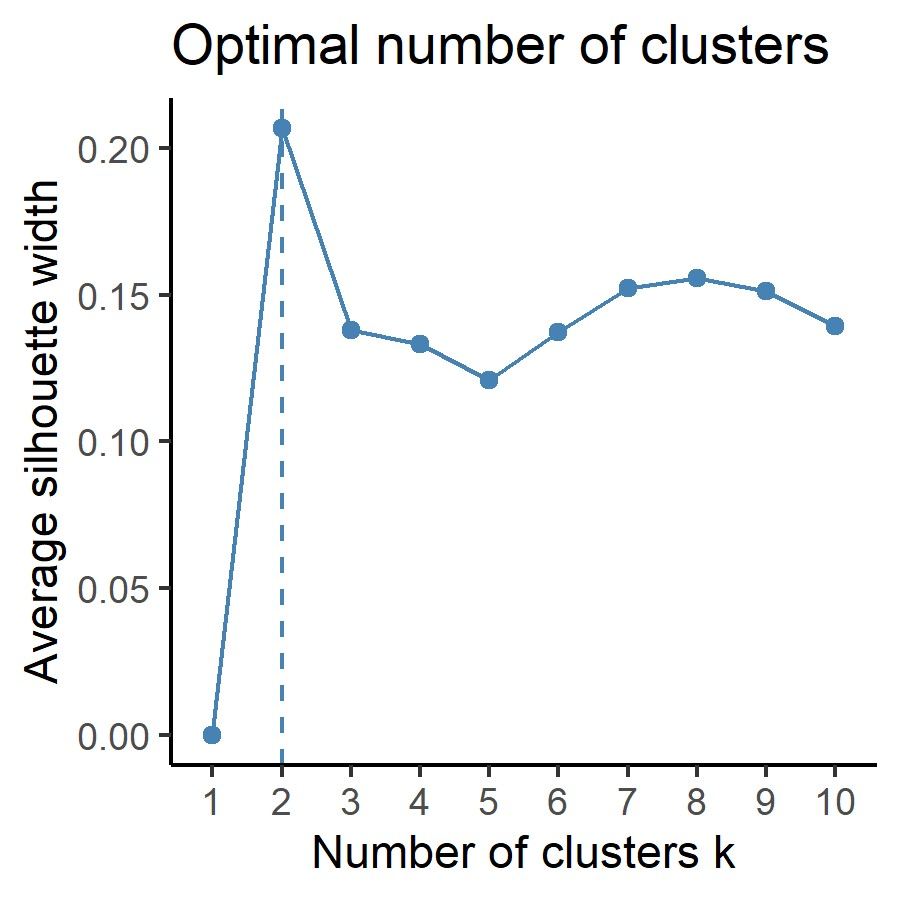


Figure S5. Optimal number of clusters by k-means using the average silhouette width


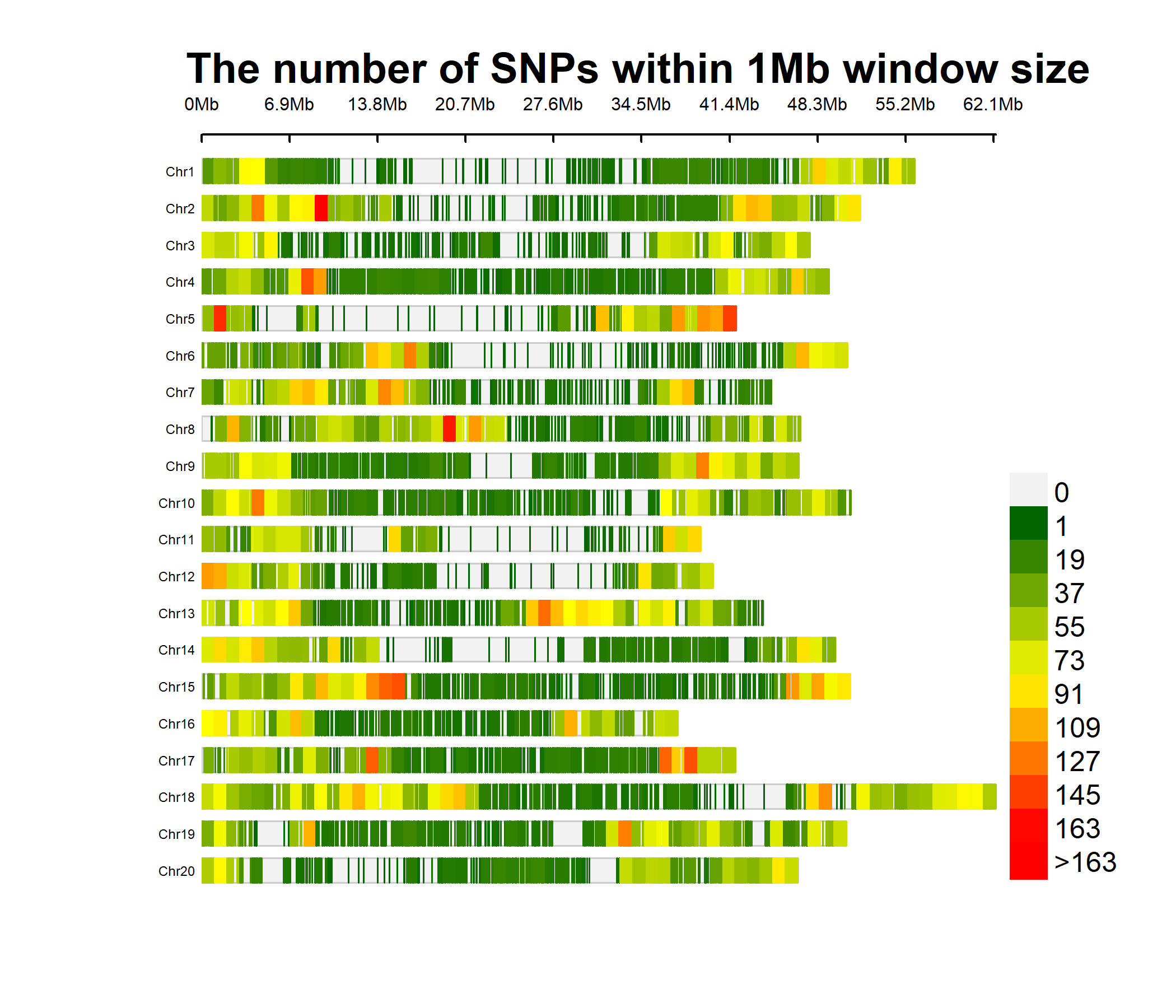


Figure S6. SNP density plot across 20 chromosomes of soybean representing the number of SNPs within 1 Mb window size


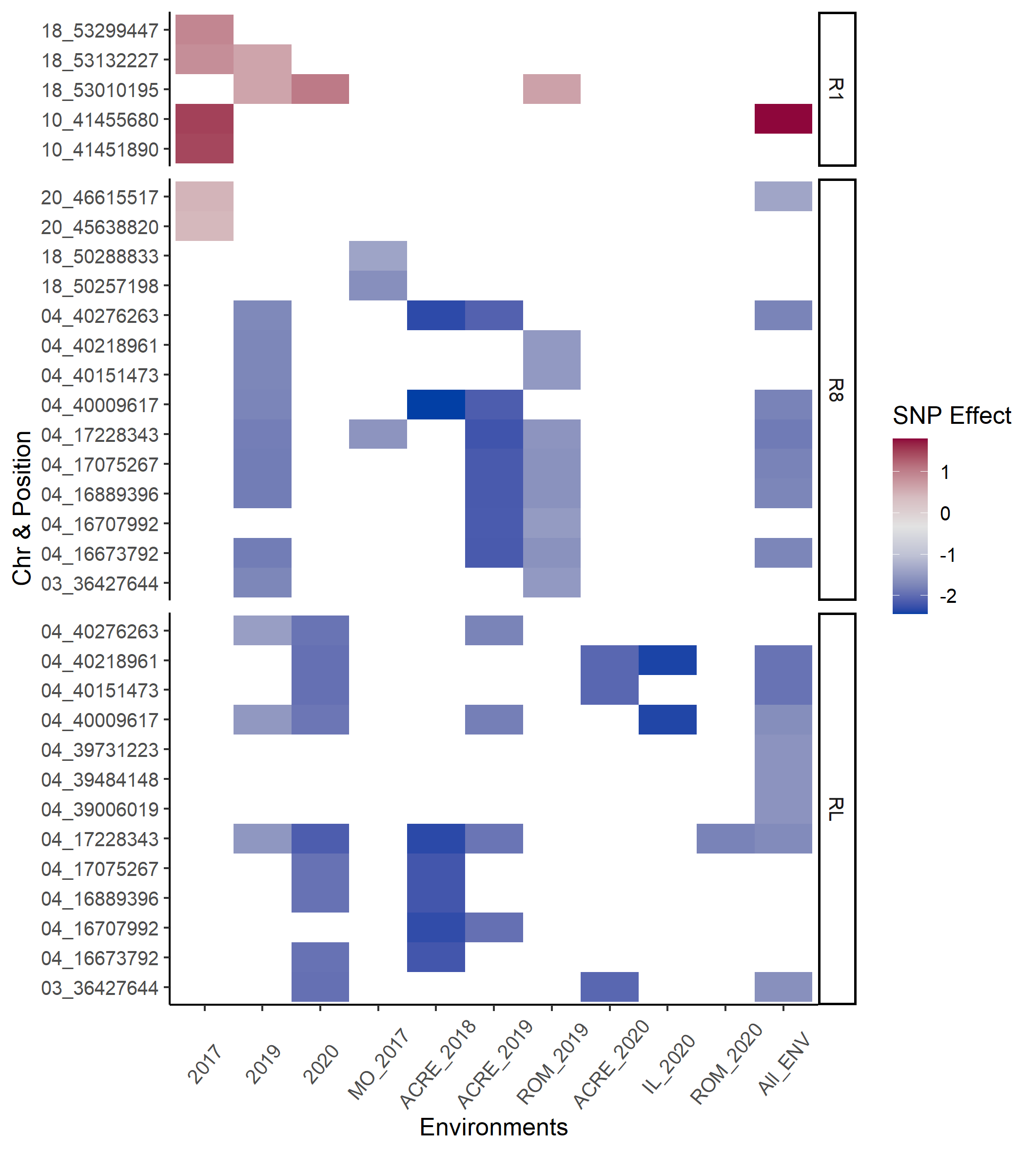


Figure S7. Significant SNPs from genome-wide association analysis by individual environments, years and across all environments. R1 is flowering time, R8 is maturity time, and RL is reproductive length. The plot is color coded by the effect of the SNPs for each trait. SNP positions are based on the Wm82.a2.v1 genome assembly.


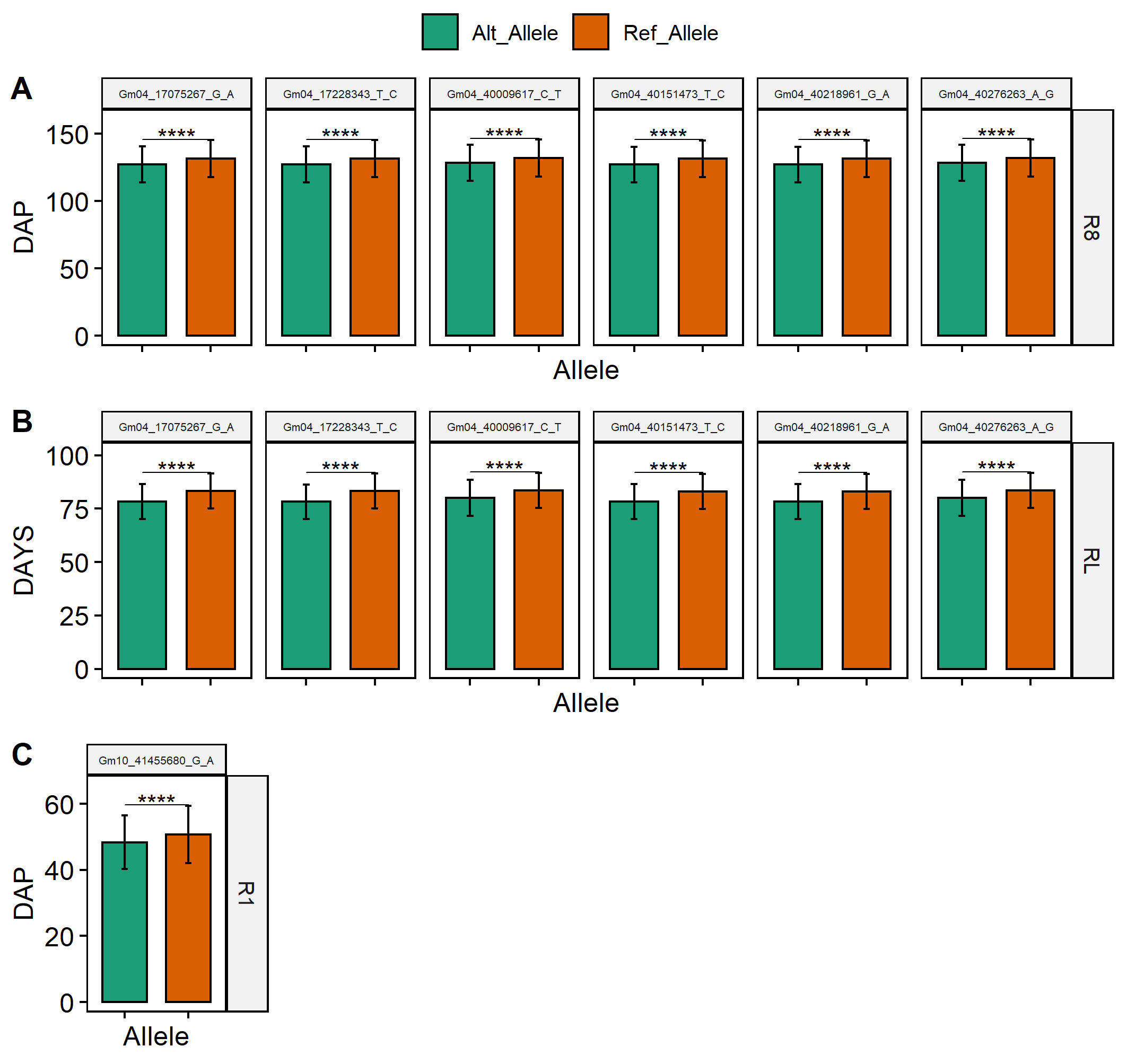


Figure S8. Phenotypic differences between lines carrying the reference allele (Ref_Allele) and the alternative allele (Alt_Allele) of the seven SNPs highly associated with R1, R8, and RL. Bar plots, show the differences in R8 (A), RL (B), and R1 (C). **** indicates significant differences at a *p*-value ≤ 0.0001 between the two groups. DAP is days after planting.
