# Supplementary material for: Genome-wide association study reveals GmFulb as candidate gene for maturity time and reproductive length in soybeans (Glycine max)
Source: PLoS One. 2024 Jan 19;19(1):e0294123. doi: 10.1371/journal.pone.0294123 (PMC10798547; doi:10.1371/journal.pone.0294123)
Supplement: Attachment — Submitted filename: Plos_one_review.docx [file pone.0294123.s019.docx]

The authors have performed GWAS analyses in soybean and documented some interesting finding. However, the paper is poorly drafted, and the findings are not well presented.

I have few suggestions,

1) Make title precise. Include the word "soybean" in the title.
2) Please narrow down the introduction section to your focused research question or hypothesis.
3) Line 161. Which package in R? Add a reference if applicable.
4) Avoid short forms in the abstract. Chr=Chromosome.
5) Throughout the manuscript, editing of English language required.
6) Line 245-246. How were the genes selected to be further analyzed? The statements are unclear.
7) Make discussion section short and precise. The explanation of results should go to the result section.
8) Line 281-282. Are you clear on the statement? Even 5,000 markers are sufficient. Please verify the statement and reference.
9) I suggest redrafting the GWAS section. It's a little confusing. Line 279-294.
